# Western diet-induced MASH in PWK/PhJ mice identifies disruptions in amino acid and sphingolipid metabolism contributing to cardiac dysfunction

**DOI:** 10.1038/s41467-026-73449-7

**Published:** 2026-05-20

**Authors:** Sandra Rodríguez-López, Miguel Pérez-Rodríguez, Alaa Badreddine, Rafael Calais Gaspar, Henrique J. Novaes Morgan, Ikki Sakuma, Giacomo V. G. von Alvensleben, Alejandro Alonso-Calleja, Stacia P. A. Everts, Nicolas-Enzo Suter, Giorgia Benegiamo, Christine Goepfert, Simone de Brot, José Manuel Villalba, Gerald I. Shulman, Kristina Schoonjans, Johan Auwerx

**Affiliations:** 1 Laboratory of Integrative Systems Physiology, École polytechnique fédérale de Lausanne, Lausanne, Switzerland; 2 Departamento de Biología Celular, Fisiología e Inmunología, Universidad de Córdoba, Campus de Excelencia Internacional Agroalimentario CeiA3, Córdoba, Spain; 3 Department of Internal Medicine, Yale University School of Medicine, New Haven, CT USA; 4 Department of Cellular & Molecular Physiology, Yale University School of Medicine, New Haven, CT USA; 5 Department of Physiology, Ribeirão Preto Medical School, University of São Paulo, Riberão Preto, Brazil; 6 Laboratory of Metabolic Signaling, Institute of Bioengineering, Ecole Polytechnique Fédérale de Lausanne, Lausanne, Switzerland; 7 COMPATH, Institute of Animal Pathology, University of Bern, Bern, Switzerland; 8 Histology Core Facility, Ecole Polytechnique Fédérale de Lausanne, Lausanne, Switzerland

**Keywords:** Molecular biology, Metabolic disorders

## Abstract

Metabolic dysfunction-associated steatotic liver disease (MASLD) and metabolic dysfunction-associated steatohepatitis (MASH) are liver disorders strongly associated with cardiovascular disease (CVD). The PWK/PhJ mouse strain is an emerging model for severe MASH, highly susceptible to Western diet (WD) and closely mimicking the clinical and molecular profile of human MASH. Here, we demonstrate that male and female PWK/PhJ mice develop hepatic fibrosis and cardiac dysfunction after 17 weeks of WD challenge. Elevated cholesterol levels and altered transcript profiles associated with translation and lipid metabolism characterize the early metabolic changes induced by WD. Chronic exposure to WD exacerbates hepatic lipid accumulation, inflammation, and fibrosis, while disrupting amino acid and mitochondrial metabolism. These alterations increase hepatic synthesis of ceramides and deoxy-ceramides, contributing to elevated sphingolipid levels in plasma and heart tissue. Collectively, these metabolic changes drive the development of MASH and significantly increase CVD risk. Our findings establish the PWK/PhJ strain as a robust model to study cardio-metabolic cross talk and identifying therapeutic targets for cardio-metabolic disorders.

## Abstract (web summary)

Metabolic liver disease is strongly linked to cardiovascular risk, but mechanisms connecting hepatic and cardiac pathology remain unclear. Here the authors show that PWK/PhJ mice develop diet-induced MASH with fibrosis and cardiac dysfunction, establishing this strain as a valuable model to dissect MASLD–CVD cross talk.

## Introduction

Metabolic dysfunction-associated steatotic liver disease (MASLD) and its most severe form, metabolic dysfunction-associated steatohepatitis (MASH), consist of a spectrum of conditions linked to metabolic risk factors like obesity, insulin resistance, and dyslipidemia. MASH is characterized by excess hepatic lipid deposition, inflammation and cellular damage, which can progress to fibrosis, cirrhosis and/or hepatocellular carcinoma^1,2^. The presence of fibrosis is the key predictor of prognosis and mortality, significantly increasing the risk of extrahepatic complications^3^, such as diabetes, chronic kidney disease (CKD), and cardiovascular disease (CVD), which is the leading cause of death among these patients^4,5^. Consequently, MASLD and MASH represent significant medical and socioeconomic burdens.

Recently, two different MASLD subtypes have been identified in humans, each characterized by distinct risks and outcomes^6^. The liver-specific MASLD subtype is genetically driven, characterized by elevated plasma liver enzymes, rapid liver damage progression, and a low risk of CVD and type 2 diabetes (T2DM). In contrast, cardiometabolic MASLD is characterized by higher rates of dyslipidemia, hypertension, and altered glucose homeostasis, leading to increased risks of CVD and T2DM. Additionally, cardiometabolic MASLD is characterized by the elevation of specific metabolites in plasma, including glycerophospholipids, sphingolipids, amino acid derivatives, and bile acid metabolites, which define a distinct metabolic signature linked to a high risk of MASH progression^6^.

Metabolomic studies have shown an association between circulating levels of branched-chain amino acids (BCAAs) and an increased risk of metabolic diseases, including MASLD. High serum BCAA levels, closely associated with early-stage liver fat accumulation, may result from insulin resistance and disrupted protein catabolism^7^. Skeletal muscle, with high BCAA aminotransferase (BCAT) activity, is the primary site for BCAA catabolism, while the liver, with very low BCAT levels, processes only a small fraction. While BCAAs have been positively linked to increased cardiometabolic risk, glycine and serine levels have been observed to decrease in metabolic diseases, including MASLD^8–10^. Additionally, the alanine-to-glycine ratio has emerged as a predictive biomarker for T2DM^11^.

Serine is an essential precursor for de novo sphingolipid synthesis, starting with its condensation with palmitoyl CoA in a reaction catalyzed by the enzyme serine palmitoyl transferase (SPT). Under certain conditions, SPT can also use alternative amino acids, such as alanine and glycine, as substrates^10,12–14^. The use of alanine and glycine instead of serine results in the production of toxic sphingolipid derivatives, deoxy-sphingolipids (DeoxySph) and deoxy-ceramides (DeoxyCer). Elevated levels of ceramides and their toxic derivatives are involved in several metabolic diseases^15,16^ and contribute to MASLD development by promoting lipotoxicity, insulin resistance, oxidative stress, and inflammation^10,17,18^. The liver is considered the main contributor to elevated circulating ceramide levels. After their synthesis, ceramides are exported through lipoproteins, which are then secreted into the bloodstream and can contribute to the development of atherosclerosis^17,18^. Consequently, plasma ceramides are emerging as important biomarkers for a variety of conditions, including MASH and atherosclerosis^19^.

The lack of a robust preclinical model that closely replicates the human phenotype has limited advances in understanding the chronic and complex pathophysiology of MASLD and MASH^20^. In previous studies, we identified the PWK/PhJ strain as a more effective model for studying MASH, exhibiting mitochondrial dysfunction, rapid fibrosis progression, and mimicking key features of the human pathology^21,22^. The PWK/PhJ strain addresses a critical limitation of the commonly used C57BL/6 J model, which is resistant to the development of extensive fibrosis. However, the cardiometabolic profile of PWK/PhJ mice during MASH progression has not been fully characterized. Since MASH is a well-recognized sexually dimorphic disease^23^, including both males and females in metabolic studies is essential for comprehensive analysis. Additionally, housing temperature plays a crucial role in shaping outcomes across various mouse models of human diseases, with particular relevance to metabolic disorders^24^.

In this study, we first evaluated the cardiometabolic phenotype of male and female PWK/PhJ mice under different housing temperatures following a Western diet (WD) challenge. Our findings demonstrate that housing temperature significantly affects body weight, energy expenditure, and glucose homeostasis, emphasizing the importance of considering environmental factors beyond diet in metabolic studies. Notably, while female mice are typically resistant to diet-induced obesity and MASLD/MASH development, PWK/PhJ female mice exhibited liver fibrosis progression similar to males under WD. Additionally, both sexes developed cardiac dysfunction, with females exhibiting a more pronounced phenotype. Next, we conducted a comprehensive longitudinal study uncovering both early and late events in the progression of MASLD/MASH disease. Early events included increased plasma cholesterol levels and changes in liver transcript profiles, while late events involved liver fibrosis, impaired amino acid, mitochondrial and sphingolipid metabolism. Mitochondrial dysfunction is a hallmark of advanced MASH and, together with abnormal sphingolipid metabolism, contributes to ceramide-driven inflammation and fibrosis^25^. Increased de novo ceramide synthesis and accumulation in the liver, along with elevated ceramides and sphingomyelin in plasma and heart, suggest enhanced export of sphingolipid-rich lipoproteins from a compromised liver. This contributes to systemic lipid imbalance and promotes cardiac ceramide buildup, a risk factor for heart dysfunction^26^. Our study identifies an imbalance in hepatic amino acid and sphingolipid metabolism in PWK/PhJ mice that perturbs cardio-metabolic signaling in MASH and hence reveals potential therapeutic targets for cardio-metabolic diseases.

## RESULTS

### Sex-dependent metabolic responses to housing temperature in PWK/PhJ mice influence liver and heart phenotypes

**Fig. 1:**
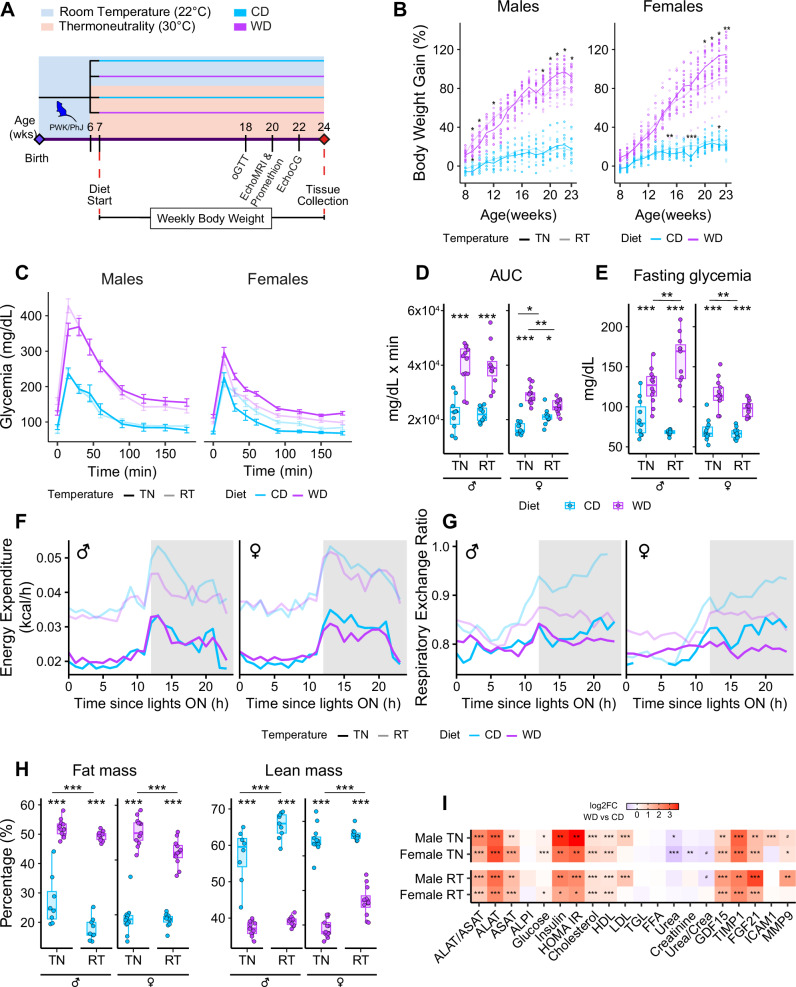
Effect of housing temperature in PWK/PhJ mice. **A** Illustration of the experimental pipeline. Male and female PWK/PhJ mice were housed either at thermoneutrality (TN) or room temperature (RT) and fed either a Western-style diet (WD) or a control diet (CD) for 17 weeks, beginning at 7 weeks of age. The in vivo phenotypes were assessed at the indicated time points. **B** Body weight gain curves expressed as a percentage of the starting body weight, with the line representing the median. TN-males, *n* = 8 CD, 12 WD; TN-females, *n* = 12 CD, 13 WD; RT-males, *n* = 9 CD, 11 WD; RT-females, *n* = 11 CD, 12 WD. **C** Oral glucose tolerance test (OGTT) curves. TN-males, *n* = 11 CD, 12 WD; TN-females, *n* = 12 CD, 13 WD; RT-males, *n* = 10 CD, 11 WD; RT-females, *n* = 11 CD, 12 WD**. D** Area under the curve (AUC) from OGTT. TN-males, *n* = 11 CD, 12 WD; TN-females, *n* = 12 CD, 13 WD; RT-males, *n* = 10 CD, 11 WD; RT-females, *n* = 11 CD, 12 WD**. E** Fasting glycemia measured after overnight fasting. TN-males, *n* = 11 CD, 12 WD; TN-females, *n* = 12 CD, 13 WD; RT-males, *n* = 10 CD, 11 WD; RT-females, *n* = 11 CD, 12 WD**. F** Energy expenditure (EE) (kcal/h). TN-males, *n* = 8 CD, 10 WD; TN-females, *n* = 10 CD, 10 WD; RT-males, *n* = 9 CD, 11 WD; RT-females, *n* = 11 CD, 12 WD. **G** Respiratory exchange ratio (RER). Lines indicate the median, and the white and shaded areas represent light and dark housing phases (12 h each), respectively. TN-males, *n* = 8 CD, 10 WD; TN-females, *n* = 10 CD, 10 WD; RT-males, *n* = 9 CD, 11 WD; RT-females, *n* = 11 CD, 12 WD**. H** Percentage of fat mass and lean mass. TN-males, *n* = 8 CD, 12 WD; TN-females, *n* = 12 CD, 13 WD; RT-males, *n* = 9 CD, 11 WD; RT-females, *n* = 11 CD, 12 WD**. I** WD vs CD log2 fold changes of plasma analytes. For GDF15, TIMP1 and FGF21: TN-males, *n* = 8 CD, 11 WD; TN-females, *n* = 10 CD, 9 WD; RT-males, *n* = 9 CD, 11 WD; RT-females, *n* = 10 CD, 10 WD. For ICAM1 and MMP9: TN-males, *n* = 6 CD, 6 WD; TN-females, *n* = 6 CD, 6 WD; RT-males, *n* = 6 CD, 6 WD; RT-females, *n* = 6 CD, 5 WD. For the remaining plasma markers: TN-males, *n* = 8 CD, 12 WD; TN-females, *n* = 12 CD, 13 WD; RT-males, *n* = 9 CD, 11 WD; RT-females, *n* = 11 CD, 12 WD. For (**D**, **E**, **H**), results are shown as box-and-whisker plots. The lower and upper hinges correspond to the first quartile (25th percentile) and third quartile (75th percentile), with the median represented by a line in the center. The whiskers show the minimum and maximum values in the data. Points beyond the whiskers are outliers, plotted individually. Statistical analysis was performed using a two-way ANOVA followed by Tukey’s post hoc test. For **B** and **I**, statistical analysis was conducted using a two-sided Student’s *t*-test with Benjamini–Hochberg adjusted *p*-values. ^#^*P* < 0.1, *P < 0.05, **P < 0.01, ***P < 0.001. Source data are provided as a Source Data file.

To investigate the impact of housing temperature on diet-induced MASH in PWK/PhJ mice, male and female mice were fed either chow diet (CD) or WD for 17 weeks and housed at room temperature (RT) or thermoneutrality (TN) (Fig. 1A). We observed an increase in body weight gain in WD-fed mice housed at TN compared to those at RT starting from week 19. In contrast, CD-fed mice showed no significant differences in body weight by the end of the experiment (Fig. 1B). Given the role of obesity in driving insulin resistance^27^, we measured glucose tolerance (OGTT test) after 11 weeks of dietary intervention. Both male and female animals showed glucose intolerance on WD compared to CD. No temperature-dependent differences were observed in male animals (Fig. 1C). In contrast, female mice under CD showed a higher area under the curve (AUC) for OGTT in RT compared to TN, but lower AUC in RT compared to TN in WD (Fig. 1C, D). Fasting plasma glucose (FPG) levels were higher in the WD groups. In males, we observed elevated FPG in RT compared to TN, whereas in females, FPG was higher in TN compared to RT (Fig. 1E). Together, our data indicate sex differences in metabolic adaptation to environmental and WD challenges.

Adaptation to housing conditions affects temperature regulation, impacting, among others, energy expenditure (EE)^28–31^. As expected, both male and female mice showed lower EE when housed at TN during both the light and dark phases compared to RT housing (Fig. 1F). EE is directly related to VO_2_ consumption and VCO_2_ production (Fig. S1A), which also showed higher levels in RT groups. A respiratory exchange ratio (RER) close to 0.8 in TN animals reflects a mixed fuel utilization for energy. In contrast, an RER between 0.9–1 suggests more reliance on carbohydrate metabolism, especially during the night phase in CD-RT mice (Fig. 1G). EE is strongly influenced by body composition. We observed increased fat mass in WD and lean mass in CD-fed animals, independent of sex. However, in males, the impact of TN compared to RT on fat and lean mass was observed only in CD-fed animals, while in females, the effects were limited to WD-fed animals (Fig. 1H).

MASLD and MASH are characterized by alterations in plasma markers, particularly those associated with liver damage, lipid levels, and inflammation^32,33^. Circulating liver enzymes ALAT and ASAT, key indicators of liver damage, were significantly higher in WD-fed animals (Figs. 1I, S1B). We observed reduced plasma urea levels in animals housed at TN, indicating compromised liver function and alterations in nitrogen metabolism (Figs. 1I, S1B). Additionally, TN groups exhibited higher levels of total cholesterol in plasma compared to the RT groups (Figs. 1I, S1B). A panel of cytokines, chemokines, and other plasma factors associated with MASH was measured in plasma samples. However, most of these markers were below the limit of detection (Supplementary Data 1). Notably, plasma levels of GDF15, TIMP1, and FGF21 were consistently elevated in WD-fed mice, regardless of the housing temperature (Figs. 1I, S1B). These circulatory markers are released during MASH progression in response to hepatic stress and inflammation, serving as indicators of disease progression^34–37^.

**Fig. 2:**
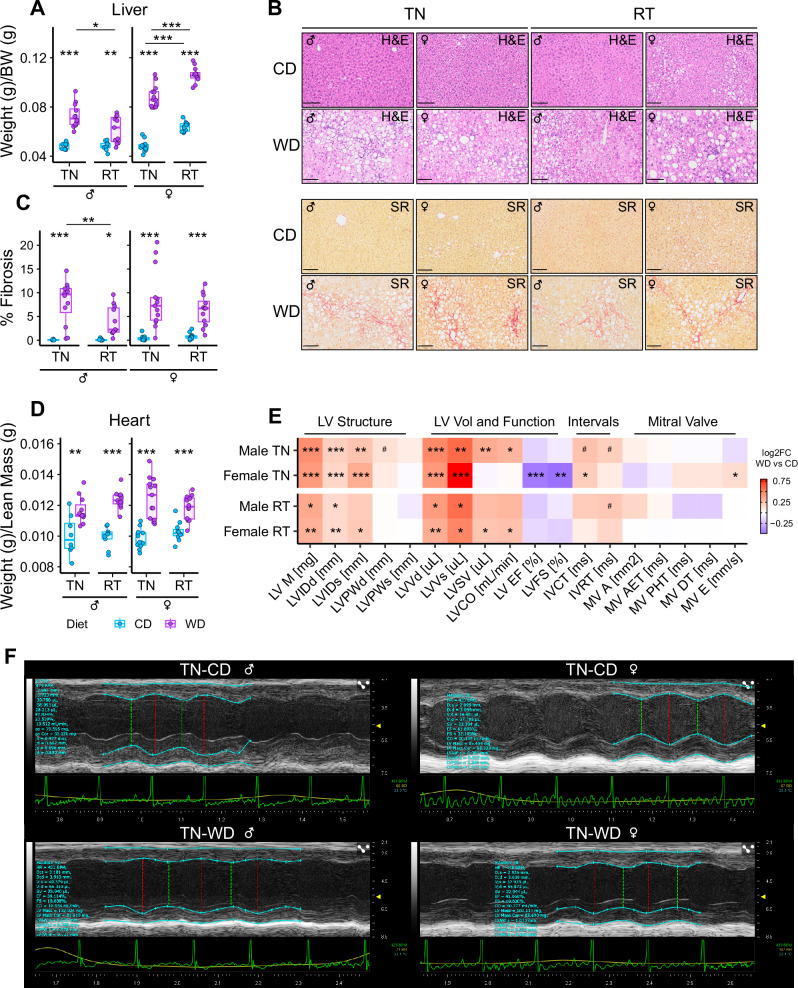
Thermoneutrality housing exacerbates MASH severity and heart dysfunction. **A** Liver weight normalized to body weight. TN-males, *n* = 8 CD, 12 WD; TN-females, *n* = 12 CD, 13 WD; RT-males, *n* = 9 CD, 11 WD; RT-females, *n* = 11 CD, 12 WD. **B** Representative H&E and Sirius Red (SR) staining images of formalin-fixed liver sections. Scale Bar = 100 µm. **C** Percentage of fibrosis quantified as a positive area for SR staining. TN-males, *n* = 8 CD, 12 WD; TN-females, *n* = 12 CD, 13 WD; RT-males, *n* = 9 CD, 11 WD; RT-females, *n* = 11 CD, 12 WD. **D** Heart weight normalized to lean mass. TN-males, *n* = 8 CD, 12 WD; TN-females, *n* = 12 CD, 13 WD; RT-males, *n* = 9 CD, 11 WD; RT-females, *n* = 11 CD, 12 WD. **E** WD *vs*. CD log2 fold changes of echocardiography parameters. TN-males, *n* = 8 CD, 12 WD; TN-females, *n* = 12 CD, 13 WD; RT-males, *n* = 9 CD, 11 WD; RT-females, *n* = 11 CD, 12 WD. **F** Representative M-mode echocardiography images from the TN groups. For (**A**, **C**, **D**), results are shown as box-and-whisker plots. The lower and upper hinges correspond to the first quartile (25th percentile) and third quartile (75th percentile), with the median represented by a line in the center. The whiskers show the minimum and maximum values in the data. Points beyond the whiskers are outliers, plotted individually. Statistical analysis was performed using a two-way ANOVA followed by Tukey’s post hoc test. For **E**, statistical analysis was conducted using a two-sided Student’s *t*-test with Benjamini–Hochberg adjusted *p*-values. ^#^*P* < 0.1, *P < 0.05, **P < 0.01, ***P < 0.001. Source data are provided as a Source Data file.

We next evaluated the effect of WD on the tissue mass. Kidney and skeletal muscle tissues exhibited lower normalized weight in WD groups, while the liver, heart, and abdominal fat (EpiWAT) showed increased relative weight (Figs. 2A, D, S2A). Males exhibited a higher liver-to-body weight ratio as well as greater absolute liver weight in TN compared with RT, but only in WD (Figs. 2A, S2B). In contrast, females showed increased liver-to-body weight ratio under RT in both CD- and WD-fed animals (Fig. 2A). Histological analysis of the liver revealed sex- and temperature-dependent differences in fibrosis, steatosis, and inflammation. In males, fibrosis percentage per tissue area was higher under TN compared to RT conditions **(**Fig. 2B, C**)**, although no significant differences were observed in fibrosis scores (TN: 1.83, RT:1.55) (Fig. S2D). In females, fibrosis percentage per tissue area did not differ between housing temperatures **(**Fig. 2B, C**)**, but fibrosis scores were significantly higher in RT (2.08) than in TN (1.77) **(**Fig. S2D**)**. Liver steatosis increased under WD, with no differences observed between housing conditions in the percentage of macrosteatosis **(**Fig. S2C**)**. When considering steatosis score—which accounts for both micro- and macrosteatosis—females under TN showed increased steatosis levels **(**Fig. S2D**)**. Inflammation was elevated in females in both CD and WD **(**Fig. S2D**)**. No differences in total NAS scores were observed between conditions RT and TN under CD, whereas females in WD-RT showed increased NAFLD activity scores (NAS) compared to females in WD-TN, driven primarily by higher inflammation **(**Fig. S2D**)**. Sex comparisons revealed that females accumulated more steatosis in CD under both temperatures than males. Overall, RT increased all NAS score parameters in females compared to males **(**Fig. S2D**)**. Fibrosis scores were also higher in females under RT, whereas no sex differences in NAS or fibrosis were observed under WD-TN **(**Fig. S2D**)**. Fibrosis marker expression was also evaluated by Western blot analysis (Fig. S3A, B). Taken together, these results indicate that TN induces a similar MASH phenotype in both males and females, whereas RT exacerbates the phenotype in females.

MASLD and MASH are strongly linked to CVD, being the leading cause of mortality in these patients^4,5^. Standard lab temperatures (20–22 °C) induce cold stress in mice^38–40^, affecting blood pressure and heart rate, making thermoneutral housing essential for metabolic studies linked to heart function. Echocardiography showed that WD led to cardiac hypertrophy (Figs. 2D, S2B) and left ventricular (LV) dilatation (LV M) in both male and female mice (Figs. 2E, F, S2D). Females housed at TN also exhibit mild LV systolic dysfunction (reduced LV EF) and diastolic dysfunction (elevated MV E) (Figs. 2E, F, S2D).

### WD induces liver transcript and protein signatures of MASH but has minimal effects on the heart

**Fig. 3:**
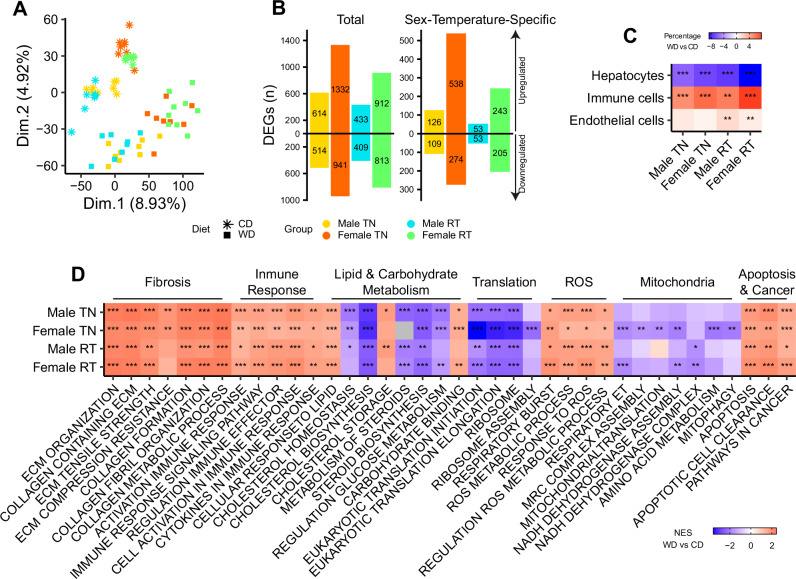
Liver transcriptome response to different housing temperatures. **A** PCA of CPM normalized liver gene expression data. **B** Total (left) and sex-temperature-specific (right) number of genes significantly up- or downregulated upon WD compared to CD in the liver for each experimental group. **C** Percentages of hepatocytes, immune cells, and endothelial cells in the liver samples, estimated by single-cell deconvolution analysis. **D** Gene Set Enrichment Analysis (GSEA) results for representative gene sets relevant for MASH. TN-males, *n* = 8 CD, 9 WD; TN-females, *n* = 8 CD, 8 WD; RT-males, *n* = 7 CD, 8 WD; RT-females, *n* = 7 CD, 9 WD. For **C** and **D**, statistical analysis was conducted using a two-sided Student’s *t*-test with Benjamini–Hochberg adjusted *p*-values. *P < 0.05, **P < 0.01, ***P < 0.001. Source data are provided as a Source Data file.

To explore the molecular mechanisms underlying sex differences in response to WD at different temperatures, we performed transcriptomic BRB-seq analysis on liver tissue. Principal component analysis (PCA) of gene expression showed a clear separation between the CD and WD groups (Fig. 3A). However, sex was a stronger determinant than temperature, as clusters specific to sex emerged, regardless of diet (Fig. 3A). Females exhibited the highest number of differentially expressed genes (DEGs), particularly those at TN (Fig. 3B). Males showed nearly half of DEGs compared to females both at TN and RT, indicating a stronger effect of WD on the liver transcriptomes of female mice. Additionally, females at TN had the highest number of sex-temperature-specific genes (Figs. 3B, S4A). This suggests that sex-specific responses to the diet are influenced by temperature, which emphasizes the need to consider both sex and environmental factors in gene expression studies. Interestingly, only 399 genes were common across all WD groups (Fig. S4B).

Next, we analyzed the impact of the WD on liver cellular composition estimated using single-cell deconvolution^41^. We found an increase in the proportion of immune cells in all groups, especially in females, along with a decrease in the proportion of hepatocytes. Surprisingly, in the RT groups, we observed an increase in endothelial cells, a pattern not seen in the TN groups (Figs. 3C, S4C). This could suggest potential differences in capillary formation or inflammatory responses between the two temperature conditions.

To determine the pathways underlying the diet-induced transcriptional changes within sex and temperature groups, we performed gene set enrichment analysis (GSEA) (Fig. 3D). Pathways related to fibrosis, immune response, reactive oxygen species (ROS), apoptosis, and cancer were positively enriched, whereas negative enrichment was observed for pathways linked to carbohydrate and lipid metabolism, translation, and mitochondrial function. Sex-specific differences showed positively enriched cholesterol storage pathways in males, and positively enriched carbohydrate binding and negative enrichment of mitochondrial pathways in females.

Given the observed increase in plasma cholesterol and the negative enrichment of GSEA results in pathways related to cholesterol synthesis and homeostasis, we examined individual genes related to cholesterol and lipid metabolism to identify specific drivers of this dysregulation. SREBP-2 (sterol regulatory element-binding protein 2) is the transcription factor that controls cholesterol homeostasis^42^. Under WD, elevated cellular cholesterol triggers a negative feedback loop, reducing SREBP-2 levels and consequently downregulating genes of the mevalonate pathway (Fig. S4D), which are essential for cholesterol synthesis. Conversely, *Sptlc3*, encoding a subunit of the rate-limiting enzyme in ceramide de novo synthesis, and *Smpd3,* encoding the enzyme responsible for hydrolyzing sphingomyelin into ceramides, were both upregulated (Fig. S4D). Increased ceramide levels promote the progression of MASLD to MASH and contribute to the development of atherosclerotic plaques^15,19,43^.

**Fig. 4:**
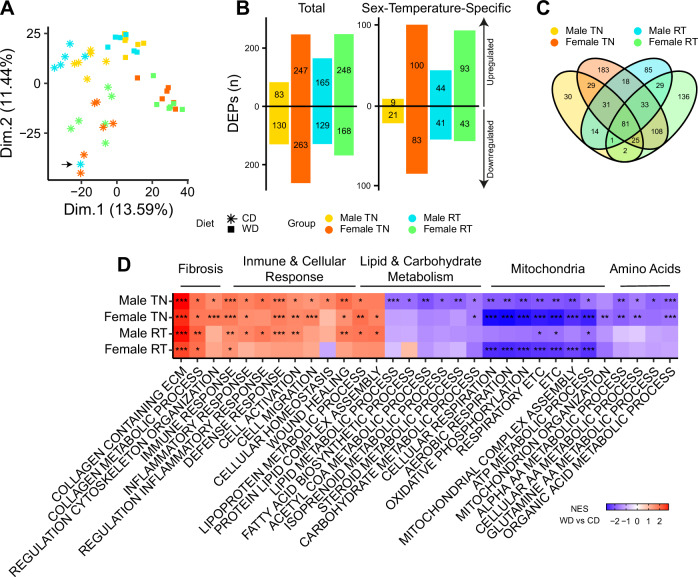
Proteomics analysis of liver tissues under different housing temperatures. **A** PCA on vsn-normalized LFQ intensity data from liver samples. The sample indicated by the black arrow was removed from downstream analysis. **B** Total (left) and sex-temperature-specific (right) number of proteins significantly up- or downregulated upon WD compared to CD in the liver for each experimental group. **C** Venn diagram of liver proteomics showing the number of proteins on WD overlapping between the four groups. **D** Gene Set Enrichment Analysis (GSEA) was performed on the proteomics data. The results show the representative protein sets relevant for MASH. TN-males, *n* = 6 CD, 6 WD; TN-females, *n* = 6 CD, 6 WD; RT-males, *n* = 5 CD, 6 WD; RT-females, *n* = 6 CD, 6 WD. For **D**, statistical analysis was conducted using a two-sided Student’s *t*-test with Benjamini–Hochberg adjusted *p*-values. *P < 0.05, **P < 0.01, ***P < 0.001. Source data are provided as a Source Data file.

To validate our transcriptomic findings, we performed proteomic profiling of the liver. Similar to the transcriptomics data, PCA of normalized protein abundance clearly separated CD and WD groups, with sex having a stronger effect than temperature (Fig. 4A). Females exhibited higher number of differentially expressed proteins (DEPs) than males in both TN and RT (Fig. 4B), with 81 proteins shared across all WD groups (Fig. 4C). GSEA results revealed positive enrichment for pathways related to fibrosis, immune and cellular response, while negative enrichment was observed for pathways associated with lipid metabolism, mitochondria, and amino acids. Notably, TN differed from RT by showing stronger negative enrichment of pathways related to amino acid metabolism (Fig. 4D).

Considering the strong correlation between our morpho-functional liver data and RNA sequencing results, we applied a similar approach to study the transcriptional changes underlying altered heart function. PCA of BRB-seq data obtained from whole hearts showed minimal separation between the CD and WD groups. In contrast to liver tissue, there was no clear distinction between males and females, nor between the temperature groups (Fig. S5A). This suggests minimal changes in transcript levels in the heart tissue. To confirm this, we analyzed the DEGs between diets in each group. Similar to the liver, females showed more DEGs than males, although the number of genes was significantly lower compared to the liver (Fig. S5B), and only 39 DEGs were common among all WD groups (Fig. S5C). Similarly, single-cell deconvolution data did not show significant changes in the proportion of different cell types in any of the groups (Fig. S5D). GSEA showed and confirmed minimal diet effect differences between sex and temperature groups. In males at TN, we identified negative enrichment of DNA binding and repair pathways, while in females, we observed negative enrichment of protein synthesis and rRNA processing. Positive enrichment of pathways related to lipid and fatty acid metabolism was observed for RT but not in TN groups (Fig. S5E). No specific pathway was found to be enriched and overlap among all the groups. Proteomic analysis of heart was performed to determine if the minimal changes in transcript levels were also reflected at the protein level. PCA showed little separation among the groups (Fig. S6A), consistent with the transcriptomic data, and only a few proteins were differentially expressed, with males at TN showing the fewest (Fig. S6B). Haptoglobin (HP) was the only protein consistently upregulated across all groups (Fig. S6C). GSEA indicated that no pathways were common to all groups, with females showing higher diet-associated alterations compared to males (Fig. S6D). These results suggest less pronounced transcript and protein changes in the heart compared to the liver. Alternatively, RNA and protein extraction from the whole heart may have masked expression changes specific to the LV due to heart tissue heterogeneity^44–46^.

### Transcript response to WD matches with downregulation of amino acid metabolism seen in human MASH

**Fig. 5:**
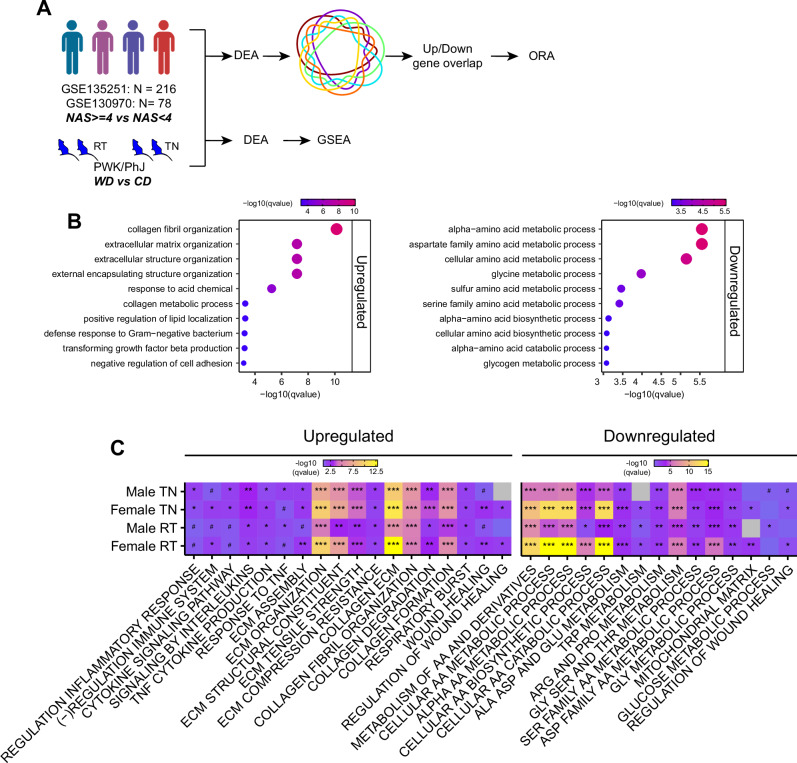
Mouse liver transcriptomic signatures correlate with MASLD/MASH severity in humans, characterized by suppressed amino acid metabolism pathways. **A** Illustration of the bioinformatic pipeline. Human icons were created in BioRender. Auwerx, J. (2026) https://BioRender.com/m700e43. Differential expression analysis (DEA) was performed on the mouse and human BRB-seq and RNA-seq data, respectively, followed by Over-Representation Analysis (ORA) on overlapping up- or downregulated genes, or gene set enrichment analysis (GSEA). For humans, the contrast NAS score ≥ 4 vs NAS < 4 was used for DEA, and the DEGs intersected with the ones obtained from the WD vs CD contrast in PWK/PhJ mice. **B** Top 10 enriched gene ontology (GO)-terms associated with biological processes among the up- or downregulated genes common to mouse and human. The dot size indicates significance (−log_10_[qvalue]). **C** Representative ORA-enriched gene sets for the up- and downregulated genes. TN-males, *n* = 8 CD, 9 WD; TN-females, *n* = 8 CD, 8 WD; RT-males, *n* = 7 CD, 8 WD; RT-females, *n* = 7 CD, 9 WD; GSE135251, *n* = 68 NAS < 4, *n* = 148 NAS > = 4; GSE130970, *n* = 36 NAS < 4, *n* = 42 NAS > = 4. Adjusted *q*-values: ^#^*Q* < 0.1, *Q < 0.05, **Q < 0.01, ***Q < 0.001. Source data are provided as a Source Data file.

To evaluate the clinical relevance of the PWK/PhJ susceptibility to MASH, we examined the overlap with human MASH signatures. We selected two publicly available datasets (GEO accession number GSE135251 and GSE130970)^47,48^, which include liver gene expression with available NAS scoring. We intersected the changes in transcript levels obtained from the contrast between subjects with a NAS score ≥ 4 vs NAS < 4 in humans, with the ones obtained by comparing WD vs CD in PWK/PhJ mice (Fig. 5A). We found 30 upregulated genes overlapping across all contrasts, with only 8 being downregulated (Fig. S7A). Using the jointly up-regulated genes, we identified enriched pathways related to extracellular matrix (ECM) organization, collagen synthesis, lipid metabolism, and cell adhesion. The overlapping downregulated genes resulted in an enrichment of pathways mainly linked to amino acid metabolism (Fig. 5B). Next, we performed overrepresentation analysis (ORA) on the DEGs overlapping between both human datasets and each of the mouse group, to functionally compare the transcriptional diet effect within specific experimental conditions and the onset/severity of MASH in humans. Across all groups, the overlapping upregulated genes showed significant enrichment for terms related to fibrosis, collagen, respiratory bursts, and inflammation, whereas for the overlapping downregulated genes we identified pathways associated with amino acid metabolism (Fig. 5C).

To shift our analysis from a gene-focused approach to a global view, we compared the global expression changes based on their biological functions by analyzing GSEA results across species. We identified conserved positive enrichment of pathways related to inflammation, fibrosis, and tissue remodeling in both humans and PWK/PhJ mice. We also identified conserved negative enrichment of cholesterol, steroid, and translation pathways (Fig. S7B). Our findings suggest comparable disease-related transcriptional signatures when aligned with human data sets between male and female PWK/PhJ mice.

### Male and female mice respond differently to acute and chronic WD challenge

To explore the progression from MASLD to MASH and its impact on metabolism, liver and heart, we performed a longitudinal study under TN conditions. This allowed us to gain valuable insights into the natural course of disease development.

**Fig. 6:**
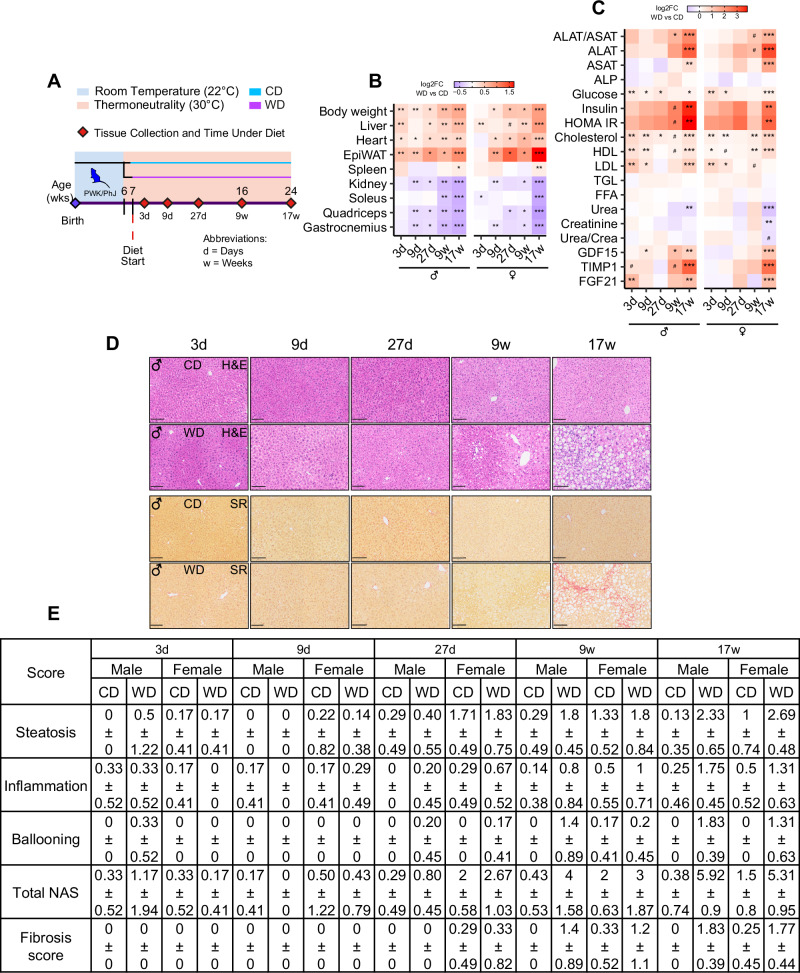
Longitudinal phenotyping of PWK/PhJ mice housed at TN. **A** Illustration of the experimental pipeline. Male and female PWK/PhJ were housed at TN and fed either WD or CD. Tissues were collected at 3, 9, 27 days and 9 weeks after starting the diet. Data collected after 17 weeks of the diet from the previous study (Fig. 1A) were used as the final time point to examine the diet’s effects over time. **B** Log2 fold changes for body weight and tissue weight at sacrifice, comparing WD to CD. Tissue weights were normalized to body weight (g/g), except the heart, shown as total weight (g). 3 d: TN-males, *n* = 6 CD, 6 WD; TN-females, *n* = 6 CD, 6 WD; 9 d: TN-males, *n* = 6 CD, 6WD; TN-females, *n* = 6 CD, 6 WD; 27 d: TN-males, *n* = 7 CD, 5 WD; TN-females, *n* = 7 CD, 6 WD; 9w: TN-males, *n* = 7 CD, 5 WD; TN-females, *n* = 6 CD, 5 WD; 17w: TN-males, *n* = 8 CD, 12 WD; TN-females, *n* = 12 CD, 13 WD. **C** Log2 fold changes for plasma analytes, comparing WD to CD. For GDF15, TIMP1 and FGF21: 3 d: TN-males, *n* = 6 CD, 6 WD; TN-females, *n* = 6 CD, 6 WD; 9 d: TN-males, *n* = 6 CD, 6WD; TN-females, *n* = 6 CD, 6 WD; 27 d: TN-males, *n* = 7 CD, 5 WD; TN-females, *n* = 7 CD, 6 WD; 9w: TN-males, *n* = 7 CD, 5 WD; TN-females, *n* = 6 CD, 5 WD; 17w: TN-males, *n* = 8 CD, 11 WD; TN-females, *n* = 10 CD, 9 WD. For the remaining plasma markers: 3 d: TN-males, *n* = 6 CD, 6 WD; TN-females, *n* = 6 CD, 6 WD; 9 d: TN-males, *n* = 6 CD, 6WD; TN-females, *n* = 6 CD, 6 WD; 27 d: TN-males, *n* = 7 CD, 5 WD; TN-females, *n* = 7 CD, 6 WD; 9w: TN-males, *n* = 7 CD, 5 WD; TN-females, *n* = 6 CD, 5 WD; 17w: TN-males, *n* = 8 CD, 12 WD; TN-females, *n* = 12 CD, 13 WD**. D** Representative H&E and SR staining images of formalin-fixed liver sections from male animals (Fig. S4 for females and CD45 staining images). Scale Bar = 100 µm. **E** Pathological scoring of liver H&E and SR-stained sections, presented as mean ± SD. Details of the scoring system, as well as the calculation and statistical analysis of the NAS and fibrosis scores, are provided in Supplementary Data 2. 3 d: TN-males, *n* = 6 CD, 6 WD; TN-females, *n* = 6 CD, 6 WD; 9 d: TN-males, *n* = 6 CD, 6WD; TN-females, *n* = 6 CD, 6 WD; 27 d: TN-males, *n* = 7 CD, 5 WD; TN-females, *n* = 7 CD, 6 WD; 9w: TN-males, *n* = 7 CD, 5 WD; TN-females, *n* = 6 CD, 5 WD; 17w: TN-males, *n* = 8 CD, 12 WD; TN-females, *n* = 12 CD, 13 WD. For (**B**, **C**, **E**), statistical analysis was conducted using a two-sided Student’s *t*-test with Benjamini–Hochberg adjusted *p*-values. ^#^*P* < 0.1, *P < 0.05, **P < 0.01, ***P < 0.001. For details on the p-values for the NAS and fibrosis scores, see Supplementary Data 2. Source data are provided as a Source Data file.

We collected tissue samples at 3, 9, 27 days and 9 weeks after starting the diet (Fig. 6A). Data from mice that had been under 17 weeks on the diet, collected from the experiment described above, were also used. We observed notable differences in body weight between the CD and WD groups after 3 days in males and 9 days in females (Fig. 6B). This also aligns with the tissue weights of the liver, heart, and epiWAT, which are the first to be affected by the diet (Figs. 6B, S8A). Decreases in kidney and muscle mass occurred later, with a marked reduction in males starting at 9 days, and a more pronounced decline in females at later stages (Fig. 6B). Spleen weight only increased at the very late stage (17 weeks on diet) (Fig. 6B), which aligns with the elevated inflammation levels in the liver at this point.

Plasma levels of cholesterol and fasting glucose were higher upon WD from the beginning of the diet challenge (Fig. 6C). Insulin levels, HOMA-IR, ALAT and ASAT levels showed a significant increase only after 17 weeks of WD challenge, indicating that both insulin resistance and liver dysfunction appear at the latest stage (Fig. 6C). Similarly, GDF15, TIMP1 and FGF21 levels at the latest time point (Fig. 6C), with an initial rise occurring after 3 days in males, indicating an acute response. The levels subsequently returned to normal before rising again after prolonged exposure to the diet (Figs. 6C, S8B). We then analyzed the liver histology across all the time points. Liver fibrosis and steatosis became evident upon WD during the late stages of the diet challenge, while the inflammatory marker CD45 showed a progressive increase over time (Figs. 6D, E, S9A and S9C). We observed a stronger correlation between liver fibrosis and the collagen-related genes *Col1a1*, *Col1a2*, and *Col3a1* in females compared to males (Fig. S9C). Activated hepatic stellate cells were evaluated by α-SMA immunofluorescence, showing an increase at 17 weeks (Fig. S9D), consistent with the fibrosis results. We also performed NAS and fibrosis scoring throughout the longitudinal study. Males showed an increase in steatosis, ballooning, total NAS, and fibrosis scores in WD at both 9 and 17 weeks, whereas females exhibited these differences only at 17 weeks of challenge (Fig. 6E).

Given the critical role of hepatic cholesterol in MASH, we measured total liver cholesterol content and found no changes between CD and WD (Fig. S8D**)**. However, recent findings suggest that cholesterol in the lipid droplets serves as a more accurate marker for MASH than total cholesterol and acts as a key mediator of the disease^49^. Therefore, we measured total cholesterol, as well as triglycerides and cholesterol specifically in the liver lipid droplets (Fig. S8C, D**)**. Both triglycerides and cholesterol in the lipid droplets showed an increase in response to WD from day 3 onward and remained elevated until the end of the study (Fig. S8C, D**)**. Taken together, these findings indicate that 17 weeks on a WD diet is sufficient to induce liver damage and fibrosis in both male and female PWK/PhJ mice, and earlier timepoints show a milder phenotype.

**Fig. 7:**
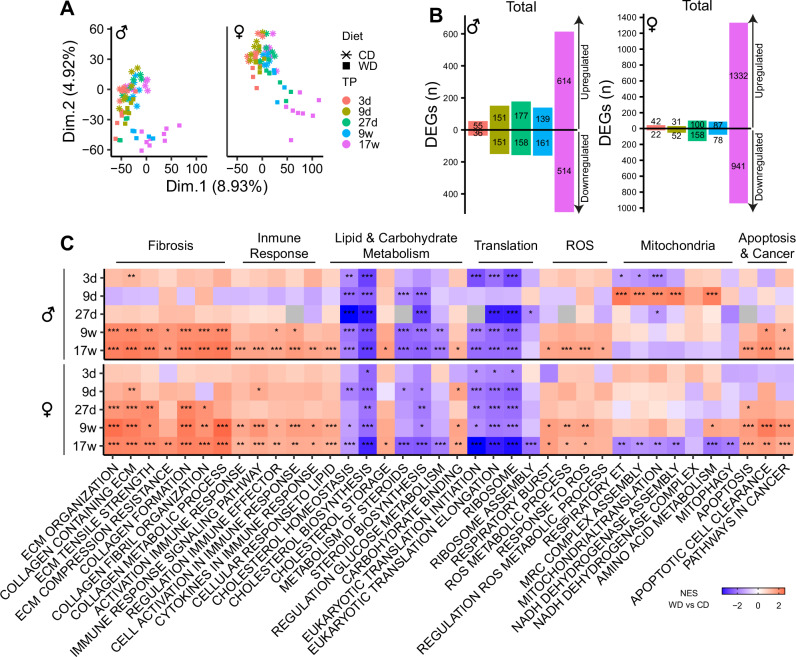
Longitudinal liver transcriptome analysis reveals distinct early and late-stage events. **A** PCA of normalized liver expression data. **B** Total number of liver genes significantly up- or downregulated upon WD compared to CD for males (left) and females (right) in each experimental group. **C** GSEA results for representative gene sets relevant for MASH. 3 d: TN-males, *n* = 6 CD, 6WD; TN-females, *n* = 6 CD, 6 WD; 9 d: TN-males, *n* = 6 CD, 6WD; TN-females, *n* = 6 CD, 6 WD; 27 d: TN-males, *n* = 7 CD, 5 WD; TN-females, *n* = 7 CD, 6 WD; 9w: TN-males, *n* = 7 CD, 5 WD; TN-females, *n* = 6 CD, 5 WD; 17w: TN-males, *n* = 8 CD, 9 WD; TN-females, *n* = 8 CD, 8 WD. For **C**, statistical analysis was conducted using a two-sided Student’s *t*-test with Benjamini–Hochberg adjusted *p*-values. *P < 0.05, **P < 0.01, ***P < 0.001. Source data are provided as a Source Data file.

Next, to assess gene expression changes associated with early and late events, as well as sex-specific differences, we performed BRBseq analysis on the liver and heart tissues throughout the different time points of the longitudinal characterization. In liver, PCA of the gene expression data revealed a separation between CD and WD groups in both sexes at 9 and 17 weeks only (Fig. 7A).

We then analyzed the impact of the WD at each time point on cellular composition in liver using single-cell deconvolution. No differences were found in cellular composition until 17 weeks after WD challenge (Fig. S10A). We also identified the DEGs upon WD, with most of the changes occurring at the final time point. Females had almost twice as many DEGs as males, while the earlier time points showed only a small number of DEGs in both sexes (Fig. 7B). Next, we performed a GSEA to identify the pathways underlying those expression changes. Both males and females showed negatively enriched pathways of lipid and carbohydrate metabolism (Fig. 7C) as an early response maintained over time, in line with the elevated cholesterol and glucose levels in plasma observed under a WD (Fig. 7C). This indicated a shift in metabolism towards stress response pathways triggered by dietary nutrient overload^50^. In response to stress, rapid repression of gene expression enables organisms to shift their cellular machinery to a protective state, prioritizing survival and damage prevention^51^. Notably, translation was negatively enriched from the beginning in the livers under a WD challenge and maintained over time (Fig. 7C). This may have severe consequences on protein synthesis, potentially impairing the function of other tissues even in the absence of changes in gene expression in those tissues. This hypothesis relies on the central role of the liver in producing essential proteins for metabolism, homeostasis, and regulating other organs. GSEA revealed positively enriched pathways related to fibrosis, immune response, and ROS metabolism, observed as part of middle-stage events (27 days diet) in females and late-stage events (from 9 weeks diet) in males (Fig. 7C).

PCA of the transcriptomic data in the heart did not reveal any significant time- or diet-dependent effects in either sex (Fig. S10B). Single-cell deconvolution data also showed no changes in cellular composition at any time point (Fig. S10C), and the number of DEGs was low up to 9 weeks of the diet (Fig. S10D). GSEA revealed minimal changes in pathways at different time points, with no overlap between males and females (Fig. S10E). These findings support the previous conclusion about the minimal WD diet effects on heart transcriptomics, extended over time.

### Chronic WD challenge impacts amino acid and sphingolipid metabolism

To explore metabolic changes in the liver driving the progression of MASLD to MASH, we performed liver targeted metabolomics at the last two time points. These points were selected based on the histological changes, with initial steatosis accumulation becoming evident at 9 weeks and both steatosis, inflammation and fibrosis observed at 17 weeks after WD (Fig. 6D, E).

**Fig. 8:**
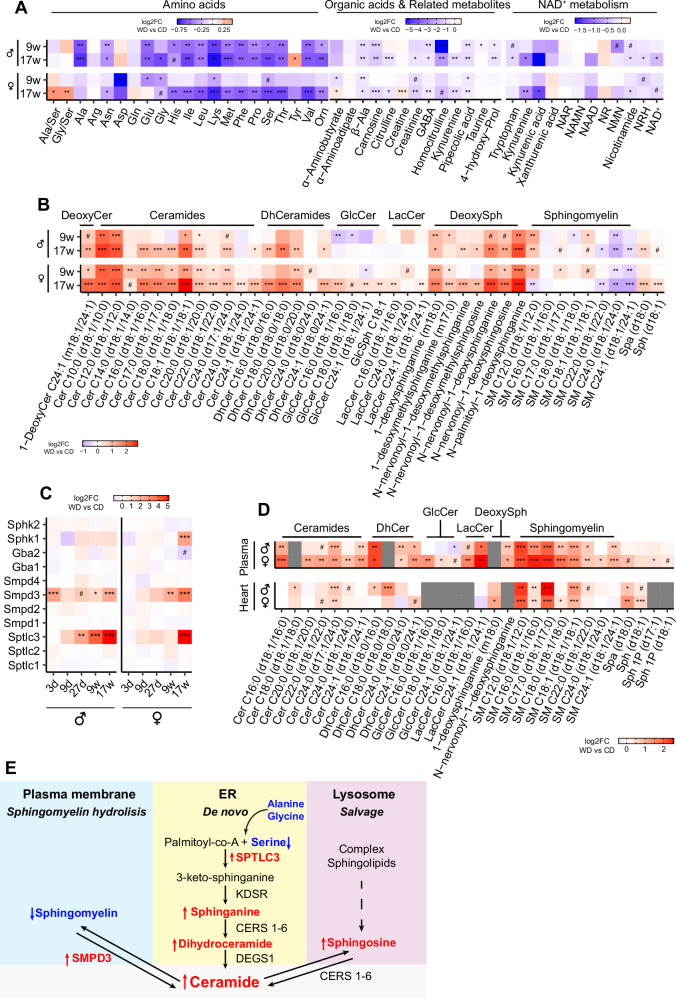
Liver disease in PWK/PhJ model under WD is characterized by disrupted amino acid and sphingolipid metabolism, with sphingolipid release into the bloodstream impacting the heart. **A** Metabolomic WD vs CD log2 fold changes for amino acid, organic acids and related amino acid metabolites and the NAD^+^ pathway in liver tissue. **B** Lipidomic WD vs CD log2 fold changes for sphingolipid species in liver tissue. **C** Liver transcriptomic WD vs CD log2 fold changes for genes associated with sphingolipid metabolic pathways. **D** Lipidomic WD vs CD log2 fold changes for sphingolipid species in plasma and heart tissue. **E** Schematic of sphingolipid pathways, with colors indicating changes in genes and metabolites: red represents upregulation, blue represents downregulation, and black represents no changes upon WD. 9w: TN-males, *n* = 7 CD, 5 WD; TN-females, *n* = 6 CD, 5 WD; 17w: TN-males, *n* = 6 CD, 6 WD; TN-females, *n* = 6 CD, 7 WD. Statistical analysis was conducted using a two-sided Student’s *t*-test with Benjamini–Hochberg adjusted *p*-values. ^#^*P* < 0.1, *P < 0.05, **P < 0.01, ***P < 0.001. Source data are provided as a Source Data file.

Amino acid levels decreased upon WD compared to CD in both males and females at 17 weeks after the diet challenge, with an early decrease in males already at 9 weeks (Fig. 8A). At week 17, a depletion in tryptophan (in males) and kynurenine (in both males and females) metabolites was observed, likely affecting NAD^+^ synthesis as indicated by the lower NAD^+^ levels (Figs. 8A, S11A). Sphingolipids are closely linked to amino acid and lipid metabolism, and elevated levels of ceramides impair the ability of the liver to regulate lipid metabolism, promoting insulin resistance, which is associated with several diseases, including MASH^52–54^.

SPTLC3 is an essential subunit of the SPT enzyme complex, responsible for catalyzing the initial and rate-limiting step in ceramide de novo biosynthesis (Fig. 8E). We observed an increase in *Sptlc3* transcripts in PWK/PhJ mice under WD, with males exhibiting a time-dependent upregulation, while females showed increased transcript levels only at 17 weeks of WD (Fig. 8C). Additionally, we analyzed *Sptlc3* gene expression across different mouse strains and diets using transcriptomic data from our previous MASLD /MASH studies^21,22^. Among all the strains analyzed, PWK/PhJ mice showed the highest increase in *Sptlc3* gene expression under both WD and high-fat diet (HFD) (Figs. 8C, S11C). The decrease in both sexes at 17 weeks in levels of serine and related amino acids (Fig. 8A), essential for de novo synthesis, reflects their utilization in ceramide synthesis, consistent with the observed increase in the levels of ceramide and other sphingolipids (Fig. 8A, B).^13,14^. We analyzed the correlation between the *Sptlc3* gene and the amino acids serine, and the alternative substrate amino acids, alanine, glycine, and phenylalanine, and found that males exhibited a stronger correlation with these alternative amino acids compared to females (Fig. S11B). This may suggest that SPT in males exhibits a wider substrate specificity. In females, the increased intracellular alanine- and glycine-to-serine ratio at week 17 upon WD (Fig. 8A), indicates that the non-canonical de novo synthesis pathway is the main contributor to the increase in deoxy-sphingolipids^13,14^. In males, the absence of changes in those ratios suggests that the increase is more likely attributed to alternative mechanisms^12,55^.

Accumulation of ceramide species may be exacerbated by increased sphingomyelin degradation, as reflected by the decreased levels of sphingomyelin, increased levels of *Smpd3* gene (both males and females), and the increase in sphingosine (Sph) in females (Fig. 8B). Notably, WD but not HFD, induces *Smpd3* levels not only in PWK/PhJ mice but also in other strains (Figs. 8C, S11C). While sphingomyelin hydrolysis can partially contribute to ceramide accumulation, the observed increase in *Sptlc3* transcript levels and enhanced de novo synthesis also play a role in the buildup of ceramide and deoxy-ceramide accumulation in the liver. Ceramides are key mediator of liver fibrosis, promoting hepatocyte apoptosis and inflammation. Thus, the elevated levels of these lipid species in PWK/PhJ mice may underlie their susceptibility to MASH, as well as to the development of liver fibrosis and mitochondrial dysfunction.

The liver is a major source of circulating ceramides, and both ceramides and sphingomyelins (SMs)—particularly Cer(d18:1/16:0) and SM(d18:1/16:0)—have been implicated in the pathogenesis of atherosclerosis^43,56,57^. Hence, we quantified these lipid species in plasma and heart tissue to evaluate their potential association with the cardiac dysfunction phenotype observed in PWK/PhJ mice. As expected, mice under WD showed increased levels of plasma ceramides and SMs (Fig. 8D). Peripheral tissues, including the heart, can incorporate circulating sphingolipids, contributing to tissue lipid accumulation. Accumulation of toxic sphingolipid in the heart has been associated with cardiac lipotoxicity in animal models of obesity^58,59^. To assess potential changes in the PWK/PhJ cardiac lipidome, we hence quantified sphingolipid levels in heart tissue. We observed particularly elevated levels of Cer(d17:1/24:0), SMs and Spa (18:0) in both males and females, while Sph (d18:1) was increased exclusively in females (Fig. 8D). We also measured amino acid levels in plasma and heart tissue and found no significant changes, indicating that de novo ceramide synthesis in the heart is unlikely to contribute to the changes in cardiac sphingolipid levels (Fig. S11D). Together, these results suggest that increased hepatic ceramides contribute to circulating sphingolipids, potentially promoting atherosclerosis and cardiac dysfunction.

## Discussion

In this study, we characterized the longitudinal response of PWK/PhJ strain to WD challenge and TN. Our findings identify that the PWK/PhJ strain not only models the development of MASLD and its transition to MASH but also shows that it develops features of cardiac dysfunction, positioning it as a model for exploring the complex connection between liver and heart dysfunction.

Most metabolic diseases exhibit sexual dimorphism and are influenced by a combination of genetic, hormonal, and environmental factors^23^. Consequently, we first explored the effects of temperature on liver and heart metabolism, while also examining how sex and diet shape these metabolic responses. Our data demonstrated that housing temperature affects body weight, energy expenditure and glucose metabolism in PWK/PhJ mice, consistent with similar studies performed in C57BL/6 mice^60,61^. Female C57BL/6 mice are typically more resistant to diet-induced obesity compared to males^62–64^. A recent study using a diet containing fructose, palmitate, and cholesterol (FPC) found that female mice developed more severe hepatic steatosis, inflammation, and fibrosis. However, male mice did not develop the same fibrotic phenotype, suggesting a sex-specific susceptibility to FPC-induced MASH^65^. In contrast, we found that both male and female PWK/PhJ mice developed liver steatosis, fibrosis and a disease-related transcriptional signature after 17 weeks of challenge with WD. Furthermore, liver steatosis and advanced fibrosis developed in PWK/PhJ mice at 24 weeks of age, compared with 44 weeks of age reported for C57BL/6 mice^66^, indicating that the PWK/PhJ strain is an effective and appropriate model for studying the transition from MASLD to MASH. Our work furthermore revealed that PWK/PhJ mice develop cardiac dysfunction in response to a WD, with females at TN exhibiting a more severe phenotype.

To further characterize the longitudinal effect of WD on MASLD/MASH in PWK/PhJ mice, we evaluated the disease trajectory. We identified early metabolic adaptations to the diet, including increased body weight gain, and elevated plasma glucose and cholesterol levels, while markers of liver dysfunction, such as elevated levels of ALT, AST, TIMP1, FGF21 and GDF15 appeared only at a late stage. We also observed an early and sustained increase in cholesterol content in liver lipid droplets, a key factor in MASH progression and aligning with recent findings^49^. Histopathological analysis (Fig. 6E) showed that the NAS score increased early and progressively, preceding fibrosis, which appeared only at the end of the diet challenge, suggesting that steatosis and inflammation drive subsequent fibrotic progression. Our longitudinal analysis also revealed that mitochondrial dysfunction emerges late in MASH progression, with female mice exhibiting greater susceptibility. This dysfunction perpetuates a harmful vicious cycle of hepatocyte injury, lipid overload, and chronic inflammation, ultimately driving liver fibrosis^25^. Accordingly, 17 weeks of WD feeding are necessary to induce severe liver damage and fibrosis, whereas shorter durations result in milder phenotypes, demonstrating that, the PWK/PhJ model closely recapitulates the pathological progression observed in human disease^3^.

The liver is an important organ for protein synthesis, which depends on an adequate supply of amino acids. Certain amino acids, such as glycine and serine have been observed to decrease in metabolic diseases, while amino acid supplementation has been proposed as a potential treatment^67,68^, which highlights the importance of amino acid balance in liver homeostasis. We found a downregulation of amino acid metabolism in the liver of PWK/PhJ mice following a WD challenge.

Sphingolipids are closely associated with dysregulated intracellular lipid and amino acid metabolism^10,69^. These lipid species, essential for cellular membranes, signaling, and regulation, play a significant role in diseases like atherosclerosis and MASH^17,18^. Ceramides are produced through three distinct pathways (Fig. 8E), with serine being the key precursor in the de novo sphingolipid synthesis pathway. The first step in the de novo pathway is catalyzed by SPT, consisting of three subunits, SPTLC1, SPTLC2 and/or SPTLC3, with the latter being expressed in the liver and linked to increased liver fibrosis, elevated LDL-cholesterol, dyslipidemia, and CVD^70–72^. Under serine deficiency, the first step of the de novo pathway can be carried out by using alternative amino acids^10,12^. Moreover, stable-isotope tracing studies in C57BL/6 mice have shown that glycine-derived serine contributes to sphingolipid synthesis in MASLD^69^. Given the observed low levels of serine and other amino acids in PWK/PhJ mice after a WD challenge, ceramide production may rely not only on de novo synthesis using serine but also on alternative amino acids like alanine or glycine to produce toxic derivatives DeoxySph and DeoxyCer. Both increased de novo ceramide synthesis, particularly via SPTLC3, and enhanced sphingomyelin hydrolysis by SMPD3 contribute to the accumulation of toxic ceramide in liver cells^17,18^. Interestingly, PWK/PhJ males and females show differences in liver sphingolipid metabolism. Both sexes show high ceramides and deoxy-sphingolipids, but in males, low glucosylceramide (GlcCer) may indicate reduced conversion of ceramides to GlcCer, increasing toxicity of accumulating ceramides and deoxy-ceramides. In females, higher GlcCer and lactosylceramide (LacCer) may reduce ceramide toxicity, but inefficient lipid turnover could cause lipid accumulation. The observed increase in ceramides and deoxy-species may contribute to the progression of liver fibrosis in both sexes through lipotoxicity. High ceramide levels and their toxic byproducts can be exported through lipoproteins, primarily in very low-density lipoproteins (VLDL). This export contributes to the elevated plasma ceramide levels, particularly of Cer(d18:1/16:0) and SM(d18:1/16:0), both implicated in atherosclerosis development through their roles in promoting inflammation, plaque instability, and vascular dysfunction^43,56,57^. Additionally, ceramide accumulation in cardiac tissue contributes to heart remodeling and dysfunction, further linking hepatic ceramide dysregulation to CVD progression. Approaches targeting SPT to reduce ceramides have demonstrated effectiveness in treating muscle dystrophies, reducing atherosclerosis and mitigating MASLD phenotype^54,73,74^. Thus, our findings support the idea that amino acids and sphingolipids are not only predictive biomarkers for MASH and CVD but also promising potential drug targets.

In summary, we provide a detailed sex-specific characterization of the PWK/PhJ strain under WD and TN, which replicates multiple aspects of the metabolic syndrome and human MASLD/MASH. Importantly, unlike many other mouse strains, both male and female PWK/PhJ mice are susceptible to developing steatosis, fibrosis, and inflammation and show significant alterations in amino acid and sphingolipid metabolism like seen in the human disease, making them a suitable model to study the transition of MASLD to MASH.

Our data are openly accessible through our online app (https://lisp-lms.shinyapps.io/PWK_study_app/**)** as a valuable resource for the research community, providing free access to explore the phenotyping, metabolic, lipidomic, and transcriptomic traits presented in this work.

### Limitations of the study

Without direct comparison between PWK/PhJ and other strains, particularly in females, it remains unclear whether the cardiac dysfunction phenotype observed is more severe than that in the C57BL/6 strain. Studies across different strains are needed to clarify how strain, sex, and diet modulate the MASH-CVD link. Additionally, although we identified impaired amino acid and sphingolipid metabolism in PWK/PhJ liver tissue as a potential contributor of MASH and cardiac dysfunction following WD challenge, this study does not establish whether these metabolic changes are a cause or a consequence of the observed phenotype. Further studies are needed to validate the molecular mechanisms, including tracing experiments to clarify the link between amino acid reduction and de novo ceramide synthesis in PWK/PhJ mice. We also speculate that SPTLC3 may underlie the greater sensitivity of the PWK/PhJ strain to MASH induction, potentially contributing to mitochondria dysfunction. Understanding SPTLC3 function and the regulation of its expression may provide valuable insight into liver pathophysiology. Hence, liver-specific strategies targeting *Sptlc3* in PWK/PhJ are necessary to establish its role during MASH unequivocally. Finally, using compounds like myriocin and ALT-007^55^ to inhibit de novo ceramide synthesis will be the subject of a more extensive therapeutic study to determine whether the MASH/CVD phenotype in PWK/PhJ mice can be reversed pharmacologically.

## METHODS

### Animals

PWK/PhJ male and female mice were purchased from The Jackson Laboratory (Strain #:003715) and bred at the École Polytechnique Fédérale de Lausanne (EPFL) animal facility. Animals were randomized based on body weight and assigned to different experimental groups. Mice were housed five per cage, with individuals separated when aggression occurred to prevent fighting. Housing conditions included cage enrichment, a 12-h light/dark cycle, *ad libitum* access to food and water, and they were housed at 30 °C (TN) or 22 °C (RT) from 7 weeks of age. WD groups were fed with research diet D12079B (40% kcal from fat, 17% kcal from protein, and 43% kcal from carbohydrates) and the CD groups were fed with research diet D16042904B (10% kcal from fat, 17% kcal from protein, and 73% kcal from carbohydrates). Body weight was measured weekly from 7 weeks of age until sacrifice. In vivo, phenotyping tests started at 18 weeks of age, with a two-week resting interval between each subsequent test, following the pipeline illustrated in Fig. 1A. All animal experiments were performed according to Swiss ethical guidelines and approved by the Service de la Consommation et des Affaires Vétérinaires of the Canton de Vaud (license VD3598).

### Oral glucose tolerance test (OGTT)

At 18 weeks of age, mice were fasted overnight. On the morning of the OGTT experiment, a 20% glucose solution in water (10 mL [2 g]/kg body weight) was administered via gavage to induce hyperglycemia. Blood glucose levels were measured from the tail vein using a glucometer before the gavage and at 15, 30, 45, 60, 90, 120, 150, and 180 min afterwards.

### Body composition analysis (Echo-MRI) and indirect calorimetry

At 20 weeks of age, body composition was assessed using an Echo-MRI machine (the 3-in-1, Echo Medical Systems). This method provided measurements of each mouse’s lean mass, fat mass, and total body weight, with a processing time of approximately one minute per subject. On the same day, the mice were placed in the metabolic and behavioral phenotyping system (Promethion, Sable Systems) for 48 h. They were housed individually in metabolic cages, where movement, oxygen consumption (VO₂), and carbon dioxide production (VCO₂) were recorded every 3 min. The first 24 h were considered for adaptation, while data from the following 24 h were used for analysis.

### Echocardiography

At 22 weeks of age, transthoracic echocardiography was performed on PWK/PhJ mice to evaluate cardiac function and morphology. Mice were anesthetized with 0.8–1.5% isoflurane mixed with O₂, ensuring a light sedation level was maintained throughout the procedure. To prevent ocular dryness during anesthesia, one drop of artificial tears (Viscotears) was applied to each eye. Mice were positioned ventral side up on a heating platform to maintain their body temperature at 37 °C ± 0.5 °C. Heart rate (HR), respiration rate (RR), and body temperature were continuously monitored using ECG electrodes and a rectal probe. The chest area was shaved, and warm ultrasound gel was applied to the area of interest. Transthoracic echocardiography was performed using a Vevo 2100 system (FUJIFILM VisualSonics, Toronto, Canada) with a 40-MHz transducer. Precautions were taken to minimize pressure on the sternum, as excessive force could compromise signal accuracy and quality.

Systolic function was assessed in both B-mode parasternal long axis and M-Mode parasternal short axis view. To ensure proper alignment and accurate measurements, the apex and aorta were aligned, the pulmonary artery was excluded from the long-axis view, and both papillary muscles were included in the short-axis view. Measurements on these 2 views were performed automatically by the AI-based “Auto LV analysis” function from Vevolab 5.7 software (FUJIFILM VisualSonics, Toronto, Canada). Diastolic function was assessed by pulsed wave (PW) Doppler-mode of the mitral flow in an apical 4-chamber view. Aortic flow was assessed by PW Doppler-mode in the descending aorta in an aortic arch view.

All measurements were performed with the Vevolab 5.7 software.

### Plasma analyses

For plasma analysis, blood was collected in ethylenediamine tetraacetic acid (EDTA) coated tubes (Microvette 500 EDTA K3E, Sarstedt). Plasma was isolated by centrifugation at +4 °C and subsequently stored at −80 °C for further analysis. Plasma parameters were measured on 2 times diluted samples (1:1 ratio of plasma to diluent) using Dimension EXL 200 (Siemens Healthcare Diagnostics AG, Dudingen, Switzerland). The biochemical tests were performed according to the manufacturer kit for each parameter: Alkaline phosphatase (Siemens Healthcare, DF15A), enzymatic creatinine (Siemens Healthcare, DF270B), transaminase ASAT (Siemens Healthcare, DF41A), transaminase ALT (Siemens Healthcare, DF143), glucose (Siemens Healthcare, DF40), cholesterol (Siemens Healthcare, DF27), HDL (Siemens Healthcare, DF48B), LDL (Siemens Healthcare, DF131), triglycerides (Siemens Healthcare, DF69A), free fatty acids (FUJIFILM Wako Diagnostics, NEFA-HR (2)), urea nitrogen (Siemens Healthcare, DF21).

Plasma insulin levels were measured using a mouse insulin ELIZA kit (10-1247-01, Mercodia) following the manufacturer’s instructions.

Plasma levels of TIMP1, FGF21, GDF15, KC (CXCL1), TNF-α, MIP-2 (CXCL2), IL-6, MCP-1 (CCL2), IL-1β (IL-1F2), IFN-γ, ICAM-1 (CD54), CXCL10 (IP-10/CRG-2), G-CSF and MMP9 were measured using the Mouse Premixed Multi-Analyte Kit (LXSAMSM, R&D Systems) in a Luminex 200 system following the manufacturer’s instructions. Plasma analyses for individual animals are available in Supplementary Data 1.

### Liver histology

Liver samples were collected from all mice immediately after sacrifice and fixed in 4% buffered formalin for 48 h at 4 °C before being embedded in paraffin. The paraffin-embedded samples were then sectioned into 4 µm thick slices and stained using standard H&E staining to evaluate general morphology, Sirius red (SR) to assess collagen fiber content, and CD45 to identify positive immune cells. Hepatic stellate cells were visualized by immunofluorescence on 8 μm cryosections using α-smooth muscle actin (α-SMA) conjugated to Cy3 (C6198, Sigma-Aldrich). Images were acquired with an Olympus Slide Scanner VS120 L100 microscope at 20x magnification and analyzed using QuPath software.

### Histopathologic analysis

A semiquantitative analysis was performed in a blinded manner by a board-certified veterinary pathologist as described by Kleiner et al.^75^. NAS components such as steatosis, lobular inflammation and hepatocyte ballooning were assessed on H&E-stained sections. In summary, scores were given as follows: (A) steatosis (100x High Power Field (HPF)): grade 0 = <5%, 1 = 5–33%, 2 = 33–66%, 3 = >66%; (B) lobular inflammation (200x HPF): 0 = none, 1 = <2 foci, 2 = 2–4 foci, 3 = >4 foci; (C) hepatocyte ballooning (whole tissue section assessed): 0 = none, 1 = few balloon cells, 2 = prominent ballooning. The total NAFLD score was calculated as the sum of the single scores A–C and ranged from 0 to 8. Details of the scoring system, the calculation and statistical analysis, as well as the individual animal NAS scores are available in Supplementary Data 2.

Fibrosis scoring was performed separately on SR-stained sections with the following scores given (whole tissue section assessed): 0 = none, 1 = perisinusoidal or periportal, 2 = perisinusoidal and portal/periportal, 3 = bridging, 4 = cirrhosis. Details of the scoring system, the calculation and statistical analysis, as well as the individual animal fibrosis scores are available in Supplementary Data 2.

### Preparation of liver tissue extracts

For protein extraction, 20 mg frozen liver tissue was homogenized in ice-cold RIPA buffer (50 mM Tris-HCl pH 8, 150 mM NaCl, 0.5% deoxycholate, 0.1% SDS, 1% Triton X-100) supplemented with 1 mM DTT, 1 mM PMSF, 10 μg/mL each of chymostatin, leupeptin, antipain, and pepstatin A (CLAP), and phosphatase inhibitor cocktails 2 and 3 (Sigma-Aldrich, P5726 and P0044) diluted at 1:100. Samples were homogenized using a Mixer Mill MM 300 (Retsch, Germany) at 25 Hz for 1 min. Homogenates were centrifuged at 10000 *g* for 15 min at 4 °C to eliminate cell debris. The total amount of protein was estimated by Bradford assay (Bio-Rad, 5000001).

### Electrophoresis and Western blot immunodetection

A total of 50 μg of protein was mixed with SDS–dithiothreitol loading buffer (10% sucrose, 2 mM EDTA, 1.5% SDS, 20 mM dithiothreitol, 0.01% bromophenol blue and 60 mM Tris–HCl, pH 6.8) and denatured by heating at 100 °C for 5 min. Proteins were separated by SDS–PAGE on 4–20% polyacrylamide gradient gels and transferred onto nitrocellulose membranes using the Trans-Blot Turbo Transfer System (Bio-Rad, USA). Membranes were blocked with 5% (w/v) powdered milk for 1 h at RT and were then incubated overnight at 4 °C with primary antibodies and diluted at 1:1000: anti-COL1A2 (Santa Cruz, sc-393573), anti α-SMA (Invitrogen, 14-9760-82), anti-TIMP1 (Abcam, ab179580) and anti-CTGF (Abcam, ab318148). Binding sites were detected using species-specific HRP-conjugated secondary antibodies diluted at 1:10000 and incubated for 1 h at RT: goat-anti-rabbit (Azure Biosystems, AC2114) and goat-anti-mouse (Azure Biosystems, AC2115). Following incubation with secondary antibodies, protein bands were visualized by chemiluminescence (ECL-Plus, GE Healthcare Life Sciences) and the signal was recorded using a ChemiDoc Imaging System (Bio-Rad, USA). Band intensities were quantified with Image Lab 6.1 software (Bio-Rad, USA) and normalized to Ponceau S staining.

### Liver lipid droplet fractionation and metabolite quantification

Lipid droplet isolation was performed as previously described^49^. Briefly, 40–50 mg of powdered liver tissue was homogenized in 700 μL of buffer A (20 mM Tris-HCl, pH 7.4, 1 mM EDTA, 0.25 mM EGTA, 250 mM sucrose). One third of the homogenate was reserved for liver total cholesterol measurement, and the remainder was transferred to ultracentrifuge tubes overlaid with 300 μL of 3% sucrose and centrifuged at 100000 *g* for 1 h at 4 °C. The lipid layer was collected in full using a needle to obtain the lipid droplet fraction.

Lipids from homogenates and lipid droplet fractions were extracted with 3 ml chloroform/methanol (2:1, v/v) followed by 1 ml dH_2_O. After vortexing and centrifugation (1800 *g*, 10 min, room temperature), the methanol phase was collected for downstream analyses.

For cholesterol quantification, 1 mL of the methanol phase was dried under nitrogen, reconstituted in 200 μL of 1 × assay diluent containing 0.25% NP-40 (Total Cholesterol Assay Kit, Cell Biolabs), and analyzed according to the manufacturer’s instructions. For triglyceride quantification, 22.5 μL of the methanol phase was dried under nitrogen and analyzed using the Triglyceride-SL Reagent Assay (Sekisui Diagnostics, USA).

### RNA preparation for BRB-seq analysis

For mRNA extraction, 10–20 mg of powdered tissue was homogenized in 1 mL of Trizol with ceramic beads using the Precellys Evolution Touch Homogenizer, coupled with the Cryolys Evolution cooling system (8800 rpm, 5 s, 4 °C). Samples were centrifuged at 12000 *g* for 10 min at 4 °C, and the supernatant was mixed with chloroform at a ratio of 200 µL per 1 mL of Trizol. Afterward, the mixture was centrifuged again at 12000 *g* for 15 min at RT, and the upper phase was combined with an equal volume of 100% ethanol. Next steps were performed using the direct-zol-96 RNA kit (R2054, Zymo Research). mRNA concentration was measured for all samples. All samples passed a quality check of purity (NanoDrop) and fragmentation (FragmentAnalyzer).

### Library preparation for BRB-seq analysis

Bulk RNA barcoding and sequencing (BRB-seq) libraries were generated and sequenced by Alithea Genomics (Switzerland)^76^.

RNA sequencing libraries were prepared using the Mercurius™ BRB-seq Library Preparation Kit for 384 samples (Alithea Genomics, PN 10814). For each sample, 50–1000 ng of total purified RNA was used as input. Individual samples were first reverse-transcribed using barcoded Mercurius™ oligo-dT primers to tag each sample with a unique 14-nt sample barcode and a 14-nt Unique Molecular Identifier (UMI) during first-strand synthesis. Following reverse transcription, the barcoded cDNA samples were pooled into a single tube for streamlined processing.

Non-incorporated primers were removed from the pool via free primer digestion using EXB and EXO reagents. Double-stranded full-length cDNA was then generated through second-strand synthesis and purified using SPRI AMPure Beads (Beckman Colter, PN A63881).

The resulting cDNA was tagmented using a pre-loaded Tn5 transposase to fragments optimized for library amplification. The 5’ terminal fragments were subsequently amplified and indexed using Unique Dual Indexing (UDI) adapter primers provided in the kit (PN 10524). The final library was subjected to quality control via fragment analysis (aiming for a range of 300–1000 bp) and quantified using Qubit (Thermo Fisher Scientific).

### Sequencing and data processing

The libraries were sequenced on an Illumina instrument (or Element Biosciences AVITI system). Raw FASTQ files (Read 1 and Read 2) were generated after standard demultiplexing of the UDI adapters. Read 1 contained the sample barcode and UMI, while Read 2 contained the gene fragment.

Bioinformatics analysis was performed by aligning the sequencing reads to the mouse reference genome (GRCm39/mm39) using the STAR aligner (version 2.7.9a) in STARsolo mode. This step allowed for the simultaneous alignment of reads and the generation of gene and UMI read count matrices. Counts per million (CPM) normalization and downstream visualizations were performed using R (version 4.1.a) with the data.table and Matrix packages^77^. For further analyses, R (version 4.1.0) through RStudio was used.

Differential expression analysis was conducted using limma-voom (version 3.50.3)^78^, using as contrast WD vs. CD. GSEA was performed using the clusterProfiler R package (version 4.2.2)^79^. The gene sets (Reactome, GO, Hallmarks, and KEGG) were retrieved from the msigdbr R package (version 7.5.1)^80^. To perform single-cell deconvolution analysis, MuSiC R package (version 1.0.0)^81^ was used. The GEO dataset GSE109774 was used as the reference single-cell RNA-seq dataset^41^. Benjamini–Hochberg adjusted p-values below 0.05 were considered the significance threshold in all statistical analyses. Bar plots, heatmaps, boxplots, and scatterplots were created using ggplot2 (version 3.5.1), as well as Venn diagrams, through the ggplot2-dependent package ggvenn (version 0.1.10). Upset plots were generated using UpSetR^82^ (version 1.4.0). Heart samples HDP-018715 and HDP-019057 were excluded from downstream analysis due to suspected sample swap.

### Human and mouse MASLD/MASH RNA-seq datasets

Processed counts from public human liver bulk RNA-seq data of MASLD/MASH patients were obtained from Gene Expression Omnibus (GEO) under the accession numbers GSE135251 and GSE130970^47,48^. DEGs were determined using the contrast NAS ≥ 4 vs NAS < 4. Processed counts from public mouse liver bulk RNA-seq data of CC founder strains were obtained from Gene Expression Omnibus (GEO) under the accession numbers GSE201819 and GSE182668^21,22^. DEGs were determined using the contrast WD vs. CD. Functional analyses, including Gene Ontology (GO) term enrichment and GSEA and ORA, were performed using the clusterProfiler R package.

### Proteomics sample preparation

300 µl of guanidine hydrochloride solution in Tris-buffered saline at pH 8 was added to 20 mg liver frozen powder. Samples were homogenized using Precellys (Bertin Technologies) for 2 min at 6500 rpm and 4 °C, followed by centrifugation for 10 min at 21000 *g* and 4 °C. The supernatants were heated up to 65 °C for 5 min, and bath sonicated at medium strength for another 5 min. The samples were subsequently centrifuged at 1800 *g* for 10 min at 4 °C, and the supernatant was recovered. Protein concentrations were determined with a Bradford assay, and 30 µg of protein was used for the proteomics sample preparation procedure. The samples were then reduced, alkylated, and trypsinized. After trypsinization, peptides were purified using SOLAµ HRP SPE plates and eluted in an 80% acetonitrile solution with 0.1% trifluoroacetic acid in water. Peptides were then dried and analyzed by liquid chromatography with tandem mass spectrometry (LC-MS/MS).

### Proteomics library creation

A library was first created by mixing equal amounts of proteins from each sample. Digested and desalted samples were fractionated into 12 fractions by High pH Reverse Phase Chromatography using an Integrated Vanquish Fraction Collector system (ThermoFischer Scientific). Briefly, 100 µg of the mixed sample was injected on a Waters Acquity UPLC BEH C18 (1.0 mm x 100 mm) column and separated through a 90 min. gradient using 10 mM Bicarbonate Ammonium in 5% Acetonitrile as Solvent A and 10 mM Bicarbonate Ammonium in 90% Acetonitrile as Solvent B. Fraction collection was performed every minute with a concatenation-pooling strategy resulting in 12 final fractions directly dried by vacuum concentrator. Fractions were finally resuspended in 2% acetonitrile; 0.1% formic acid and 1 µg was injected for LC-MS/MS analysis over 90 min. gradients using standard shotgun DDA acquisition mode.

### Proteomics BoxCar LC-MS/MS

Individual samples were acquired using a BoxCar LC-MS/MS method described elsewhere^83^. Briefly, due to protein high dynamic range of the samples, BoxCar termed acquisitions were performed through sequential and interspaced narrow m/z windows, ultimately covering the full mass range. A first full scan was acquired, followed by two BoxCar-based ones covering 400 to 1’200 m/z range. The nano-flow separations were performed on a Vanquish Neo nano UPLC system (ThermoFisher Scientific) connected on-line with an Exploris 480 Orbitrap mass spectrometer (ThermoFisher Scientific) at the EPFL Proteomics Core Facility. A capillary precolumn (Acclaim PepMap C18; 3 μm-100 Å; 2 cm × 75 μm ID; ThermoFisher Scientific) was used for sample trapping and cleaning. Analytical separations were performed at 250nL/min over a 90 min. biphasic gradient on a 50 cm long in-house packed capillary column (75 μm ID; ReproSil-Pur C18-AQ; 1.9 μm silica beads; Dr. Maisch, Ammerbuch). Initial full scans were acquired with a resolution of 120’000 (i.e., at 200 m/z) as well as the two following BoxCar scans. The 5 most intense parent ions were selected from the first full scan and fragmented by High-Energy Collision Dissociation (HCD) with a Normalized Collision Energy (NCE) of 30%, using an isolation window of 1.4 m/z. Fragmented ion scans were acquired with a resolution of 15’000 (i.e., at 200 m/z) and selected ions were then excluded for the following 25 s.

### Proteomics data analysis

We compiled an *in-silico M. musculus* proteome from the PWK/PhJ strain in Ensembl release 113. The resulting FASTA files were then preprocessed with an in-house script to remove all initiator methionines and signal peptides, as annotated in Uniprot. Thermo raw files were searched with MaxQuant version 2.4.4.0. Cysteine carbamidomethylation was included as a fixed modification, while methionine oxidation and protein N-terminal acetylation were included as variable modifications. We allowed for a maximum of two missed cleavages and searched the data with MaxQuant’s match-between runs feature, while also enabling the identification of second peptides.

Protein intensities were imported from MaxQuant’s proteinGroups.txt file into R, using the proteoDA package (version 1.0.1). The analysis followed the protocol described by Pan ZF et al.^84^. Proteins identified only by site, potential contaminants, reverse hits, or with fewer than two unique peptides were removed. Proteins with more than 90% missing values in any group were also filtered out. The resulting LFQ intensity matrix was normalized using variance-stabilizing normalization (vsn) (version 3.76.0). Principal component analysis (PCA) was performed on log2-transformed intensities with missing values imputed using iterative PCA (missMDA, version 1.20) to assess global variation among samples and detect potential outliers. Based on PCA, liver sample S_42 (sample ID HDP-018879, corresponding to a male CD-RT) was excluded from the downstream analysis due to suspected mislabeling with sample ID HDP-018897, corresponding to a female CD-RT. Differential protein abundance was analyzed using limma, and *p*-values were adjusted for multiple testing using the Benjamini–Hochberg procedure. A model design matrix without intercept (~0 + group) was constructed, where group encodes sex, diet, and temperature. Similar to BRB-seq data, GSEA was performed on proteomics results using clusterProfiler R package. Data visualizations, including bar plots, heatmaps and scatterplots, were generated using ggplot2. Venn diagrams were created using the ggvenn package.

### Targeted LC-MS/MS analysis of Sphingolipids

For absolute quantification of sphingolipids, 30 mg of powdered tissue and 25 µL plasma samples were homogenized with water. Subsequently, 25 µL of the resulting homogenate was extracted using 200 μL of ice-cold methanol (MeOH) containing internal standards. Sample extracts were vortexed and centrifuged (4500 *g*, 15 min, 4 °C). The supernatant was transferred to a 96-well plate and injected into the LC-MS/MS system. Ten-point calibration curves were prepared using the same procedure.

Samples and calibrators were analyzed by Liquid Chromatography coupled to tandem mass spectrometry (LC - MS/MS) in positive ionization mode using a TSQ Altis triple quadrupole instrument (QqQ) interfaced with a Vanquish UHPLC system (Thermo Fisher Scientifics). Chromatographic separation was carried out in a Zorbax Eclipse plus C18 column (1.8 μm, 100 mm × 2.1 mm I.D., Agilent Technologies). The mobile phase consisted of A (5 mM ammonium formate, 2% formic acid in water) and B (5 mM ammonium formate, 0.2% formic acid in MeOH) with a flow rate of 600 μL/min. The column temperature was 40 °C and the sample injection volume 4 µL. The linear gradient elution starting from 80% to 100% of B (in 8 min) was applied and held until 14 min. The column was then equilibrated to initial conditions. Optimized HESI source parameters were set as follows: voltage 3500 V in positive mode, Sheath Gas (Arb) = 60, Aux Gas (Arb) = 15, Sweep Gas (Arb) = 1 and Ion Transfer Tube Temperature 380 °C. Nitrogen was used as the nebulizer and Argon as collision gas (1.5 mTor). Vaporizer Temperature was set to 350 °C. Optimized compound-dependent parameters were used for data acquisition in timed- Selected Reaction Monitoring (t-SRM) mode.

Raw LC-MS/MS data acquired in t-SRM mode were processed with the clinical trace finder software (version 4.1, Thermo Fisher Scientific). Quantification was based on extracted ion chromatogram areas for the monitored t-SRM transitions, corresponding calibration curves, and the corresponding (or structural analogs) stable isotope-labeled internal standard response. The linearity of the standard curves was evaluated for each metabolite using ten calibration points, and peak area integrations were manually curated and corrected when necessary. The concentrations were normalized to total protein content (determined using the BCA assay). Supporting Information is available in Supplementary Data 3. Analyses of individual animal sphingolipid metabolites are available in Supplementary Data 4.

### Targeted LC-MS/MS analysis of amino acids

For amino acid quantification, 30 mg of powdered tissue and 25 µL plasma samples were homogenized with water. Subsequently, 20 µL of the resulting homogenate was extracted with a pre-cooled MeOH:H_2_O (5:1, v/v) solvent mixture containing isotope-labeled internal standard. Sample extracts were vortexed and centrifuged (2700 *g,* 15 min, 4 °C). The resulting supernatant was collected and injected into the LC-MS system. Ten-point calibration curves were prepared using the same procedure^85^.

Extracted samples were analyzed by LC-MS/MS in positive ionization mode using a Triple Quadrupole mass spectrometer (6495 iFunnel Agilent). The chromatographic separation was carried out in an Acquity BEH Amide, 1.7 μm, 100 mm × 2.1 mm I.D. column (Waters, Massachusetts, US). The mobile phase consisted of A (20 mM ammonium formate, 0.1% formic acid in water) and B (0.1% formic acid in acetonitrile). The linear gradient elution from 95% B (0–1.5 min) down to 45% B was applied (1.5 min–17 min), and these conditions were held for 2 min. The initial chromatographic condition was maintained as a post-run for 5 min for column re-equilibration. The flow rate was 400 μL/min, the column temperature was 25 °C, and the volume of the sample was 2 µl. ESI source conditions were set as follows: dry gas temperature 290 °C, nebulizer 35 psi and flow 14 L/min, sheath gas temperature 350 °C and flow 12 L/min, nozzle voltage 0 V, and capillary voltage 2000 V. Dynamic Multiple Reaction Monitoring (DMRM) was used as acquisition mode with a total cycle time of 600 ms. Optimized collision energies for each metabolite were applied.

Raw LC-MS/MS data were processed using the Agilent Quantitative analysis software (version B.07.00, MassHunter Agilent Technologies). Peak areas were based on EIC (Extracted Ion Chromatogram) areas for the monitored MRM transitions and translated into concentrations using calibration curves and ISTD spike (i.e., response factor). The concentrations were normalized to total protein content (determined using the BCA assay). Supporting Information is available in Supplementary Data 3. Analyses of individual animal amino acid metabolites are available in Supplementary Data 5.

### Targeted LC-MS/MS analyses of NAD+ metabolome

For NAD^+^ metabolites quantification, 30 mg of powdered tissue and 25 µL plasma samples were extracted with MeOH:H_2_O (4:1, v/v). Subsequently, 100 µL of the resulting homogenate were spiked with 25 µL of internal standard solution and then the sample extracts were evaporated to dryness using a centrivap centrifugal vacuum concentrator (Labconco, Kansas City, Missouri, US). The resulting residues were reconstituted in 75 µL of water and injected into the LC–MS system. Ten-point calibration curves were prepared using the same procedure^86^.

Extracted samples were analyzed by LC–MS/MS using ultra-high performance liquid chromatography (UHPLC) on an Agilent 1290 Infinity system (Agilent Technologies), coupled to a triple quadrupole mass spectrometer (Agilent 6495 iFunnel) equipped with an Agilent Jet Stream ESI source for quantification. The chromatographic separation was carried out in a Scherzo SM-C18 column. The mobile phase consisted of A (20 mM ammonium formate and 0.1% formic acid in H_2_O), and B (20 mM ammonium formate and 0.1% formic acid (90:10, v/v) in acetonitrile). The gradient started at 100% A (0–2 min), increased to 100% B by 12 min and maintained at 100% B for 3 min. Then it was returned to 100% A in 1 min, followed by an isocratic hold at the initial conditions from 16 to 22 min to allow column re-equilibration. The flow rate was 200 µL/min, the column temperature was 30 °C, and the volume of the sample was 2 µL.

ESI source conditions were set as follows: dry gas temperature 290 °C, nebulizer 45 psi and flow 12 L/min, sheath gas temperature 350 °C and flow 12 L/min, nozzle voltage 500 V, and capillary voltage 4000 V. Dynamic Multiple Reaction Monitoring (DMRM) was used as the acquisition mode with a total cycle time of 600 ms. Optimized collision energies for each metabolite were applied.

Raw LC-MS/MS data were processed using the Agilent Quantitative analysis software (version B.07.00, MassHunter, Agilent Technologies). Peak areas were based on EIC areas for the monitored MRM transitions and translated into concentrations using calibration curves and ISTD spike (*i.e*., response factor). The concentrations were normalized to total protein content (determined using the BCA assay). Supporting Information is available in Supplementary Data 3. Individual animal NAD⁺ metabolome analyses are available are available in Supplementary Data 6.

### Reporting summary

Further information on research design is available in the Nature Portfolio Reporting Summary linked to this article.

## Supplementary information

Supplementary Information

Description of Additional Supplementary Information

Supplementary Data 1

Supplementary Data 2

Supplementary Data 3

Supplementary Data 4

Supplementary Data 5

Supplementary Data 6

Reporting Summary

Transparent Peer Review file

## Source data

Source Data

## Data Availability

Source Data are provided with this paper. Our data are openly accessible through our online app (https://lisp-lms.shinyapps.io/PWK_study_app/**)** as a valuable resource for the research community, providing free access to explore the phenotyping, metabolic, lipidomic, and transcriptomic traits presented in this work. BRB-seq data used in this study were deposited in the Gene Expression Omnibus (GEO) database (GSE309251; https://www.ncbi.nlm.nih.gov/geo/query/acc.cgi?acc=GSE309251), and proteomics raw data were deposited at PRoteomics IDEntifications (PRIDE) (Liver: PXD068560; https://www.ebi.ac.uk/pride/archive/projects/PXD068560, Heart: PXD068595; https://www.ebi.ac.uk/pride/archive/projects/PXD068595). RNA-seq data of MASLD/MASH patients were obtained from Gene Expression Omnibus (GEO) under the accession numbers GSE135251 and GSE130970^47,48^. RNA-seq data of CC founder strains were obtained from Gene Expression Omnibus (GEO) under the accession numbers GSE201819 and GSE182668^21,22^. Mouse tissue samples generated in this study are available under a collaboration agreement with the lead contact, Johan Auwerx (admin.auwerx@epfl.ch). Source data are provided with this paper.

## Code availability

This study did not generate original code and instead used standard R libraries described in the methods. R Scripts required to reanalyze the data reported in this paper are available from the corresponding authors upon request.
