# Supplementary material for: Western diet-induced MASH in PWK/PhJ mice identifies disruptions in amino acid and sphingolipid metabolism contributing to cardiac dysfunction
Source: Nat Commun. 2026 May 20;17:6629. doi: 10.1038/s41467-026-73449-7 (PMC13381867; doi:10.1038/s41467-026-73449-7)

Western diet-induced MASH in PWK/PhJ mice identifies disruptions in amino acid and sphingolipid metabolism contributing to cardiac dysfunction

Sandra Rodríguez-López[1*], Miguel Pérez-Rodríguez[2], Alaa Badreddine[1], Rafael Calais Gaspar[3,4], Henrique J. Novaes Morgan[3,4,5], Ikki Sakuma[3,4], Giacomo V G von Alvensleben[1], Alejandro Alonso-Calleja[6], Stacia P.A Everts[1], Nicolas-Enzo Suter[1], Giorgia Benegiamo[1], Christine Goepfert[7,8], Simone de Brot[7], José Manuel Villalba[2], Gerald I Shulman[3,4], Kristina Schoonjans[6], and Johan Auwerx[1*]

[1]Laboratory of Integrative Systems Physiology, École polytechnique fédérale de Lausanne, Lausanne, Switzerland.

[2]Departamento de Biología Celular, Fisiología e Inmunología, Universidad de Córdoba, Campus de Excelencia Internacional Agroalimentario CeiA3, Córdoba, Spain.

[3]Department of Internal Medicine, Yale University School of Medicine, New Haven, CT, USA.

[4]Department of Cellular & Molecular Physiology, Yale University School of Medicine, New Haven, CT, USA.

[5]Department of Physiology, Ribeirão Preto Medical School, University of São Paulo, Riberão Preto, Brazil.

[6]Laboratory of Metabolic Signaling, Institute of Bioengineering, Ecole Polytechnique Fédérale de Lausanne, Lausanne, Switzerland.

[7]COMPATH, Institute of Animal Pathology, University of Bern, Bern, Switzerland.

[8]Histology Core Facility, Ecole Polytechnique Fédérale de Lausanne, Lausanne, Switzerland.

*Correspondence

J.A: admin.auwerx@epfl.ch and S.R.L: b02rolos@gmail.com

Supplementary figures

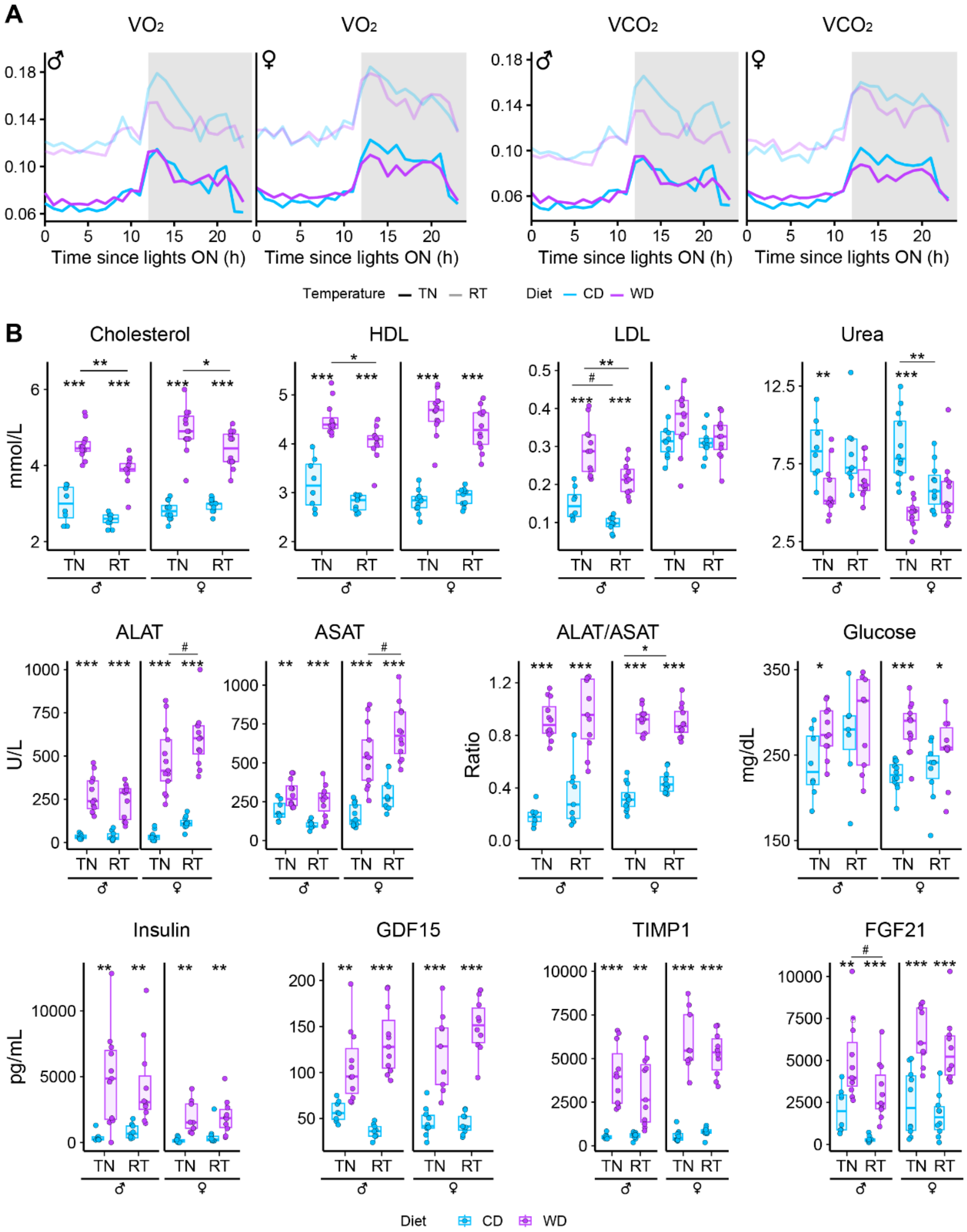

Supplementary Figure 1. Sex-specific effects of housing temperature on metabolism and plasma analytes in PWK/PhJ mice. (A) Oxygen and carbon dioxide consumption and production rates. The white and shaded areas represent light and dark phases (12h each) respectively. TN-males, n= 8 CD, 10 WD; TN-females, n= 10 CD, 10 WD; RT-males, n= 9 CD, 11 WD; RT-females, n= 11 CD, 12 WD. **(B)** Plasma analyte concentrations measured at the end of the study (week 24) and after 4 hours of fasting. For GDF15, TIMP1 and FGF21: TN-males, n= 8 CD, 11 WD; TN-females, n= 10 CD, 9 WD; RT-males, n= 9 CD, 11 WD; RT-females, n= 10 CD, 10 WD. For ICAM1 and MMP9: TN-males, n= 6 CD, 6 WD; TN-females, n= 6 CD, 6 WD; RT-males, n= 6 CD, 6 WD; RT-females, n= 6 CD, 5 WD. For the remaining plasma markers:

TN-males, n= 8 CD, 12 WD; TN-females, n= 12 CD, 13 WD; RT-males, n= 9 CD, 11 WD; RT-females, n= 11 CD, 12 WD. For B, results are shown as box-and-whisker plots. The lower and upper hinges correspond to the first quartile (25th percentile) and third quartile (75th percentile), with the median represented by a line in the center. The whiskers show the minimum and maximum values in the data. Points beyond the whiskers are outliers, plotted individually. Statistical analysis was performed using a two-way ANOVA followed by Tukey's post hoc test. $^{\#}P < 0.1$, $^{*}P < 0.05$, $^{**}P < 0.01$, $^{***}P < 0.001$.

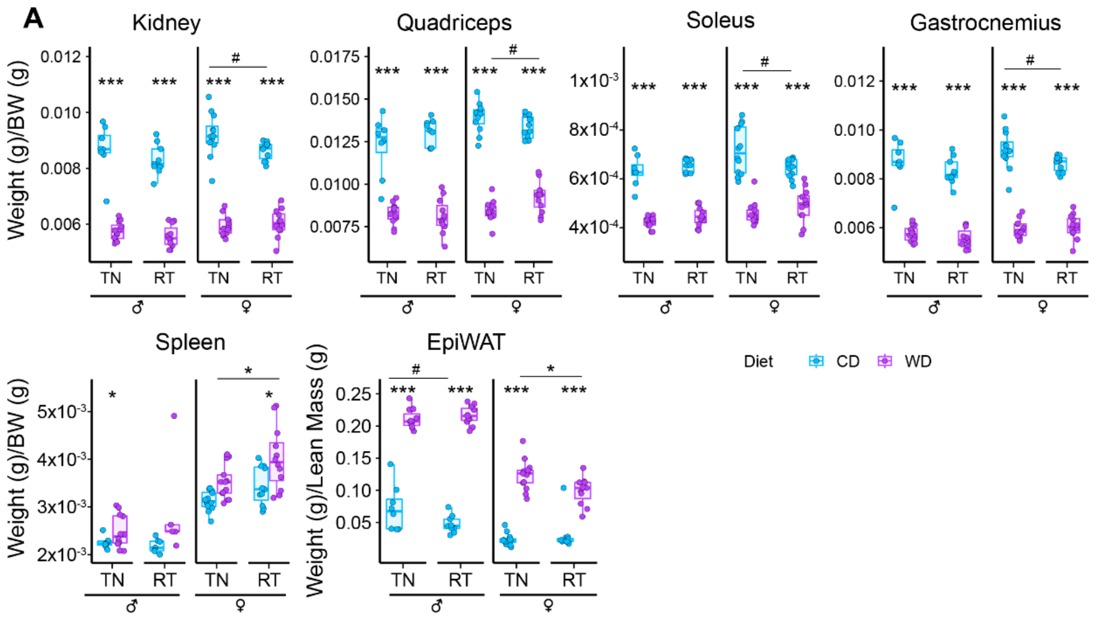

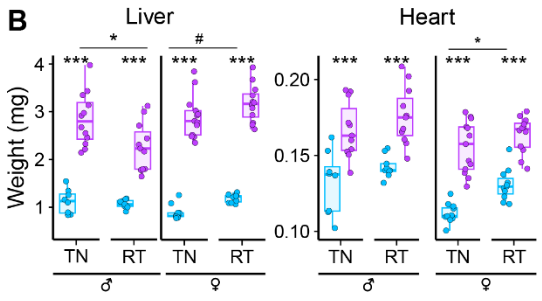

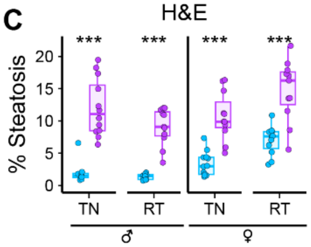

D

	TN				RT			
	Male		Female		Male		Female	
	CD	WD	CD	WD	CD	WD	CD	WD
Steatosis	0.13 ± 0.35	2.33 ± 0.65	1 ± 0.74	2.69 ± 0.48	0 ± 0	2 ± 0.77	1.45 ± 0.52	2.17 ± 0.58
Inflammation	0.25 ± 0.46	1.75 ± 0.45	0.5 ± 0.52	1.31 ± 0.63	0.22 ± 0.44	1.45 ± 0.69	1.18 ± 0.4	2.08 ± 0.79
Ballooning	0 ± 0	1.83 ± 0.39	0 ± 0	1.31 ± 0.63	0 ± 0	0.91 ± 0.94	0 ± 0	1.67 ± 0.49
Total NAS	0.38 ± 0.74	5.92 ± 0.9	1.5 ± 0.8	5.31 ± 0.95	0.22 ± 0.44	4.36 ± 2.06	2.64 ± 0.67	5.92 ± 1.38
Fibrosis score	0 ± 0	1.83 ± 0.39	0.25 ± 0.45	1.77 ± 0.44	0 ± 0	1.55 ± 0.52	1 ± 0.45	2.08 ± 0.29

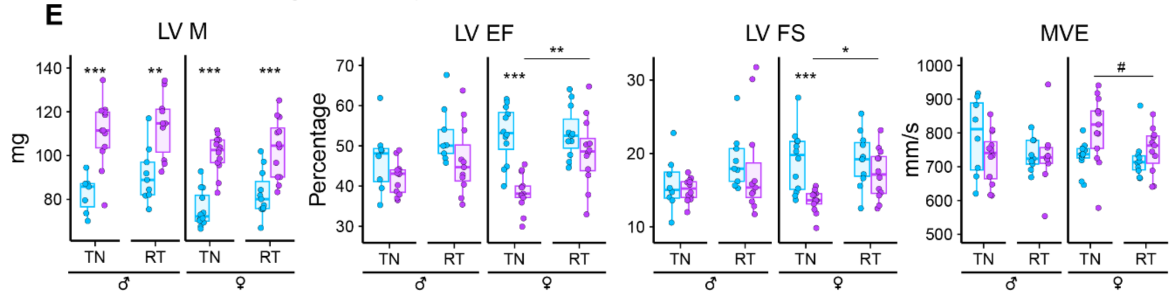

Supplementary Figure 2. Sex-specific effects of housing temperature on tissue weight and heart parameters in PWK/PhJ mice. (A) Kidney, quadriceps, soleus, gastrocnemius, and spleen weight normalized to body weight, expressed as a ratio (g/g). Abdominal fat (epiWAT) normalized to lean mass, expressed as a ratio (g/g). **(B)** Absolute liver and heart weight (g). **(C)** Percentage of steatosis quantified as a percentage of macro steatosis area for H&E staining in liver tissue. **(D)** Pathological scoring of liver H&E and SR-stained sections, presented as mean ± SD. Details of the scoring system, as well as the

calculation and statistical analysis of the NAS and fibrosis scores, are provided in Supplementary Table 2. **(E)** Echocardiography parameters. Results are shown as box-and-whisker plots. The lower and upper hinges correspond to the first quartile (25th percentile) and third quartile (75th percentile), with the median represented by a line in the center. The whiskers show the minimum and maximum values in the data. Points beyond the whiskers are outliers, plotted individually. For all graphs: TN-males, n= 7-8 CD, 12 WD; TN-females, n= 12 CD, 13 WD; RT-males, n= 9 CD, 11 WD; RT-females, n= 11 CD, 12 WD. For, A, B, C and E, statistical analysis was performed using a two-way ANOVA followed by Tukey's post hoc test. $^{\#}P < 0.1$, $^{*}P < 0.05$, $^{**}P < 0.01$, $^{***}P < 0.001$. For D, statistical analysis was conducted using a two-sided Student’s t-test with Benjamini–Hochberg adjusted p values.

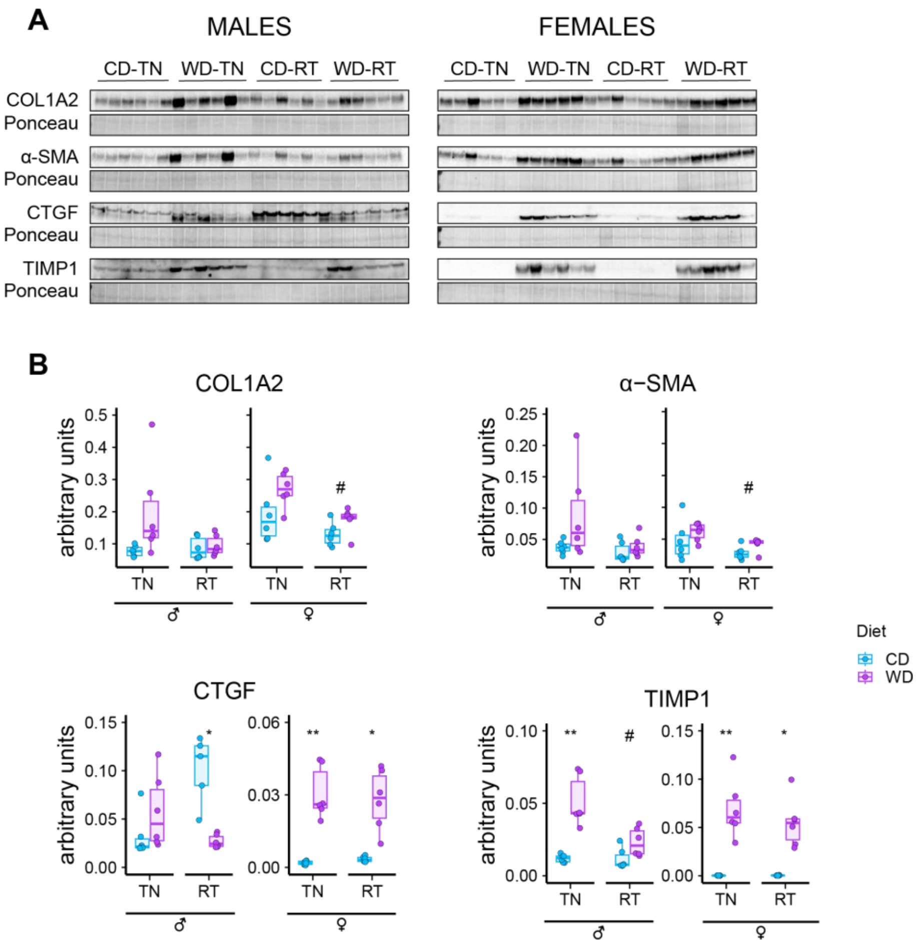

Supplementary Figure 3. Induction of liver fibrosis in PWK/PhJ mice after 17 weeks on a WD. Western blot analysis of liver tissue for COL1A2, α-SMA, CTGF and TIMP1 proteins (A) with corresponding band densitometry quantification normalized to Ponceau staining in arbitrary units (B). Results are shown as box-and-whisker plots. The lower and upper hinges correspond to the first quartile (25th percentile) and third quartile (75th percentile), with the median represented by a line in the center. The whiskers show the minimum and maximum values in the data. Points beyond the whiskers are outliers, plotted individually. TN-males, n= 6 CD, 6 WD; TN-females, n= 6 CD, 6 WD; RT-males, n= 6 CD, 6 WD; RT-females, n= 6 CD, 6 WD. For B, statistical analysis was conducted using a two-sided Student's t-test with Benjamini–Hochberg adjusted p values. #P < 0.1, *P < 0.05, **P < 0.01.

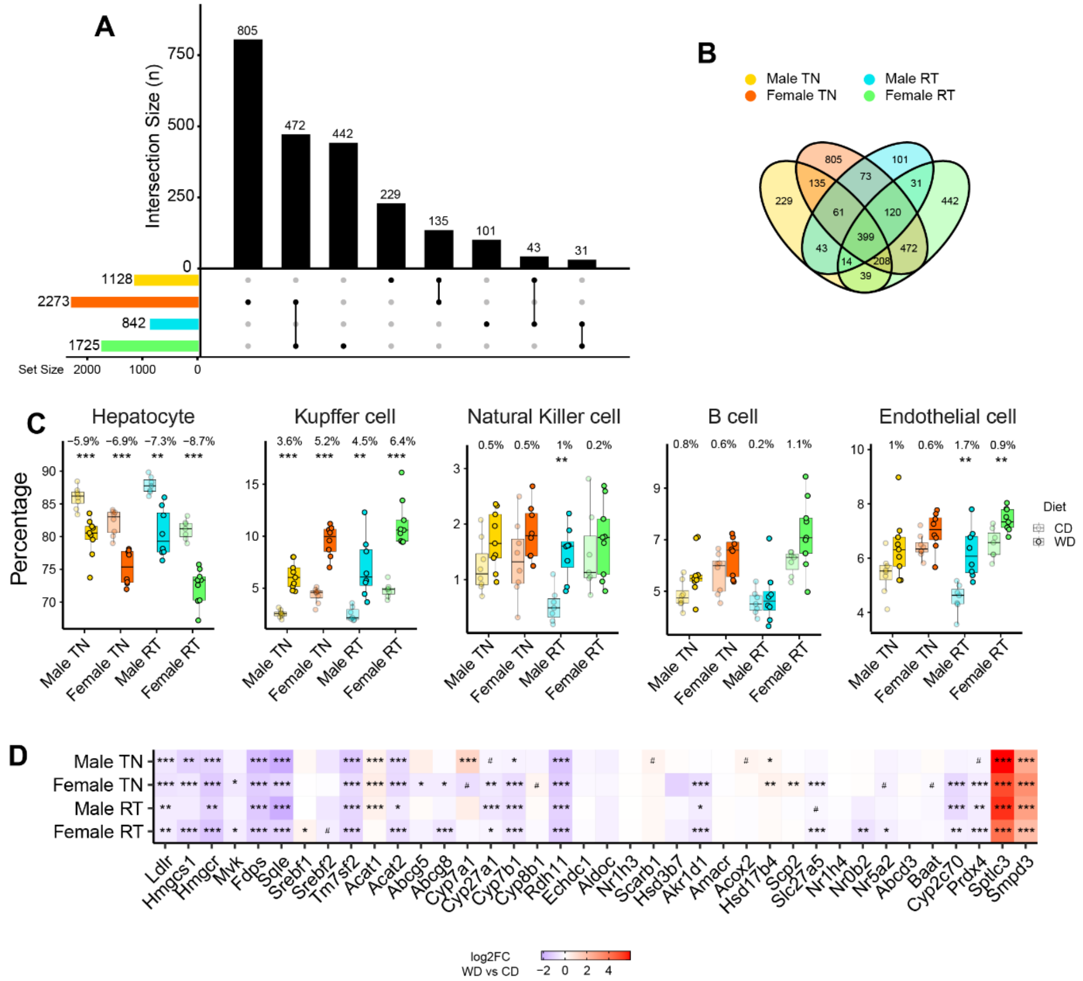

Supplementary Figure 4. Transcriptomic signatures in liver tissue. **(A)** UpSet plot showing the intersection size of liver DEGs (WD vs CD) across different groups (black bars), whose total number of DEGs is reported with the colored bars. **(B)** Venn diagram of liver transcriptomics showing the number of genes on WD overlapping between the four groups. **(C)** Percentages of hepatocytes, Kupffer cells, Natural Killer cells, B cells and endothelial cells in the liver samples, estimated by single-cell deconvolution analysis. **(D)** Transcriptomic WD vs CD log2 fold changes for genes associated with cholesterol and lipid pathways. For C, results are shown as box-and-whisker plots. The lower and upper hinges correspond to the first quartile (25th percentile) and third quartile (75th percentile), with the median represented by a line in the center. The whiskers show the minimum and maximum values in the data. Statistical analysis was performed using a two-sided Student's t-test. Points beyond the whiskers are outliers, plotted individually. TN-males, n= 8 CD, 9 WD; TN-females, n= 8 CD, 8 WD; RT-males, n= 7 CD, 8 WD; RT-females, n= 7 CD, 9 WD. For C and D, statistical analysis was conducted using a two-sided Student's t-test with Benjamini–Hochberg adjusted p values. $^{\#}P < 0.1$, $^{*}P < 0.05$, $^{**}P < 0.01$, $^{***}P < 0.001$.

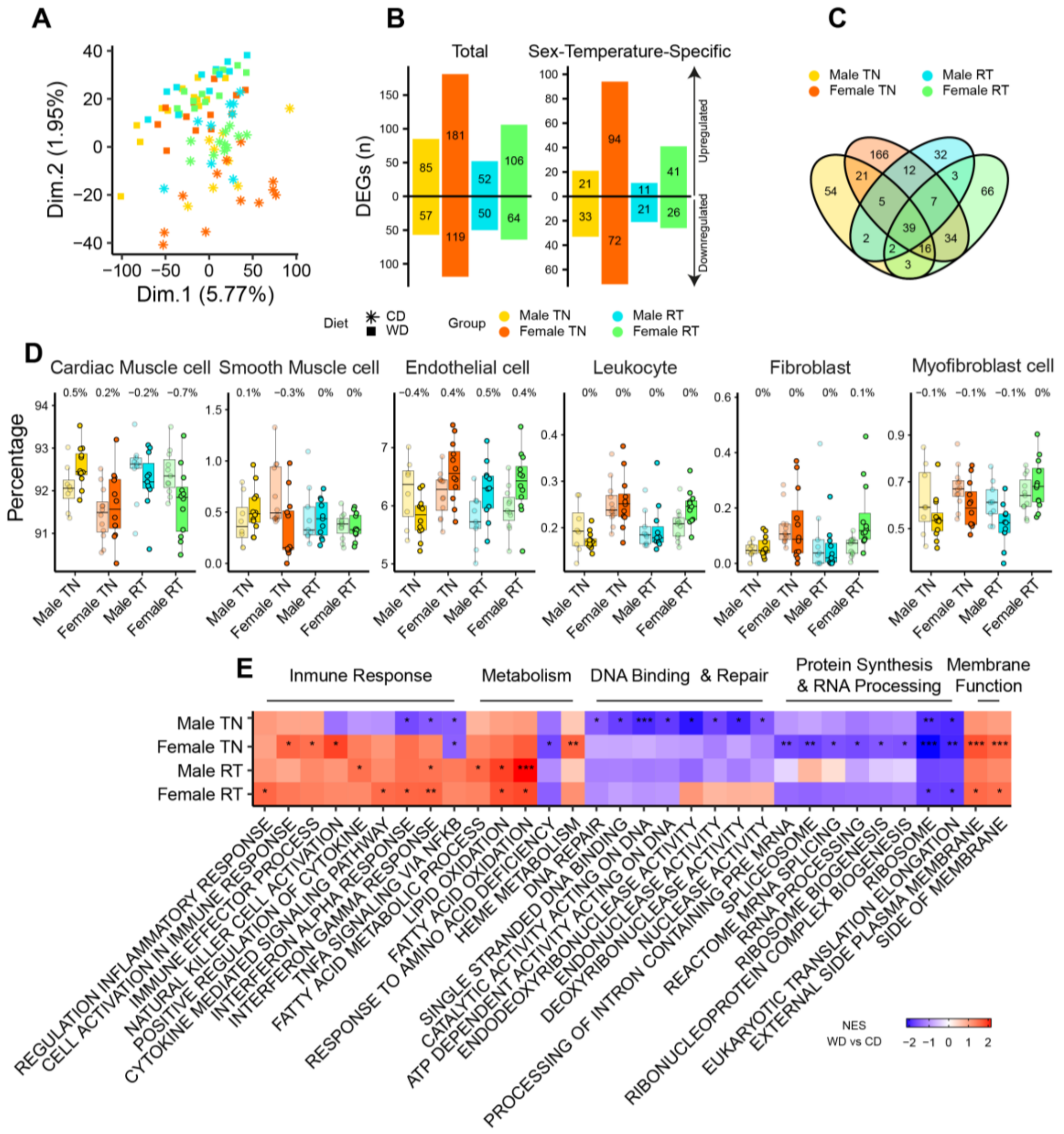

Supplementary Figure 5. Heart transcriptomic response to different housing temperatures. (A) PCA of CPM normalized heart expression data. **(B)** Total (left) and sex-temperature-specific (right) number of genes significantly up- or downregulated upon WD compared to CD in the heart for each experimental group. **(C)** Venn diagram of heart transcriptomics showing the number of genes on WD overlapping between the four groups. **(D)** Percentages of cardiac muscle cells, smooth muscle cells, endothelial cells, leukocytes, fibroblast and myofibroblast in the heart, estimated by single-cell deconvolution analysis. **(E)** GSEA results for representative gene sets relevant for MASH. TN-males, n= 8 CD, 12 WD; TN-females, n= 12 CD, 12 WD; RT-males, n= 9 CD, 11 WD; RT-females, n= 11 CD, 12 WD; For D and E, statistical analysis was conducted using a two-sided Student's t-test with Benjamini–Hochberg adjusted p values. *P < 0.05, **P < 0.01, ***P < 0.001.

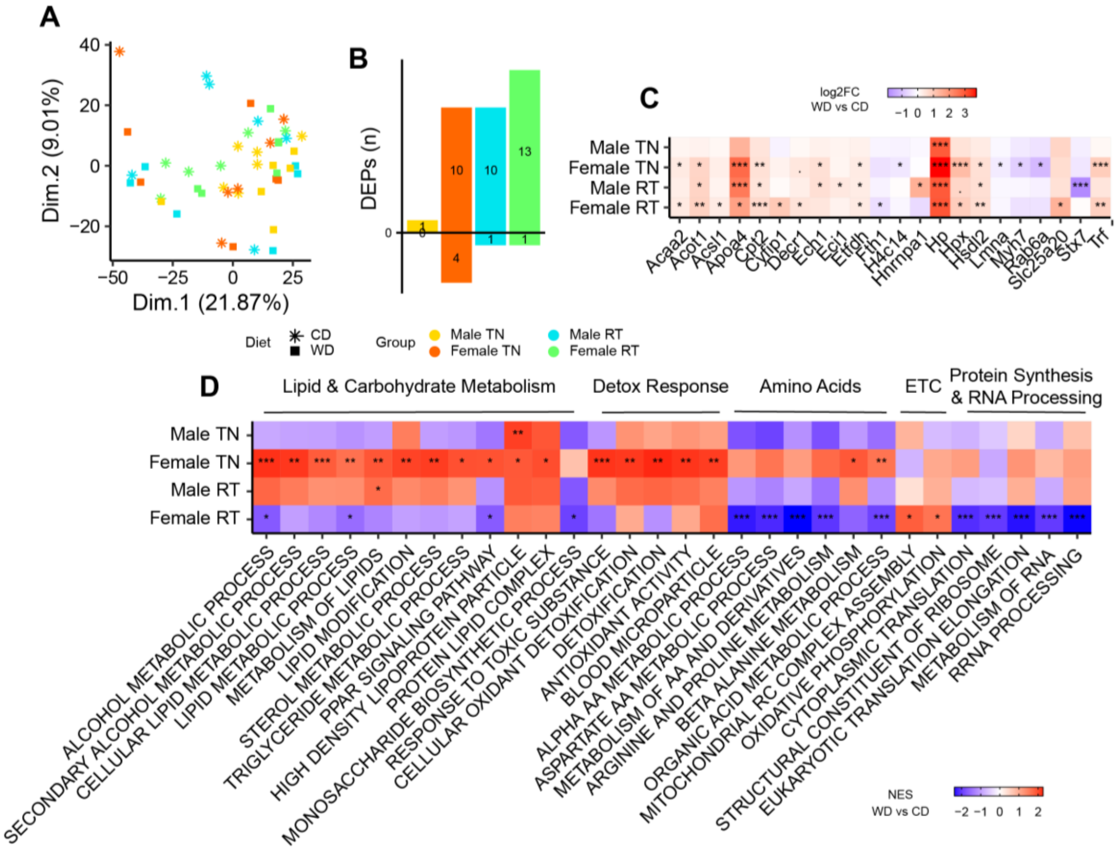

Supplementary Figure 6. Proteomic analysis of heart under different housing temperatures. (A) PCA on vsn-normalized LFQ intensity data from heart samples. **(B)** Total number of proteins significantly up- or downregulated upon WD compared to CD in heart for each experimental group. (C) Proteins that significantly up- or downregulated among the four groups. (D) Gene Set Enrichment Analysis (GSEA) performed on proteomics data. The results show a number of representative protein sets relevant for MASH. TN-males, n= 6 CD, 6 WD; TN-females, n= 6 CD, 6 WD; RT-males, n= 5 CD, 6 WD; RT-females, n= 6 CD, 6 WD. For D, statistical analysis was conducted using a two-sided Student's t-test with Benjamini–Hochberg adjusted p values. $^{\#}P < 0.1$, $^{*}P < 0.05$, $^{**}P < 0.01$, $^{***}P < 0.001$.

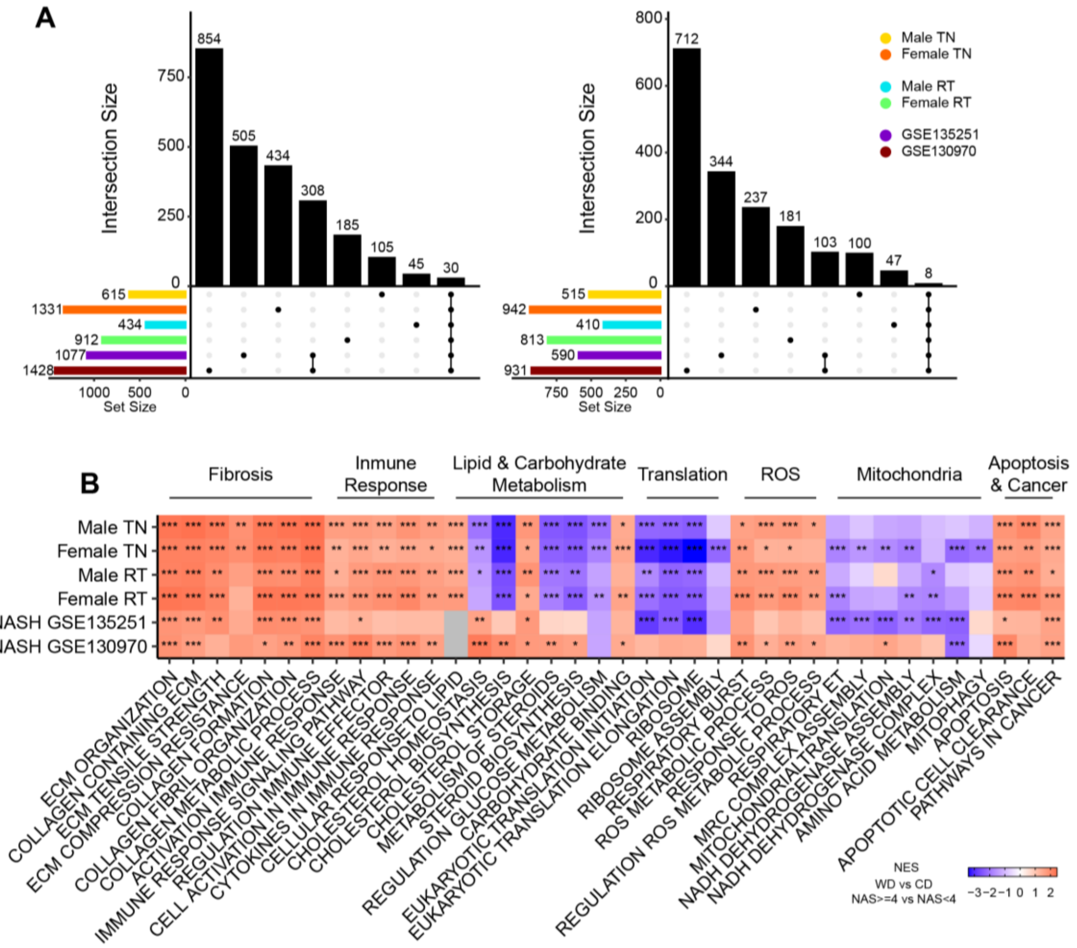

Supplementary Figure 7. Mouse liver transcriptomic signatures correlate with MASLD/MASH severity in humans. (A) UpSet plot showing the intersection sizes of liver DEGs (WD vs CD) across different groups (black bars), whose total number of DEGs is reported with the colored bars. **(B)** GSEA results for representative gene sets. TN-males, n= 8 CD, 9 WD; TN-females, n= 8 CD, 8 WD; RT-males, n= 7 CD, 8 WD; RT-females, n= 7 CD, 9 WD; GSE135251, n= 68 NAS<4, n= 148 NAS>= 4; GSE130970, n= 36 NAS<4, n= 42 NAS>= 4. Statistical analysis was conducted using a two-sided Student's t-test with Benjamini–Hochberg adjusted p values. *P < 0.05, **P < 0.01, ***P < 0.001.

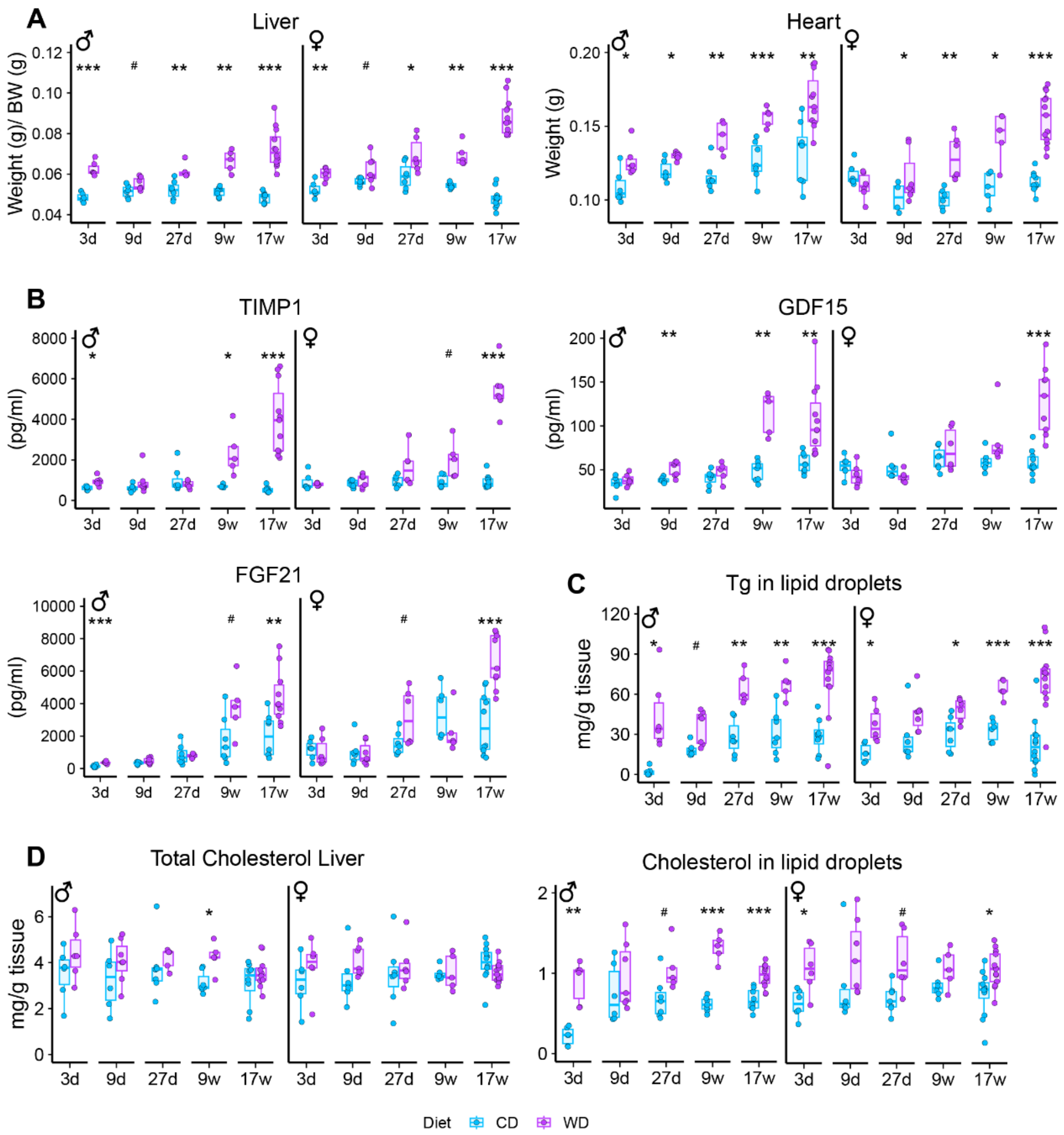

Supplementary Figure 8. Longitudinal phenotyping of PWK/PhJ mice housed at TN. (A) Liver weight normalized to body weight, expressed as a ratio (g/g) and total heart weight (g). 3d: TN-males, n= 6 CD, 6 WD; TN-females, n= 6 CD, 6 WD; 9d: TN-males, n= 6 CD, 6WD; TN-females, n= 6 CD, 6 WD; 27d: TN-males, n= 7 CD, 5 WD; TN-females, n= 7 CD, 6 WD; 9w: TN-males, n= 7 CD, 5 WD; TN-females, n= 6 CD, 5 WD; 17w: TN-males, n= 8 CD, 12 WD; TN-females, n= 12 CD, 13 WD**. (B)** TIMP1, GFD15 and FGF21 plasma levels. 3d: TN-males, n= 6 CD, 6 WD; TN-females, n= 6 CD, 6 WD; 9d: TN-males, n= 6 CD, 6WD; TN-females, n= 6 CD, 6 WD; 27d: TN-males, n= 7 CD, 5 WD; TN-females, n= 7 CD, 6 WD; 9w: TN-males, n= 7 CD, 5 WD; TN-females, n= 6 CD, 5 WD; 17w: TN-males, n= 8 CD, 11 WD; TN-females, n= 10 CD, 9 WD**. (C)** Triglycerides in liver lipid droplets (mg/ g liver tissue). 3d: TN-males, n= 6 CD, 6 WD; TN-females, n= 6 CD, 6 WD; 9d: TN-males, n= 6 CD, 7WD; TN-females, n= 6 CD, 7 WD; 27d: TN-males, n= 7 CD, 7 WD; TN-females, n= 5 CD, 6 WD; 9w: TN-males, n= 7 CD, 6 WD; TN-females, n= 5 CD, 5 WD; 17w: TN-males, n= 8 CD, 12 WD; TN-females, n= 12 CD, 13 W. **(D)** Total cholesterol in the liver and cholesterol content in liver lipid droplets (mg/ g liver tissue). 3d: TN-males, n= 6 CD, 6 WD; TN-females, n= 6 CD, 6 WD; 9d: TN-males, n= 6 CD, 7WD; TN-females, n= 6 CD, 7 WD; 27d: TN-males, n= 7 CD, 7 WD; TN-females, n= 5 CD, 6 WD; 9w: TN-males, n= 7 CD, 6 WD; TN-females, n= 5 CD, 5 WD; 17w: TN-males, n=

8 CD, 12 WD; TN-females, n= 12 CD, 13 WD. Results are shown as box-and-whisker plots. The lower and upper hinges correspond to the first quartile (25th percentile) and third quartile (75th percentile), with the median represented by a line in the center. The whiskers show the minimum and maximum values in the data. Points beyond the whiskers are outliers, plotted individually. 3d: TN-males, n= 6 CD, 6WD; TN-females, n= 6 CD, 6 WD; 9d: TN-males, n= 6 CD, 6WD; TN-females, n= 6 CD, 6 WD; 27d: TN-males, n= 7 CD, 5 WD; TN-females, n= 7 CD, 6 WD; 9w: TN-males, n= 7 CD, 5 WD; TN-females, n= 6 CD, 5 WD; 17w: TN-males, n= 8 CD, 8-12 WD; TN-females, n= 8-12 CD, 8-13 WD. Statistical analysis was conducted using a two-sided Student’s t-test with Benjamini–Hochberg adjusted p values. $^{\#}P < 0.1$, $^{*}P < 0.05$, $^{**}P < 0.01$, $^{***}P < 0.001$.

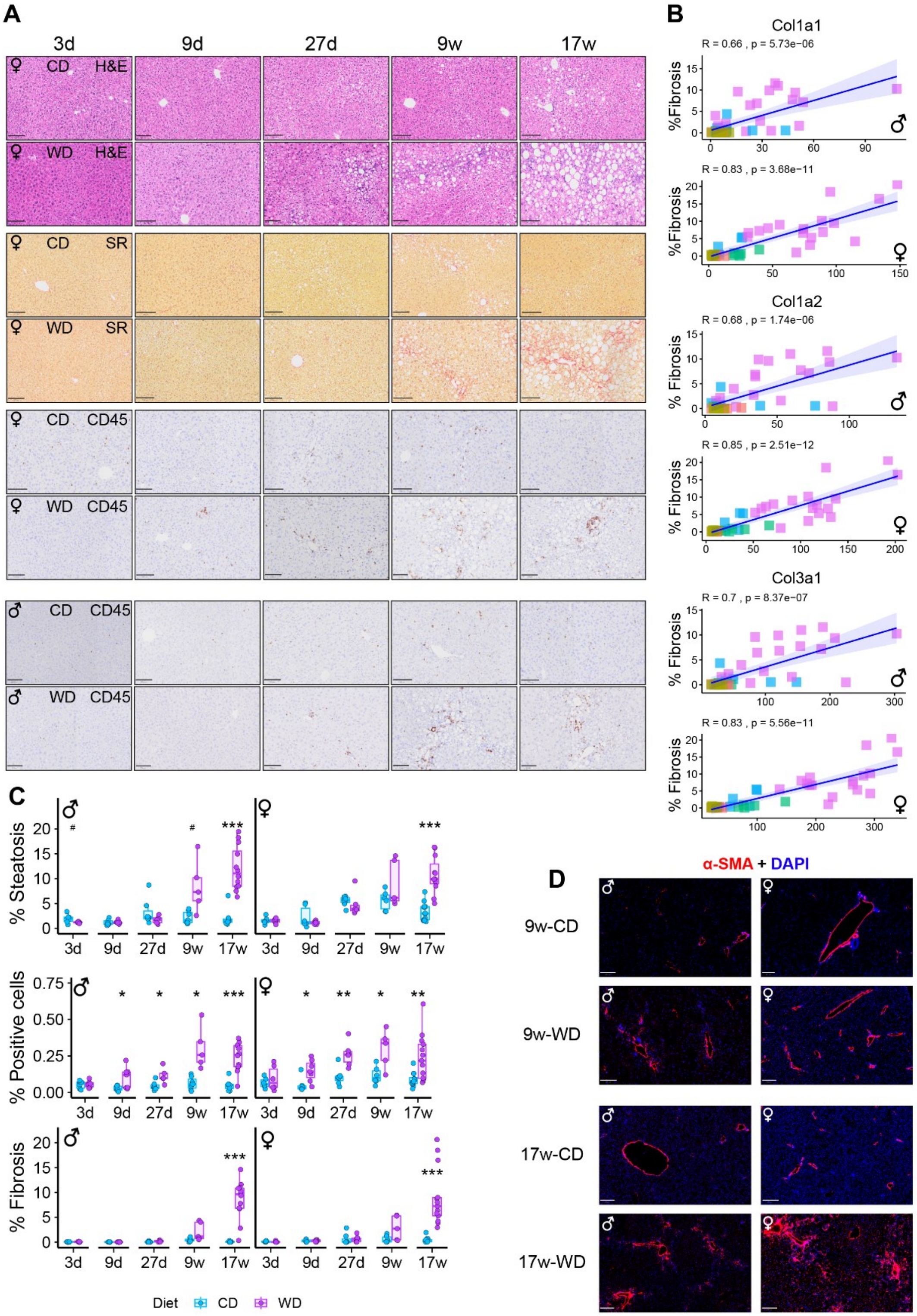

Supplementary Figure 9. Longitudinal phenotyping of PWK/PhJ mice housed at TN. (A) Representative H&E and SR staining images of formalin-fixed liver sections of female animals (Fig. 6 for males) and CD45 staining of males and females. Scale Bar = 100 µm. **(B)** Correlation analysis between liver fibrosis and collagen gene expression, with Pearson's R and P-value indicated. **(C)** Percentage of steatosis quantified as a percentage of macro steatosis per area for H&E staining, percentage of positive

CD45+ immune cells quantified per area for CD45 staining and percentage of fibrosis quantified as a positive area for SR staining. **(D)** Immunofluorescence staining for alpha-smooth muscle actin (α-SMA) in liver sections. Scale Bar = 200 µm. C, results are shown as box-and-whisker plots. The lower and upper hinges correspond to the first quartile (25th percentile) and third quartile (75th percentile), with the median represented by a line in the center. The whiskers show the minimum and maximum values in the data. Points beyond the whiskers are outliers, plotted individually. 3d: TN-males, n= 6 CD, 6WD; TN-females, n= 6 CD, 6 WD; 9d: TN-males, n= 6 CD, 6WD; TN-females, n= 6 CD, 6 WD; 27d: TN-males, n= 7 CD, 5 WD; TN-females, n= 7 CD, 6 WD; 9w: TN-males, n= 7 CD, 5 WD; TN-females, n= 6 CD, 5 WD; 17w: TN-males, n= 8 CD, 12 WD; TN-females, n= 12 CD, 13 WD. Statistical analysis was performed using a two-sided Student's t-test. $^{\#}P < 0.1$, $^{*}P < 0.05$, $^{**}P < 0.01$, $^{***}P < 0.001$.

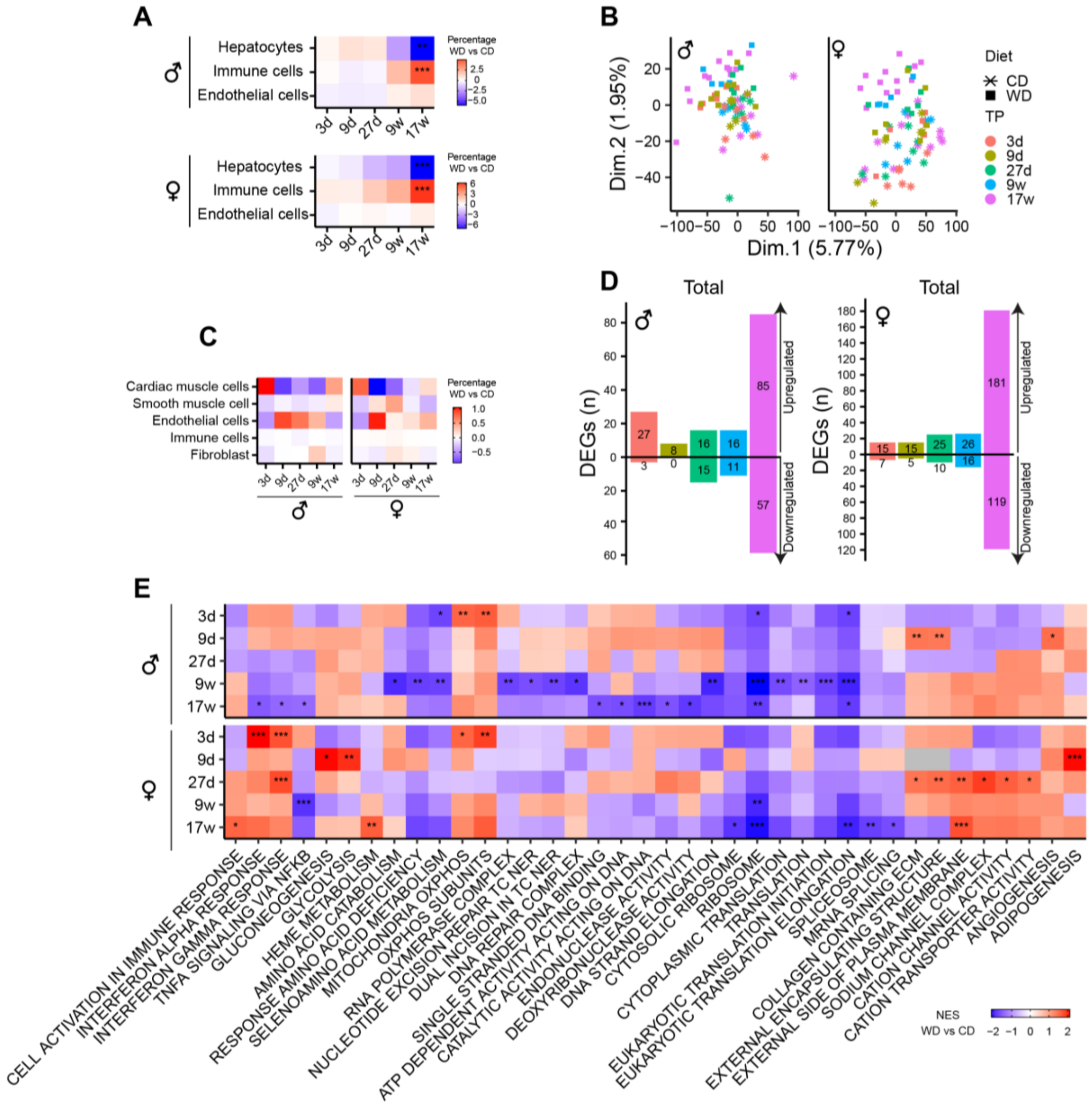

Supplementary Figure 10. Longitudinal transcriptome analysis of the heart shows no major changes in transcript levels. (A) Percentages of hepatocytes, immune cells, and endothelial cells in the liver, estimated by single-cell deconvolution analysis. **(B)** PCA of CPM normalized heart expression data. **(C)** Percentages of cardiac muscle cells, smooth muscle cells, endothelial cells, immune cells and fibroblast in the heart estimated by single-cell deconvolution analysis. **(D)** Total number of heart genes significantly up- or downregulated upon WD compared to CD for males (left) and females (right) in each experimental group. **(E)** GSEA results for representative gene sets relevant for MASH. For heart, 3d: TN-males, n= 6 CD, 6 WD; TN-females, n= 6 CD, 6 WD; 9d: TN-males, n= 5 CD, 8 WD; TN-females, n= 6 CD, 7 WD; 27d: TN-males, n= 7 CD, 5 WD; TN-females, n= 7 CD, 6 WD; 9w: TN-males, n= 7 CD, 5 WD; TN-females, n= 6 CD, 5 WD; 17w: TN-males, n= 8 CD, 12 WD; TN-females, n= 12 CD, 12 WD. For A, C and E, statistical analysis was conducted using a two-sided Student's t-test with Benjamin–Hochberg adjusted p values. *P < 0.05, **P < 0.01, ***P < 0.001.

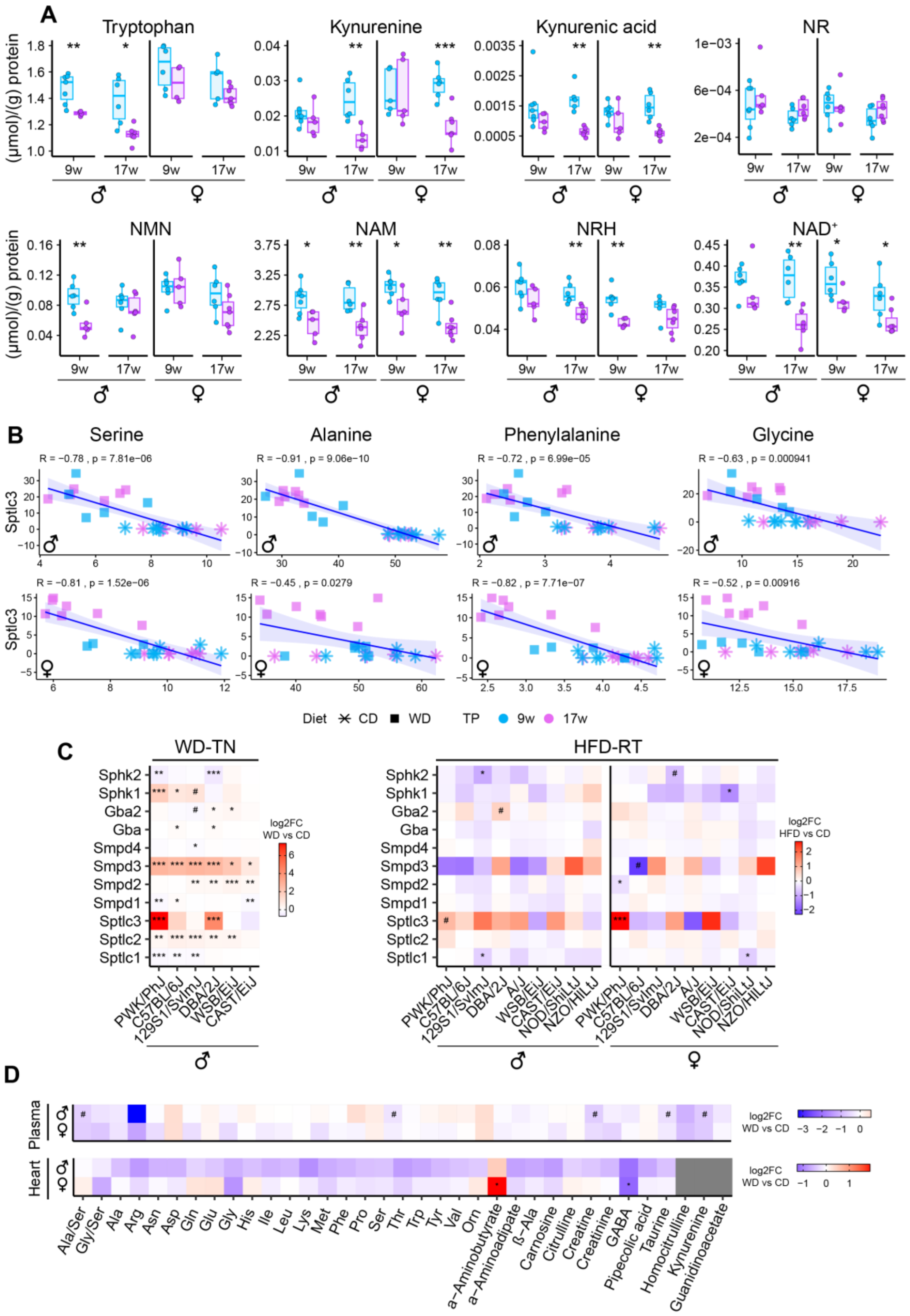

Supplementary Figure 11. Liver disease in the PWK/PhJ mouse model under a WD is characterized by disrupted amino acid and sphingolipid metabolism. (A) Abundance of metabolites in the NAD⁺

pathway in the liver. **(B)** Correlation analysis between the *Sptlc3* gene and the amino acids serine, alanine, phenylalanine and glycine, with Pearson's R and P-value indicated. **(C)** Liver transcriptomics in males WD *vs* CD (left) and males and females HFD *vs* CD (right). log2 fold changes for genes associated with sphingolipid metabolic pathways in the different mouse strains are indicated. **(D)** WD vs CD log2 fold metabolomic changes for amino acid, organic acids and related amino acid metabolites in plasma and heart tissue. 9w: TN-males, n= 7 CD, 5 WD; TN-females, n= 6 CD, 5 WD; 17w: TN-males, n= 6 CD, 6 WD; TN-females, n= 6 CD, 7 WD. Statistical analysis was conducted using a two-sided Student's t-test with Benjamini–Hochberg adjusted p values. $^{\#}P < 0.1$, $^{*}P < 0.05$, $^{**}P < 0.01$, $^{***}P < 0.001$.

MALES

Ponceau | Overlap with maker | Chemiluminescence

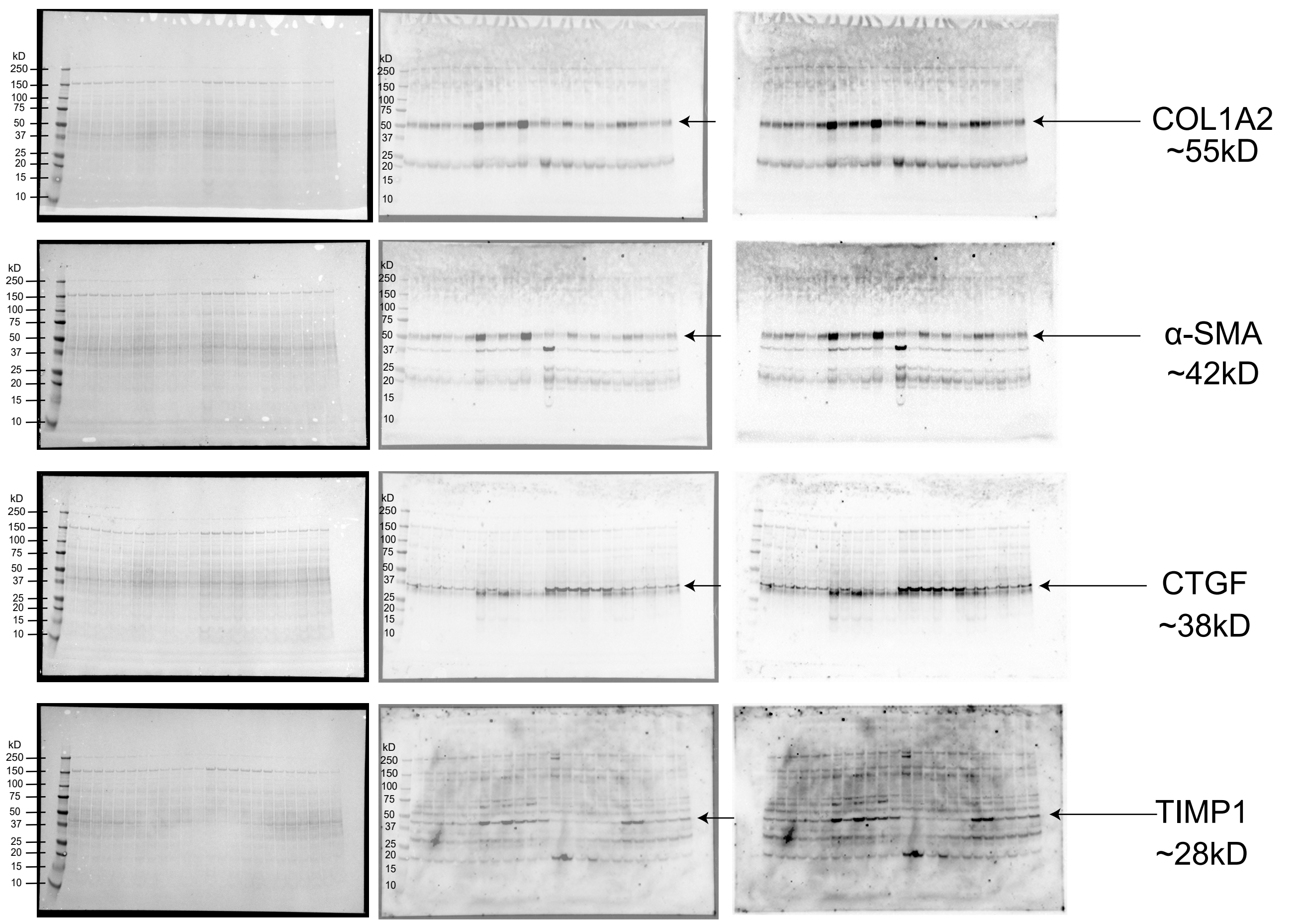

FEMALES

Ponceau | Overlap with maker | Chemiluminescence

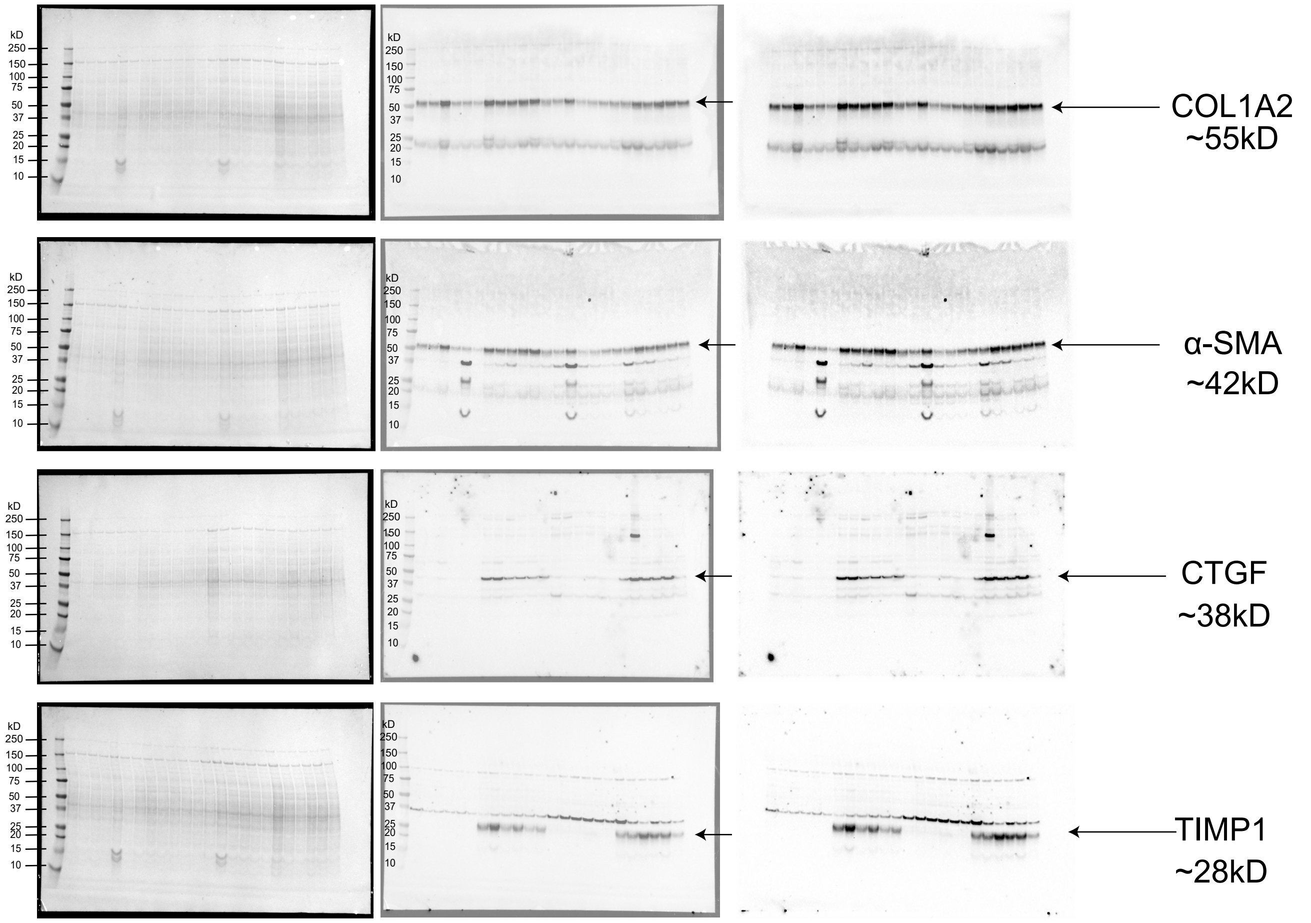

Supplement: Supplementary file 1 — Supplementary Information [file 41467_2026_73449_MOESM1_ESM.pdf]
