# Supplementary material for: Western diet-induced MASH in PWK/PhJ mice identifies disruptions in amino acid and sphingolipid metabolism contributing to cardiac dysfunction
Source: Nat Commun. 2026 May 20;17:6629. doi: 10.1038/s41467-026-73449-7 (PMC13381867; doi:10.1038/s41467-026-73449-7)
Supplement: Supplementary file 2 — Description of Additional Supplementary Information [file 41467_2026_73449_MOESM2_ESM.pdf]

Description of Additional Supplementary Information

Title: Supplementary Data 1.

Description: The Supplementary Data 1 includes: (1.1) the list of animals used in the study, (1.2) plasma biochemistry data, (1.3) insulin measurements and (1.4) Luminex analyses performed with GDF-15, TIMP-1 and FGF-21 markers.

Title: Supplementary Data 2.

Description: Supplementary Data 2 includes NAS and fibrosis scoring data: (2.1) the scoring scheme used, (2.2) the list of animals, (2.3) individual scores for steatosis, inflammation, ballooning, total NAS and fibrosis. In (2.4) mean, SD and SEM per experimental group, (2.5) summary tables of steatosis, inflammation, ballooning, total NAS and fibrosis scores (mean ± SD), (2.6) t-test results comparing WD versus CD, (2.7) t-test results comparing males versus females and finally (2.8) t-test results comparing TN versus RT.

Title: Supplementary Data 3.

Description: Supplementary Data 3 comprises the experimental settings for targeted metabolomics analyses: (3.1–3.4) sphingolipid-related measurements, including (3.1) the names of standards and their corresponding isotopically labelled internal standards, (3.2) the standard concentrations used across all calibrators for calibration curve generation together with the correlation coefficients, (3.3) coefficients of variation (CVs) obtained from pooled quality control samples representative of the full batch for each species and (3.4) the MRM transitions and instrumental parameters used for targeted analysis. Sections (3.5–3.8) detail the amino acid targeted metabolomics experimental settings adapted from Teav T et al (2019). Anal Chem. Sep 17;91(18):11757-11769. doi: 10.1021/acs.analchem.9b02373. These sections include (3.5) the names of standards and their corresponding isotopically labelled internal standards, (3.6) the standard concentrations used across all calibrators for calibration curve generation together with the correlation coefficients, (3.7) coefficients of variation (CVs) obtained from pooled quality control samples representative of the full batch for each species and (3.8) the MRM transitions and instrumental parameters used for targeted analysis. Sections (3.9–3.12) describe the NAD^+ metabolome targeted metabolomics experimental settings adapted from van der Velpen et al (2021). J Neurochem. Oct;159(2):378-388. doi: 10.1111/jnc.15362. These section include (3.9) the names of standards and their corresponding isotopically labelled internal standards, (3.10) the standard concentrations used across all calibrators for calibration curve generation together with the correlation coefficients, (3.11) coefficients of variation (CVs) obtained from pooled quality control samples representative of the full batch for each species and (3.12) the MRM transitions and instrumental parameters used for targeted analysis.

Title: Supplementary Data 4.

Description: Supplementary Data 4 contains targeted metabolomics analyses of sphingolipids, including: (4.1) the list of animals, (4.2) liver analyses, (4.3) heart analyses and (4.4) plasma analyses.

Title: Supplementary Data 5.

Description: Supplementary Data 5 contains targeted metabolomics analyses of amino acids, including: (5.1) the list of animals, (5.2) liver analyses, (5.3) heart analyses and (5.4) plasma analyses.

Title: Supplementary Data 6.

Description: Supplementary Data 6 contains targeted metabolomics analyses of NAD+ metabolome, including: (6.1) the list of animals, (6.2) liver analyses.
