# Supplementary material for: Western diet-induced MASH in PWK/PhJ mice identifies disruptions in amino acid and sphingolipid metabolism contributing to cardiac dysfunction
Source: Nat Commun. 2026 May 20;17:6629. doi: 10.1038/s41467-026-73449-7 (PMC13381867; doi:10.1038/s41467-026-73449-7)
Supplement: Supplementary file 9 — Reporting Summary [file 41467_2026_73449_MOESM9_ESM.pdf]

nature portfolio

Corresponding author(s): Johan Auwerx
Sandra Rodríguez López

Last updated by author(s): Apr 20, 2026

Reporting Summary

Nature Portfolio wishes to improve the reproducibility of the work that we publish. This form provides structure for consistency and transparency in reporting. For further information on Nature Portfolio policies, see our Editorial Policies and the Editorial Policy Checklist.

Statistics

For all statistical analyses, confirm that the following items are present in the figure legend, table legend, main text, or Methods section.

n/a	Confirmed	
☐	☒	The exact sample size (*n*) for each experimental group/condition, given as a discrete number and unit of measurement
☐	☒	A statement on whether measurements were taken from distinct samples or whether the same sample was measured repeatedly
☐	☒	The statistical test(s) used AND whether they are one- or two-sided<br>*Only common tests should be described solely by name; describe more complex techniques in the Methods section.*
☐	☒	A description of all covariates tested
☐	☒	A description of any assumptions or corrections, such as tests of normality and adjustment for multiple comparisons
☐	☒	A full description of the statistical parameters including central tendency (e.g. means) or other basic estimates (e.g. regression coefficient) AND variation (e.g. standard deviation) or associated estimates of uncertainty (e.g. confidence intervals)
☐	☒	For null hypothesis testing, the test statistic (e.g. *F*, *t*, *r*) with confidence intervals, effect sizes, degrees of freedom and *P* value noted<br>*Give P values as exact values whenever suitable.*
☒	☐	For Bayesian analysis, information on the choice of priors and Markov chain Monte Carlo settings
☒	☐	For hierarchical and complex designs, identification of the appropriate level for tests and full reporting of outcomes
☐	☒	Estimates of effect sizes (e.g. Cohen's *d*, Pearson's *r*), indicating how they were calculated

Our web collection on statistics for biologists contains articles on many of the points above.

Software and code

Policy information about availability of computer code

Data collection	- Body composition was assessed using Echo-MRI machine (the 3-in-1, Echo Medical Systems)<br>- Indirect calorimetry was performed using the metabolic and behavioral phenotyping system (Promethion, Sable Systems)<br>- Transthoracic echocardiography was performed using a Vevo 2100 system (FUJIFILM VisualSonics, Toronto, Canada) with a 40-MHz transducer.<br>- Proteomics were performed on a Vanquish Neo nano UPLC system (ThermoFisher Scientific) connected on-line with an Exploris 480 Orbitrap mass spectrometer (ThermoFisher Scientific). Proteomics raw files were acquired using Thermo instruments and processed with MaxQuant v2.4.4.0.<br>- Histological images were taken with VS120 Slide Scanner (Olympus).<br>- Western Blots images were taken with a ChemiDoc Imaging System (Bio-Rad, USA).
Data analysis	R (version 4.1.0) through RStudio was used<br>Differential expression analysis was conducted using limma-voom (version 3.50.3)<br>GSEA was performed using clusterProfiler R package (version 4.2.2)<br>Single-cell deconvolution analysis were performed using MuSiC R package (version 1.0.0)<br>LFQ intensities were normalized using variance-stabilizing normalization (vsn) (version 3.76.0)<br>p-values were adjusted for multiple testing using the Benjamini–Hochberg procedure<br>Western blot band intensities were quantified with Image Lab 6.1 software (Bio-Rad, USA)

For manuscripts utilizing custom algorithms or software that are central to the research but not yet described in published literature, software must be made available to editors and reviewers. We strongly encourage code deposition in a community repository (e.g. GitHub). See the Nature Portfolio guidelines for submitting code & software for further information.

Data

Policy information about availability of data

All manuscripts must include a data availability statement. This statement should provide the following information, where applicable:

- Accession codes, unique identifiers, or web links for publicly available datasets
- A description of any restrictions on data availability
- For clinical datasets or third party data, please ensure that the statement adheres to our policy

Source Data are provided with this paper.
Our data are openly accessible through our online app (https://lisp-lms.shinyapps.io/PWK_study_app/) as a valuable resource for the research community, providing free access to explore the phenotyping, metabolic, lipidomic, and transcriptomic traits presented in this work.
BRB-seq data used in this study were deposited in the Gene Expression Omnibus (GEO) database (GSE309251) and proteomics raw data were deposited at PRoteomics IDEntifications (PRIDE) (Liver: PXD068560, Heart: PXD068595).

Research involving human participants, their data, or biological material

Policy information about studies with human participants or human data. See also policy information about sex, gender (identity/presentation), and sexual orientation and race, ethnicity and racism.

Reporting on sex and gender	NA
Reporting on race, ethnicity, or other socially relevant groupings	NA
Population characteristics	NA
Recruitment	NA
Ethics oversight	NA

Note that full information on the approval of the study protocol must also be provided in the manuscript.

Field-specific reporting

Please select the one below that is the best fit for your research. If you are not sure, read the appropriate sections before making your selection.

☒ Life sciences ☐ Behavioural & social sciences ☐ Ecological, evolutionary & environmental sciences

For a reference copy of the document with all sections, see nature.com/documents/nr-reporting-summary-flat.pdf

Life sciences study design

All studies must disclose on these points even when the disclosure is negative.

Sample size	No statistical method was used to determine sample size. Sample sizes were chosen based on studies with similar experimental conducted at the laboratory of Prof. Johan Auwerx.
Data exclusions	BRB-seq: Heart samples HDP-018715 and HDP-019057 were excluded from downstream analysis due to suspected sample swap.<br><br>Proteomics: Liver sample S_42 (sample ID HDP-018879, corresponding to a male CD-RT) was excluded from the downstream analysis due to suspected mislabeling with sample ID HDP-018897.
Replication	All the replicates represent biological replicates. The mice used for experiments were from multiple different litters.
Randomization	For all the in vivo experiments, groups were randomly assigned.
Blinding	Scientists performing the mouse phenotyping experiments were blinded to the experimental groups.

Reporting for specific materials, systems and methods

We require information from authors about some types of materials, experimental systems and methods used in many studies. Here, indicate whether each material, system or method listed is relevant to your study. If you are not sure if a list item applies to your research, read the appropriate section before selecting a response.

Materials & experimental systems

n/a	Involved in the study	
[ ]	[x]	Antibodies
[x]	[ ]	Eukaryotic cell lines
[x]	[ ]	Palaeontology and archaeology
[ ]	[x]	Animals and other organisms
[x]	[ ]	Clinical data
[x]	[ ]	Dual use research of concern
[x]	[ ]	Plants

Methods

n/a	Involved in the study	
[x]	[ ]	ChIP-seq
[x]	[ ]	Flow cytometry
[x]	[ ]	MRI-based neuroimaging

Antibodies

Antibodies used

For histology analysis:
- CD45 Monoclonal Antibody (30-F11) (Catalog # 14-0451-82, Thermofisher).
- ImmPRESS® HRP Goat Anti-Rat IgG, Mouse adsorbed Polymer Detection Kit, Peroxidase (MP-7444-15: Vector laboratories)
- Anti-Actin, α-Smooth Muscle - Cy3™ antibody, Mouse monoclonal (C6198, Sigma Aldrich)

For Western Blot analysis, primary antibodies:
- COL1A2 (Santa Cruz, sc-393573)
- α-Smooth Muscle (Invitrogen, 14-9760-82)
- TIMP1 (Abcam, ab179580)
- CTGF (Abcam, ab318148)

HRP-conjugated secondary antibodies:
- goat-anti-rabbit (Azure Biosystems, AC2114)
- goat-anti-mouse (Azure Biosystems, AC2115)

Validation

All the antibodies are commercial available and were validated by the manufactures.

Additionally, the histology facility validated the antibodies in multiple samples.

Animals and other research organisms

Policy information about studies involving animals; ARRIVE guidelines recommended for reporting animal research, and Sex and Gender in Research

Laboratory animals	PWK/PhJ from 7- 24 weeks old male and female mice were used
Wild animals	The study did not involve wild animals
Reporting on sex	Sex were considered in the experimental design using male and females PWK/PhJ mice.
Field-collected samples	The study did not involve samples collected from the field
Ethics oversight	All animal experiments were performed according to Swiss ethical guidelines and approved by the Service de la Consommation et des Affaires Vétérinaires of the Canton de Vaud (license VD3598)

Note that full information on the approval of the study protocol must also be provided in the manuscript.

Plants

Seed stocks	NA
Novel plant genotypes	NA
Authentication	NA
