# Supplementary material for: Western diet-induced MASH in PWK/PhJ mice identifies disruptions in amino acid and sphingolipid metabolism contributing to cardiac dysfunction
Source: Nat Commun. 2026 May 20;17:6629. doi: 10.1038/s41467-026-73449-7 (PMC13381867; doi:10.1038/s41467-026-73449-7)
Supplement: Supplementary file 10 — Transparent Peer Review file [file 41467_2026_73449_MOESM10_ESM.pdf]

nature portfolio

Peer Review File

Western diet-induced MASH in PWK/PhJ mice identifies disruptions in amino acid and sphingolipid metabolism contributing to cardiac dysfunction

Corresponding Author: Professor Johan Auwerx

This file contains all reviewer reports in order by version, followed by all author rebuttals in order by version.

Version 0:

Reviewer comments:

Reviewer #1

(Remarks to the Author)
Although the design of this study is very systematic and meaningful data has been obtained, the hypothetical conclusions drawn lack validation and belong to descriptive articles. Based on this, I do not believe it has reached the level represented by the journal.

Reviewer #2

(Remarks to the Author)
In this work, López et al. explore the metabolic alterations in western diet-induced MASH. The authors found that amino acids and sphingolipids changed significantly in MASH and that there were some differences between genders. When performing metabolomics analysis, I think there are some problems, especially the identification and quantification of metabolites. This is very critical and maybe influence experimental conclusions.

(1) The impact of housing temperature on diet-induced MASH. Why did the authors set only the room temperature group (RT, 24 °C) and the thermoelectric group (TN, 30 °C), and not the other temperature groups? The effect of temperature on metabolism is obvious. Too high or too low temperature will inevitably affect the metabolic states of the organism. Did the authors take into account the effect of temperature in their metabolic assays for amino acids and sphingolipids.

(2) The author conducted absolute quantitative analysis on both sphingolipids and amino acids, but I did not find information on standard curves and quality control samples, as well as specific quantitative data, correlation coefficients, etc. from the raw data provided by the author (https://lisp-lms.shinyapps.io/PWK_study_app/). What isotopic internal standards did the author use?

(3) Figure 7B. The authors accurately quantified the contents and variations of different sphingolipid species, and the authors even made judgments about the composition of fatty acid side chains and cis-trans isomers. How did the authors make the identification? MS/MS analysis was performed? How were the cis-trans isomers determined?

(4) Chronic WD challenge impacts amino acid and sphingolipid metabolism. The authors performed metabolomics analysis. Non-targeted or targeted metabolomics analysis? If it is a non-targeted metabolomics analysis, how did the author select amino acids as differential variables from thousands of variables? If it was a targeted analysis, did the authors determine that the amino acids and sphingolipids would change before the experiment began?

(5) In this manuscript, the authors identified abnormalities in the metabolism of amino acids and sphingolipid, but further validation is needed to determine whether the metabolic abnormalities are related to MASLD and MASH, and whether the amino acids and sphingolipids are the cause of MASLD and MASH or merely a phenotype of altered metabolism in MASLD and MASH.

(6) How does cholesterol change in the liver? The authors only examined its changes in plasma, and if the authors had performed a metabolomics study, they should have been able to obtain information on how cholesterol changes in the liver.

Because, elevated liver cholesterol levels are common in MASH patients. In addition, changes in amino acids and sphingolipids in plasma should be of interest.

(7) Was absolute quantification also performed for the organic acids in Figure 7A? How this data was obtained is not explained by the authors.

Reviewer #3

(Remarks to the Author)
Rodríguez-López et al. report their comprehensive investigation into the cardiometabolic phenotypes of male and female PWK/PhJ mice housed at room temperature (RT) or thermoneutrality (TN) for up to 17 weeks, focusing on the development of metabolic dysfunction-associated steatohepatitis (MASH) and cardiac dysfunction. Given that cardiovascular disease (CVD) is the leading cause of death in patients with MASH, and there is a lack of mouse models that accurately replicate both conditions, this study highlighting Western diet (WD)-fed PWK/PhJ mice as a model suitable for the study of cardiac dysfunction in MASH is both important and significant. Strengths of this study include comprehensive analyses of livers, plasma, cardiac function, systemic energy metabolism, and body composition, with comparisons based on sex and housing temperature. The comparison of mouse and human liver transcriptomics and the metabolomics studies highlighting dysregulated amino acid and sphingolipid metabolism are additional strengths. The results of the longitudinal studies, demonstrating a strong MASH-fibrosis phenotype along with cardiac dysfunction after just 17 weeks on the WD, especially in females, could be highly valuable to the field, as current models typically require longer feeding periods. Despite these strengths, the study has several limitations that warrant further attention and investigation as outlined below.

Comparisons to male and female C57BL/6J mice is necessary to support key claims: In their recent study (PMID: 36787127), the authors reported that the PWK/PhJ strain is highly susceptible to WD-induced MASH compared to other mouse strains, including the widely used C57BL/6J. That study included only male mice and did not examine cardiac function. In the current study, both male and female PWK/PhJ mice were included, and both MASH and cardiac functions were assessed. However, comparison to the commonly used C57BL/6J mouse strain was lacking. While most studies using C57BL/6J mice report more severe MASH in males, recent research has shown an opposite outcome (PMID: 39911943). Moreover, sex-specific differences in MASH-associated cardiac function in various mouse strains have not been studied. Thus, endpoint analyses should be compared between male and female PWK/PhJ mice and C57BL/6J mice to support the authors' claim that MASH-fibrosis and cardiac dysfunction are accelerated in PWK/PhJ mice.

The manuscript offers limited insight into the mechanisms linking MASH and CVD: The authors report cardiac dysfunction in WD-fed PWK/PhJ mice, with notably more severe effects in females. However, RNA-seq of the hearts showed minimal changes. Moreover, metabolomics highlighting dysregulated amino acid and sphingolipid metabolism was performed only on livers with no attempt to link the findings to cardiac function. Are similar metabolic changes observed in plasma and the heart tissue? Are VLDL particles of WD-fed PWK/PhJ mice contain higher ceramides (in line with Ref. 50)? Alternatively, are changes in amino acids promote cardiac dysfuncton(in line with Ref. 58 and others [PMIDs: 26722126, 30837465, 35447412])? Assessing whether these metabolic changes contribute to the observed cardiac dysfunction would greatly strengthen the manuscript.

The authors speculate that enhanced systemic inflammation in females may account for the observed sex difference in cardiac dysfuncton(lines 136-137). However, there is no attempt to test this hypothesis. Overall, the susceptibility of male and female PWK/PhJ mice to systemic or vascular inflammation, as well as other pro-atherogenic processes, should be more thoroughly investigated, ideally in comparison to C57BL/6J mice.

Issues related to Figure 7: The scheme in Figure 7D is misleading, as it suggests an increase in glycine and alanine, whereas the data in Figure 7A clearly show a decrease in these amino acids. Considering that alanine, glycine, and phenylalanine were all decreased, the assumption that ceramide levels were increased from de novo ceramide synthesis using alternative amino acids (lines 286-288) is unlikely. A clearer explanation could be that these amino acids are utilized in ceramide production, which may account for their decrease and the corresponding increase in sphingolipids. Indeed, stable-isotope tracing studies in C57BL/6 mice showed that glycine-derived serine contributes to sphingolipid production in MASLD (PMID: 38171331). Thus, tracing studies are necessary to support the speculation regarding the link between the reduction of amino acids and de novo ceramide synthesis in PWK/PhJ mice.

Another speculation that lacks experimental support is that the upregulation of Sptlc3 in the liver may explain why PWK/PhJ is the most sensitive strain to MASH (lines 298-301). Targeting Sptlc3 specifically in livers of PWK/PhJ mice and other strains is necessary to support this hypothesis. In addition, what is the reason for the specific upregulation of the Sptlc3 subunit and not the other two subunits of SPT?

Lack of histopathological and immune cell phenotyping: Basic histological analysis of heart tissue (e.g., H&E, Picrosirius Red, etc.) was not provided. Additionally, the histopathological assessment of the liver, including the NAFLD activity score (NAS) and fibrosis score, was missing. Immunofluorescence analysis of immune cell subsets in both liver and heart tissues would also be valuable.

Data requiring further clarification: In Figure 2A, could the authors explain why females exhibit an increased liver-to-body weight ratio under RT in both CD- and WD-fed mice? In Supplementary Figure 1B, why females showed such high LDL levels on CD? In addition, the absolute AST levels are quite high in both males and females, even on CD. Why females

show a much higher increase in AST than males?

Manuscript title: Including "cardiac dysfunction" in the title would better align with the abstract and the data presented throughout the manuscript, potentially better highlighting the significance of the study.

Minor comments:

Data from metabolic cages (Promethion): It would be beneficial to include fat oxidation and glucose oxidation data. In addition, there are clear differences in RER in RT and TN during dark time in males versus females (Figure 1G), which were not sufficiently addressed.

Throughout the manuscript, MAFLD, MALFD (lines 82 and 111), NAFLD and NASH (line 214) are used instead of the recently accepted terminology - MASLD and MASH.

Figure 1F: Please add units for Y axis (Energy Expenditure)

Fig 5C: The absolute levels of all plasma measurements should be included, at a minimum, as Supplementary Material.

Lines 165-167: The authors speculate that “Genes related to the mevalonate pathway, crucial for cholesterol synthesis were downregulated, likely as a protective response to reduce lipotoxicity”. A more likely explanation would be negative feedback driven by SREBP2 suppression due to the overload of dietary cholesterol.

Version 1:

Reviewer comments:

Reviewer #1

(Remarks to the Author)
The author has made comprehensive revisions. However, the author should supplement the study with additional protein quantification experiments to further substantiate the fibrotic changes, such as measuring the protein levels of αSMA, COL1A1, COL1A3, CTGF, and TIMP1. These experiments should be added.

Reviewer #2

(Remarks to the Author)
The revisions have significantly strengthened the manuscript, and the authors have done a nice job in responding to the previous round of feedback.

Reviewer #3

(Remarks to the Author)
The authors have addressed most of the comments and acknowledged the key limitations of the study. The following issues remain and should be addressed:

1) The authors acknowledge that the absence of a direct comparison to other strains including C57BL/6J mice represents a key limitation of the current manuscript. Accordingly, because the study did not include analyses across other strains, statements suggesting superiority of the PWK/PhJ strain should be removed throughout the manuscript. Here are a couple of examples:
Abstract: “…male and female PWK/PhJ mice exhibit accelerated hepatic fibrosis and cardiac dysfunction…”
Conclusion: “…making them a preferred model to study the transition of MASLD to MASH”
Terms such as ‘accelerated’ or ‘preferred’ can only be used when conducting a comparative study.

2) Although measuring indices of systemic inflammation is relatively straightforward, the authors did not perform such assessments in the revised manuscript. Analyses of inflammatory gene expression in the liver would not address this limitation. Notably, speculations regarding systemic inflammation remained in the manuscript without any supporting data: “Larger liver size (Fig. 2A) and higher cholesterol levels (Fig. S1B) in females may contribute to systemic inflammation and impair heart function”.

3) Another statement that should be moderated, as it lacks experimental evidence including modulation of Sptlc3 in PWK/PhJ mice or other strains, is the following: “...the increased transcript levels of Sptlc3 and enhanced de novo synthesis are the primary drivers of ceramide and deoxy-ceramide accumulation in the liver, which could explain why PWK/PhJ is the most sensitive strain to MASH, developing fibrosis and mitochondrial dysfunction [21].”

4) The addition of liver histopathological analyses have strengthened the revised manuscript. However, the absence of histological and biochemical assessments to demonstrate cardiac dysfunction represents a major limitation, which weakens

the study’s conclusion regarding a dual model.

Version 2:

Reviewer comments:

Reviewer #1

(Remarks to the Author)
The revision has markedly strengthened the manuscript, and the authors have done a good work in responding to the previous round of feedback.

Reviewer #3

(Remarks to the Author)
The authors have addressed the concerns successfully, and this work will significantly advance the field.

Open Access This Peer Review File is licensed under a Creative Commons Attribution 4.0 International License, which permits use, sharing, adaptation, distribution and reproduction in any medium or format, as long as you give appropriate credit to the original author(s) and the source, provide a link to the Creative Commons license, and indicate if changes were made.
In cases where reviewers are anonymous, credit should be given to 'Anonymous Referee' and the source.
The images or other third party material in this Peer Review File are included in the article’s Creative Commons license, unless indicated otherwise in a credit line to the material. If material is not included in the article’s Creative Commons license and your intended use is not permitted by statutory regulation or exceeds the permitted use, you will need to obtain permission directly from the copyright holder.
To view a copy of this license, visit https://creativecommons.org/licenses/by/4.0/

POINT TO POINT REPLY

We sincerely appreciate the time and effort of all the Reviewers in evaluating our manuscript and for providing valuable feedback. Our responses to the Reviewers' comments are highlighted in blue, and the corresponding changes in the revised manuscript are also indicated in blue.

REVIEWER COMMENTS

Reviewer #1 (Remarks to the Author):

Although the design of this study is very systematic and meaningful data has been obtained, the hypothetical conclusions drawn lack validation and belong to descriptive articles. Based on this, I do not believe it has reached the level represented by the journal.

We agree that our study does not establish a direct mechanistic link between SPTLC3 and the liver dysfunction, as highlighted at the end of the manuscript. Fully addressing this would require additional lengthy animal experiments, which are beyond the scope of the present work and might shift focus away from our primary aim that is to establish a robust model to investigate the complex relationship between liver and heart disease. Importantly, we show that both males and females exhibit accelerated liver pathology together with impaired cardiac function following WD, demonstrating that this is a valuable model for investigating the links between these two interconnected complex diseases. We supported these findings with extensive *in vivo* phenotyping, and *ex vivo* analysis including plasma markers and liver histology along with a longitudinal study, transcriptomics analysis of liver and heart, comparison of mouse and human liver transcriptomics and targeted liver metabolomics. In the revised version, we have strengthened the work by adding proteomics analysis in liver and heart and targeted metabolomics in plasma and heart tissues.

While the proposed pathways remain hypothetical, they open a line of research for future mechanistic studies and highlight possible therapeutic targets. In the revised version, we have further strengthened this hypothesis by demonstrating that elevated sphingolipid species, recognized as atherosclerotic drivers and established CVD biomarkers, are present in the bloodstream and that the sphingolipid composition of cardiac tissue is also altered, thereby reinforcing the physiological relevance of our findings.

We are convinced that the systematic and integrative nature of this work is of value to the field, lays the necessary foundation for future mechanistic studies and aligns with the scope of the journal.

Reviewer #2 (Remarks to the Author):

In this work, López et al. explore the metabolic alterations in western diet-induced MASH. The authors found that amino acids and sphingolipids changed significantly in MASH and that there were some differences between genders. When performing metabolomics analysis, I think there are some problems,

especially the identification and quantification of metabolites. This is very critical and maybe influence experimental conclusions.

(1) The impact of housing temperature on diet-induced MASH. Why did the authors set only the room temperature group (RT, 24 °C) and the thermoelectric group (TN, 30 °C), and not the other temperature groups? The effect of temperature on metabolism is obvious. Too high or too low temperature will inevitably affect the metabolic states of the organism.

We focused on room temperature (RT) and thermoneutrality (TN) because these two conditions represent the most commonly used (RT) and physiologically relevant (TN) housing temperatures for mouse experiments.

RT reflects the standard housing conditions and was originally chosen for the comfort of human researchers, not for the physiological needs of mice [1]. Additionally, RT is easier to maintain and control in animal facilities than TN conditions, which require more precise regulation and often higher-cost management. However, RT imposes a mild cold stress, and this induces thermogenesis, a mechanism in which brown adipose tissue (BAT) generates heat [2]. This means rodents housed at RT are constantly maintaining elevated metabolic rates compared to TN housing conditions. This has been widely reported [3, 4], and we also demonstrated this difference with the indirect calorimetry data in **Figure. 1 F-G** and **Supplementary Figure 1A**.

In recent years, there has been growing concern about the impact of chronic thermal stress on metabolic outcomes, and housing temperature might partially explain why the findings in metabolic studies differ between humans and rodents [5-7].

TN, in contrast, represents the temperature at which animals do not need to expend extra energy to maintain body temperature; essentially the “natural” temperature of metabolic homeostasis, for *Mus musculus* is 30–32 °C [3, 4]. In the context of metabolic diseases, TN housing has been reported to exacerbates MASLD/MASH disease [3, 8, 9].

Studies conducted in normal-weight and DIO (diet-induced obese) C57BL/6J mice, testing 22, 25, 27.5, and 30 °C conclusively demonstrate mice still experience cold-induced thermogenesis at 27.5°C, and the optimal thermoneutrality in the mouse is 30 °C [10]. Including additional intermediate temperatures between RT and TN would add experimental complexity and raise ethical considerations without necessarily yielding further mechanistic insight relevant to the questions addressed in this study.

Did the authors take into account the effect of temperature in their metabolic assays for amino acids and sphingolipids.

We performed an *in vivo* panel of metabolic phenotyping and some *ex vivo* analyses, including histology, transcriptomics and plasma markers, to compare the overall metabolic status at the two temperatures. However, we did not assess metabolomics under different temperature conditions due to the high cost of targeted metabolomics and because TN represents the physiologically relevant temperature for metabolic studies. Hence, we also conducted our longitudinal and subsequent analyses at TN to ensure biological relevance.

Based on our transcript profiling, we expect similar changes in hepatic amino acids and sphingolipids levels under RT. Transcriptomic data shows an increase in *Sptlc3* and *Smpd3* expression, which likely reflects the synthesis of ceramides from *de novo* synthesis and the increased sphingomyelin degradation, independent of the housing temperature (**Supplementary Figure 3D**).

(2) The author conducted absolute quantitative analysis on both sphingolipids and amino acids, but I did not find information on standard curves and quality control samples, as well as specific quantitative data, correlation coefficients, etc. from the raw data provided by the author (https://lisp-lms.shinyapps.io/PWK_study_app/). What isotopic internal standards did the author use?

Yes, we performed targeted metabolomics analysis to obtain absolute quantification of metabolites NAD$^+$ metabolome, sphingolipids, and amino acids. Details, such as standard curves, quality control samples, quantitative data, correlation coefficients, and other technical parameters, are now provided in the Materials and Methods. These analyses were performed by Prof. Julijana Ivanisevic at the Metabolomics Unit of the University of Lausanne (UNIL), and further information is provided in the following two methodological manuscripts:

For the NAD$^+$ metabolome - Van der Velpen V et al. [11]. Table S1 and S2, S4 and S5.

For amino acids - Teav T et al. [12]. Table S2 and S4.

For sphingolipid metabolites, this information was not previously published. Therefore, we have now provided the relevant information in the revised version of the manuscript as a **Supplementary Excel table 3**. All the data about the sphingolipid MRM method, standards and associated internal standards, calibration range (including coefficients of determination) and method precision are now provided as:

Table 3.1: Names of standards and associated internal standards (isotopically labelled).

Table 3.2: Standard concentrations across all calibrators used for calibration curves, and the correlation coefficients of those curves.

Table 3.3: Coefficients of variation (CVs) across quality control samples (pooled samples representative of the entire sample batch) for each species.

(3) Figure 7B. The authors accurately quantified the contents and variations of different sphingolipid species, and the authors even made judgments about the composition of fatty acid side chains and cis-trans isomers. How did the authors make the identification? MS/MS analysis was performed? How were the cis-trans isomers determined?

We would like to thank the Reviewer for pointing this out, and we apologize for the confusion. We did not try to make any judgments about the composition of fatty acid side chains or cis/trans isomers. Some lipid names in the figures contained notations such as "Z," "R" or "t" which indicate stereochemistry or double-bond configuration. These names with specifications of unsaturated positions and stereoisomers from the standard compounds used during method development remained in the manuscript. The standards themselves are pure and synthetically defined, and their names therefore carry this level of specificity. However, as correctly pointed out, our experimental methods do not allow us to distinguish between different stereoisomers (e.g., trans or cis; Z or R forms) nor determine the positions of double bonds. The names of the measured species have therefore been corrected accordingly throughout the figures.

(4) Chronic WD challenge impacts amino acid and sphingolipid metabolism. The authors performed metabolomics analysis. Non-targeted or targeted metabolomics analysis? If it is a non-targeted metabolomics analysis, how did the author select amino acids as differential variables from thousands of variables? If

it was a targeted analysis, did the authors determine that the amino acids and sphingolipids would change before the experiment began?

Our study employed a targeted metabolomics approach and have revised the Material and Methods to explicitly state this in the titles. The rationale for focusing on targeted amino acid and sphingolipid analysis were based on our transcriptomic data, which indicated that these metabolites could be altered. See also answer to Question #2.

(5) In this manuscript, the authors identified abnormalities in the metabolism of amino acids and sphingolipid, but further validation is needed to determine whether the metabolic abnormalities are related to MASLD and MASH, and whether the amino acids and sphingolipids are the cause of MASLD and MASH or merely a phenotype of altered metabolism in MASLD and MASH.

While our study identifies altered liver amino acid profiles associated with MASH, it does not establish whether these changes are causative or consequential. Clarifying this will require further mechanistic studies beyond the scope of the current work. We have acknowledged these limitations in the revised manuscript (**lines 472–476**).

However, we would like to point that both MASH and CVD are complex metabolic diseases in which no single factor acts as the sole driver [13, 14]. Instead, key contributors, such as amino acids and sphingolipids, function as both causes and consequences, creating positive feedback loop fueling the pathogenesis of MASH [15-17].

(6) How does cholesterol change in the liver? The authors only examined its changes in plasma, and if the authors had performed a metabolomics study, they should have been able to obtain information on how cholesterol changes in the liver. Because, elevated liver cholesterol levels are common in MASH patients. In addition, changes in amino acids and sphingolipids in plasma should be of interest.

Our study employed targeted metabolomics focused on liver sphingolipids, amino acids, and NAD^+ metabolites. This approach does not provide direct information on hepatic cholesterol levels. However, we have now measured total cholesterol in the liver, as well as triglycerides and cholesterol levels specifically in liver lipid droplets. We have updated our manuscript and included this in the **Supplementary Fig. 7C-D**. In MASH, total liver cholesterol does not necessarily increase, instead, it increases specifically in hepatocyte lipid droplets [18]. We observed that, although total hepatic cholesterol levels remained unchanged, cholesterol and triglyceride content in lipid droplets increased from the start of the WD and remained consistently elevated across all time points (**Supporting Figure 1**).

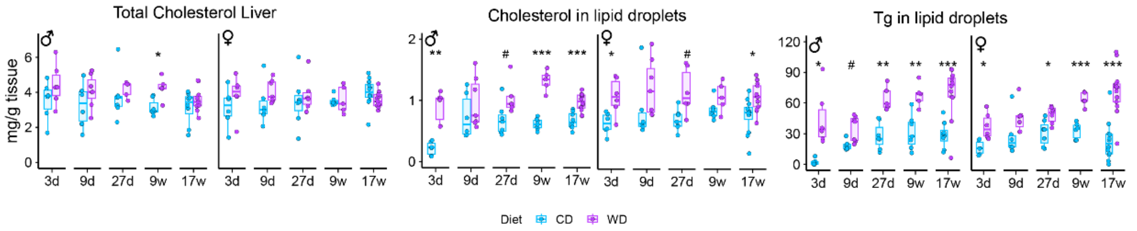

Supporting Figure 1. *Quantification of total cholesterol in liver tissue, and cholesterol and triglycerides in liver lipid droplets. Statistical analysis was conducted using a two-sided Student's t-test with Benjamini–Hochberg adjusted p values.* $^{\#}P < 0.1$, $^{*}P < 0.05$, $^{**}P < 0.01$, $^{***}P < 0.001$.

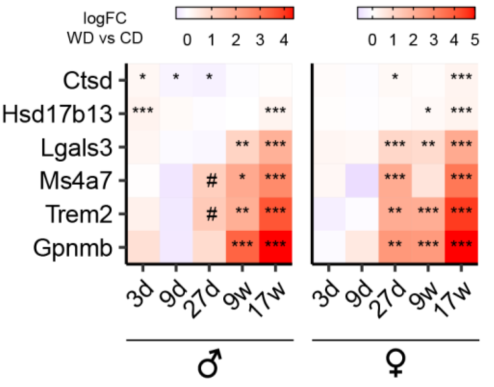

Supporting Figure 2. *WD vs CD log2 fold changes in RNA expression of genes associated with MASH. Macrophage-associated genes: Gpnmb, Trem2, and Lgals3. Lipid droplets-associated genes: Hsd17b13 and Ctsd. Statistical analysis was conducted using a two-sided Student's t-test with Benjamini–Hochberg adjusted p values. #P < 0.1, *P < 0.05, **P < 0.01, ***P < 0.001*

These findings align with a recent study [18] and have important implications, as cholesterol within lipid droplets represents a pool of esterified and free cholesterol that can directly impair organelle function and disrupt cellular homeostasis. Free cholesterol in lipid droplets can destabilize their membranes, leak into organelles, and activate macrophages and hepatic stellate cells, key drivers of inflammation and fibrogenesis in MASH [19]. From our transcriptomic data, we observed that 27 days of WD feeding induced expression of MASH-associated macrophage genes, *Gpnmb, Trem2, Ms4a7, and Lgals3,* which are linked to inflammation and immune cell infiltration; meanwhile genes related to lipid droplet turnover, *Hsd17b13* and *Ctsd* are induced at 17 weeks of WD, which are related to lipid metabolism dysfunction and fibrosis (**Supporting Figure 2**).

Since lower ratios of phosphatidylcholine (PC) to phosphatidylethanolamine (PE) can compromise membrane integrity and contribute to liver damage, we also measured this ratio in liver lipid droplets in a small number of liver samples. Even with a limited number of animals analysed, we observed a decrease in the PC/PE ratio at 9 and 17 weeks of diet in males and at 17 weeks in females (**Supporting Figure 3**).

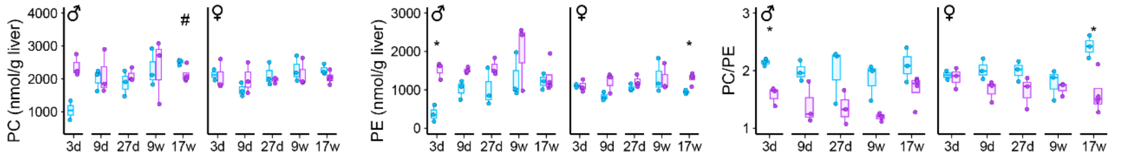

Supporting Figure 3. *Phosphatidylcholine (PC), phosphatidylethanolamine (PE) and PC/PE ratio in liver lipid droplets*

In summary, our results align with the recent study by Sakuma I. et al. [18], emphasizing that, rather than total hepatic cholesterol, accumulation of cholesterol in the liver lipid droplets acts as a key driver of inflammation and fibrosis in MASH.

We also appreciate the Reviewer's suggestion regarding additional metabolomic analysis in plasma and agree that examining amino acid and sphingolipids would be valuable for our study; this has now been included into **Figure 8D** and **Supplementary Figure 10D**, and the corresponding text (**lines 370–383**) has been updated.

(7) Was absolute quantification also performed for the organic acids in Figure 7A? How this data was obtained is not explained by the authors.

All metabolomics analyses performed in this study were targeted analyses, and absolute quantification of organic acids were included in the amino acid and NAD^+ metabolome. The measurements included in each analysis are the following:

<u>NAD^+ metabolome analysis:</u> tryptophan, nicotinic acid mononucleotide (NaMN), nicotinate ribonucleotide, kynurenine, nicotinic acid, adenine dinucleotide (NaAD), kynurenic acid, NAD^+, anthranilic acid, nicotinamide mononucleotide (NMN,

nicotinamide ribotide), 3-hydroxy-kynurenine, nicotinamide riboside (NR, ribosyl-nicotinamide), xanthurenic acid, nicotinamide (NAM, niacin, Vitamin B3), 3-hydroxyanthranilic acid, picolinic acid, quinolinic acid, nicotinic acid.

Amino acid analysis: alanine, glutamate, ornithine, alpha-aminoadipate, glutamine, phenylalanine, alpha-aminobutyrate, glycine, pipecolate, anserine, guanidinoacetate, proline, arginine, histidine, serine, asparagine, homocitrulline, taurine, beta-alanine/sarcosine, isoleucine, threonine, carnosine, kynurenine, 4-hydroxy-proline, citrulline, leucine, tryptophan, creatine, lysine, tyrosine, gamma-aminobutyrate, methionine, valine.

This information is available in Van der Velpen V et al. [11] and Teav T et al. [12], referenced in the Material and Methods.

Reviewer #3 (Remarks to the Author):

Rodríguez-López et al. report their comprehensive investigation into the cardiometabolic phenotypes of male and female PWK/PhJ mice housed at room temperature (RT) or thermoneutrality (TN) for up to 17 weeks, focusing on the development of metabolic dysfunction-associated steatohepatitis (MASH) and cardiac dysfunction. Given that cardiovascular disease (CVD) is the leading cause of death in patients with MASH, and there is a lack of mouse models that accurately replicate both conditions, this study highlighting Western diet (WD)-fed PWK/PhJ mice as a model suitable for the study of cardiac dysfunction in MASH is both important and significant. Strengths of this study include comprehensive analyses of livers, plasma, cardiac function, systemic energy metabolism, and body composition, with comparisons based on sex and housing temperature. The comparison of mouse and human liver transcriptomics and the metabolomics studies highlighting dysregulated amino acid and sphingolipid metabolism are additional strengths. The results of the longitudinal studies, demonstrating a strong MASH-fibrosis phenotype along with cardiac dysfunction after just 17 weeks on the WD, especially in females, could be highly valuable to the field, as current models typically require longer feeding periods. Despite these strengths, the study has several limitations that warrant further attention and investigation as outlined below.

Comparisons to male and female C57BL/6J mice is necessary to support key claims: In their recent study (PMID: 36787127), the authors reported that the PWK/PhJ strain is highly susceptible to WD-induced MASH compared to other mouse strains, including the widely used C57BL/6J. That study included only male mice and did not examine cardiac function. In the current study, both male and female PWK/PhJ mice were included, and both MASH and cardiac functions were assessed. However, comparison to the commonly used C57BL/6J mouse strain was lacking. While most studies using C57BL/6J mice report more severe MASH in males, recent research has shown an opposite outcome (PMID: 39911943). Moreover, sex-specific differences in MASH-associated cardiac function in various mouse strains have not been studied. Thus, endpoint analyses should be compared between male and female PWK/PhJ mice and C57BL/6J mice to support the authors' claim that MASH-fibrosis and cardiac dysfunction are accelerated in PWK/PhJ mice.

In a previous study, we examined the effects of HFD at RT on cardio-metabolic traits in both male and female mice of nine different strains, including PWK/PhJ and C57BL/6J [20]. Parameters such as body weight, OGTT, and indirect calorimetry were assessed *in vivo*, while plasma biochemistry and liver RNA-seq were analyzed *ex vivo*. The results indicated that female PWK/PhJ mice exhibited the most pronounced alterations in blood biochemistry and the most comprehensive response to HFD, including the strongest immune activation in the liver at RNA levels. From these findings, we concluded that the PWK/PhJ strain may develop more severe metabolic defects in response to HFD than the commonly used "reference strain", C57BL/6J. To test this hypothesis further, we conducted a new study to provide an in-depth characterization of MASH susceptibility in these strains [21], excluding those strains that develop obesity and diabetes (NOD/ShiLtJ, and NZO/HILtJ) and including only males, as incorporating sex as a variable would have added complexity and limited the depth of the characterization due to time and resource constraints. We also considered the recent concerns regarding housing temperature in metabolic studies. Our present study is a follow-up of these two studies, considering four variables: housing temperature, sex, diet and time-dependent disease signatures. Adding another strain for comparison would have increased not only the size of the experiment but also the complexity of the analysis. We agree with the Reviewer that, without a direct comparison of cardiac function between PWK/PhJ and C57BL/6J, we cannot conclude that PWK/PhJ develops a worse cardiac phenotype than C57BL/6J. Nonetheless, our previous work indicates that liver dysfunction is more severe in PWK/PhJ compared to C57BL/6J. We also acknowledge that PWK/PhJ females have not been directly compared between C57BL/6J females under WD and TN, and agree that such a comparison could be of interest and we have now included this as a limitation of our current study (**lines 469–472**). Repeating these experiments to include the comparison with C57BL/6J would require substantial additional time and resources. Moreover, increasing animal numbers would raise ethical concerns that cannot be justified for exploratory purposes alone, at least in Switzerland. Importantly, the extensive published literature on C57BL/6J, already provides consistent evidence supporting the C57BL/6J phenotype during MASH and CVD development in different environmental conditions.

We appreciate the Reviewer's comment regarding the recent study Arivazhagan L et al. [22] showing that C57BL/6J females may develop a more severe phenotype compared to males when subjected to specific dietary conditions. While both our study and the cited study seek to model MASH in mice, it is important to note that the composition and translational relevance of the diet differ significantly, limiting direct comparison of the results.

The FPC (Fructose-Palmitate-Cholesterol) diet combines fructose, *trans* fat, palmitic acid and cholesterol (1.25%). In addition, the drinking water of the MASH cohort was supplemented with fructose (23.1g/L) and glucose (18.0g/L), causing rapid and pronounced steatosis, inflammation, and fibrosis. Although this is an aggressive model to study MASH, it is less representative of the average human diet. The Western diet we used contains fats and sugars in proportions closer to the human Western diet. Its effects on fibrosis are more gradual, reflecting the natural progression of the disease in humans.

While it is true that most studies do not report robust MASH induction in C57BL/6J females, it is interesting that this study shows the opposite pattern. However, the limited fibrosis observed in males could represent a potential limitation of the model. Our findings that PWK/PhJ strain is highly susceptible to WD-induced MASH in both males

and females does not contradict the general outcomes reported in the study cited by the Reviewer, rather, they likely reflect differences in the models and the diets employed.

We have discussed this study (**lines 395–398**).

The manuscript offers limited insight into the mechanisms linking MASH and CVD: The authors report cardiac dysfunction in WD-fed PWK/PhJ mice, with notably more severe effects in females. However, RNA-seq of the hearts showed minimal changes. Moreover, metabolomics highlighting dysregulated amino acid and sphingolipid metabolism was performed only on livers with no attempt to link the findings to cardiac function. Are similar metabolic changes observed in plasma and the heart tissue? Are VLDL particles of WD-fed PWK/PhJ mice contain higher ceramides (in line with Ref. 50)? Alternatively, are changes in amino acids promote cardiac dysfuncton (in line with Ref. 58 and others [PMIDs: 26722126, 30837465, 35447412])? Assessing whether these metabolic changes contribute to the observed cardiac dysfunction would greatly strengthen the manuscript.

Following the Reviewer's suggestion we have now performed metabolomic measurements in total plasma and heart. The data show elevated levels of ceramides and sphingomyelins in WD plasma, which is consistent with both our findings in liver and with previous studies showing that these lipid species are implicated in the development of atherosclerosis [23-25]. Ceramides levels were also elevated in heart. Although the increase was more modest than in liver and plasma, the elevation of ceramides is noteworthy, especially considering the minimal changes detected in the transcriptomics and proteomics analyses of the heart. These data have now been incorporated into **Figure 8D** and **Supplementary Figure 10D**, and the corresponding text (**lines 370–383**) has been updated.

This approach does, however, not allow us to determine whether the observed increase in plasma ceramides and sphingomyelins is specifically associated with VLDL or with other lipoprotein fractions. We unfortunately do not have sufficient plasma available to isolate VLDL fractions for this type of analysis.

The authors speculate that enhanced systemic inflammation in females may account for the observed sex difference in cardiac dysfuncton (lines 136-137). However, there is no attempt to test this hypothesis. Overall, the susceptibility of male and female PWK/PhJ mice to systemic or vascular inflammation, as well as other pro-atherogenic processes, should be more thoroughly investigated, ideally in comparison to C57BL/6J mice.

As previously discussed, a comparison of PWK/PhJ with C57BL/6J was not the aim of our work. Therefore, we are unable to provide this analysis.

Detecting circulating pro-inflammatory cytokines and chemokines in plasma during systemic inflammation is challenging, as their concentrations are often below the detection limit, which is a known limitation [26]. However, these molecules primarily exert their effect locally at sites of inflammation. Accordingly, we compared changes in liver inflammatory gene expression induced by WD and assessed differences between sexes. Inflammatory genes were significantly upregulated by WD and were higher in females than in males (**Supporting Figure 4 A & B).** Proteomics also showed that females have higher hepatic levels of C3, FGA, FGB and FGG than males, reflecting a molecular signature of increased inflammatory stress in the liver (**Supporting Figure 4 C)**. Elevated hepatic ceramide levels are also associated with the induction of pro-inflammatory processes in hepatocytes, contributing to the amplification of local

inflammation and fibrosis. In our comparison between sexes, we found that female mice exhibited higher hepatic ceramide levels than males. (**Supporting Figure 4 D).** Sex differences in hepatic ceramide levels have been reported in other strains, including under chow diet. These differences are partly attributed to testosterone in males, which suppresses the expression of ceramide synthase enzymes [27].

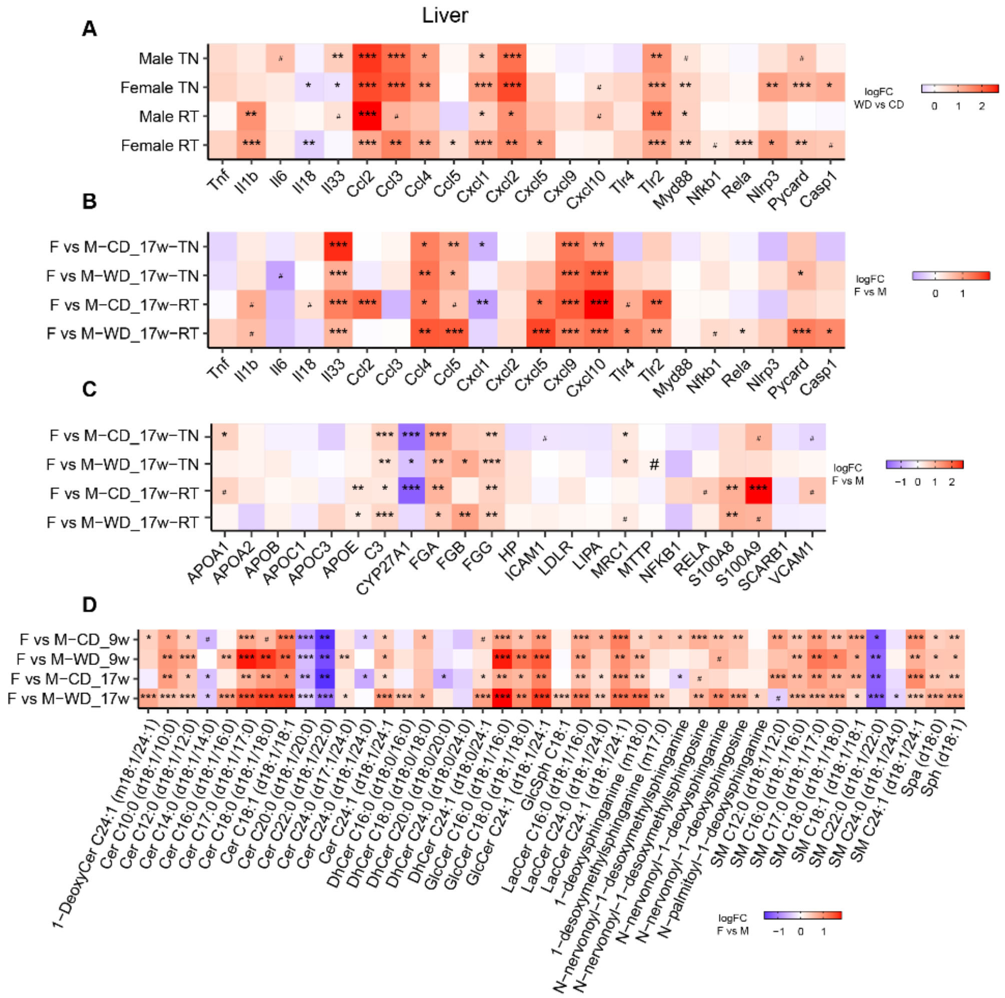

Supporting Figure 4. ***(A)*** *WD versus CD log2 fold changes of inflammatory genes in liver.* ***(B)*** *Females versus males log2 fold changes of inflammatory genes in liver.* ***(C)*** *Females versus males Log2 fold changes for liver proteins.* ***(D)*** *Females versus males Log2 fold changes for sphingolipid species in liver tissue. Statistical analysis was conducted using a two-sided Student's t-test with Benjamini–Hochberg adjusted p values. #P < 0.1, *P < 0.05, **P < 0.01, ***P < 0.001.*

Regarding pro-atherogenic processes, elevated levels of plasma cholesterol and lipoproteins, particularly LDL, are directly associated with an increased risk of developing atherosclerosis. In our plasma analysis comparing females and males, we demonstrated that females showed higher LDL levels than males (**Supporting Figure 5 A**). Additionally, circulating sphingolipids, including ceramides — which are primarily synthesized by the liver — have been increasingly recognized as plasma biomarkers for CVD and the development of atherosclerosis. Sex comparisons of plasma sphingolipids confirmed that female mice exhibit higher levels than males (**Supporting Figure 5 B)**, including Cer(d18:1/16:0) and SM(d18:1/16:0), both well-recognized markers of atherosclerosis [23-25]. Finally, sex comparison of heart tissue revealed differences in sphingolipid profiles, which reflect heart remodelling and dysfunction (**Supporting Figure 5 C)**. Thus, all our data support the hypothesis that enhanced systemic

inflammation in females accounts for the distinct heart phenotypes observed between sexes.

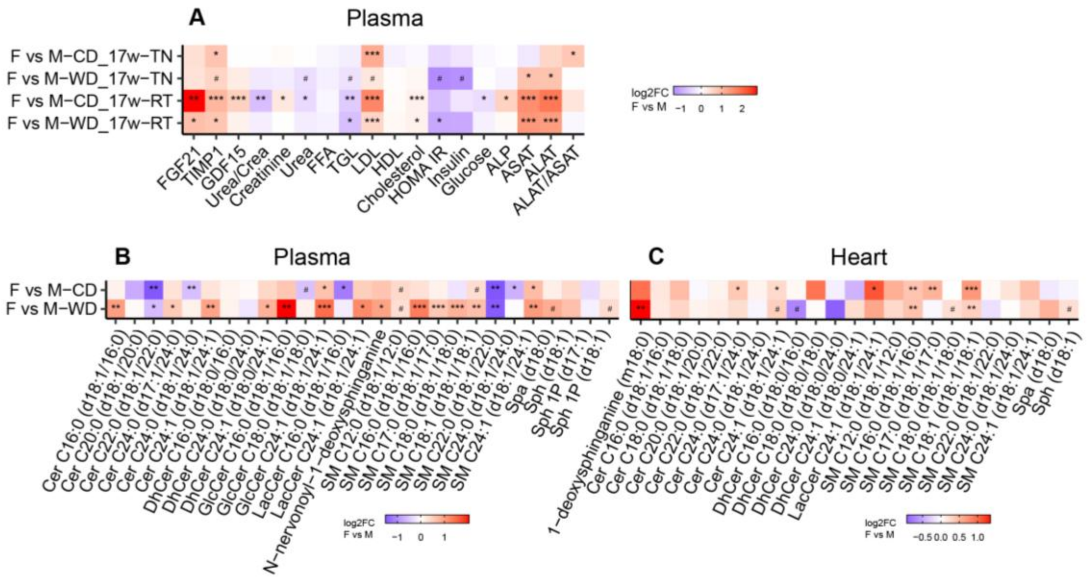

Supporting Figure 5. (A) *Log2 fold changes for plasma analytes.* ***(B)*** *Log2 fold changes for sphingolipid species in plasma.* ***(C)*** *Log2 fold changes for sphingolipid species in heart tissue. Statistical analysis was conducted using a two-sided Student's t-test with Benjamini–Hochberg adjusted p values. #P < 0.1, *P < 0.05, **P < 0.01, ***P < 0.001*

Similar graphics as shown in **Supporting Figure 4 and 5** can be generated through the online App (https://lisp-lms.shinyapps.io/PWK_study_app/), through which all our data can be queried.

Issues related to Figure 7: The scheme in Figure 7D is misleading, as it suggests an increase in glycine and alanine, whereas the data in Figure 7A clearly show a decrease in these amino acids. Considering that alanine, glycine, and phenylalanine were all decreased, the assumption that ceramide levels were increased from de novo ceramide synthesis using alternative amino acids (lines 286-288) is unlikely. A clearer explanation could be that these amino acids are utilized in ceramide production, which may account for their decrease and the corresponding increase in sphingolipids. Indeed, stable-isotope tracing studies in C57BL/6 mice showed that glycine-derived serine contributes to sphingolipid production in MASLD (PMID: 38171331). Thus, tracing studies are necessary to support the speculation regarding the link between the reduction of amino acids and de novo ceramide synthesis in PWK/PhJ mice.

We thank the Reviewer for these comments.

Figure 7D (**now Figure 8E**) has been updated to reflect the decrease in these amino acids, consistent with the color code in the figure legend. See also **lines 348–353**. Lines **434–435** have also been updated to include the cited study.

We agree with the Reviewer that tracing studies would be necessary to definitively support the link between the reduction of amino acids and *de novo* ceramides synthesis in PWK/PhJ mice. However, our laboratory does not specialize in these types of experiments, and they require substantial time and resources and a repeat of the *in vivo* work, which is beyond the scope of the current study. We have therefore discussed this point as a limitation in the revised manuscript (**lines 472–476**).

Another speculation that lacks experimental support is that the upregulation of Sptlc3 in the liver may explain why PWK/PhJ is the most sensitive strain to MASH (lines 298-301). Targeting Sptlc3 specifically in livers of PWK/PhJ mice and other strains is necessary to support this hypothesis. In addition, what is the reason for the specific upregulation of the Sptlc3 subunit and not the other two subunits of SPT?

We agree that targeting *Sptlc3* specifically in the livers of PWK/PhJ mice would be necessary to validate the hypothesis that *Sptlc3* induction is the reason why PWK/PhJ mice are most sensitive to MASH induction through the diet. We plan to explore this in future studies, including testing SPTLC3-targeted compounds as potential therapeutic interventions for MASH and CVD, but feel this is beyond the scope of the current study. We have included this point as a limitation in the revised manuscript (**lines 476–480**).

Although SPTLC1 and SPTLC2 constitute the main catalytic core for basal SPT activity, SPTLC3 is inducible and its expression increases under metabolic conditions. Additionally, SPTLC3 differs from SPTLC1 and SPTLC2 in the generation of non-canonical sphingolipids with shorter (16-carbon) sphingoid bases [28]. However, the biological roles of SPTLC3 remain incompletely understood. SPTLC3 can regulate mitochondrial function [29], plasma membrane signalling, and gluconeogenesis [30]. In our data, PWK/PhJ was the only strain showing mitochondrial dysfunction after 17 weeks of WD. Since SPTLC3 is critical for mitochondrial complex I function [29], it may play a key role in regulating mitochondrial function specifically in PWK/PhJ strain. Additionally, a recent study showed that the farnesoid X receptor (FXR) regulated the expression of SPTLC3. Reduced FXR activity, as occurs in metabolic liver disease, relieves repression of SPTLC3, leading to increased SPTLC3 expression and altered sphingolipid metabolism [31]. Genes and proteins associated with bile acid metabolism (**Supporting Figure 6 A & B**) and mitochondrial function (**Supporting Figure 6 C**) are downregulated in PWK/PhJ animals. Hence, SPTLC3 may modulate multiple processes in MASH and additional studies are required to better understand its function and that of its metabolites.

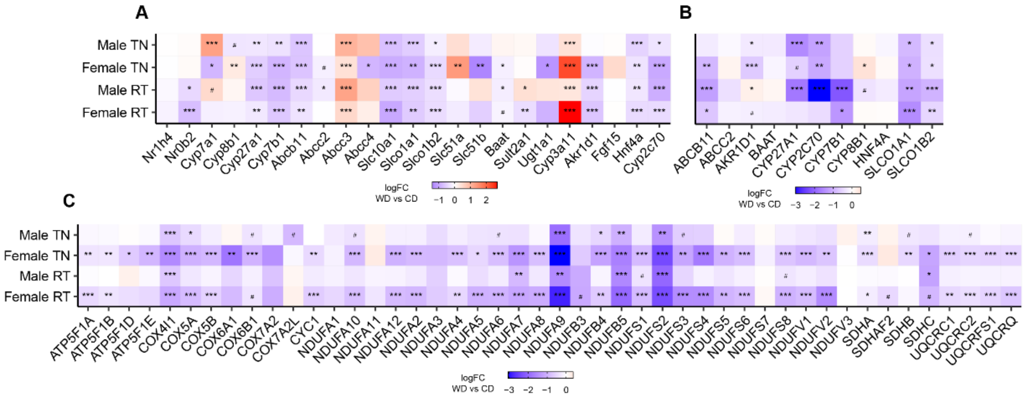

*Supporting Figure 6. (A) WD versus CD log2 fold changes for liver gene expression associated with bile acid metabolism. (B) WD versus CD log2 fold changes for liver protein expression associated with bile acid metabolism. (C) WD versus CD log2 fold changes of liver mitochondria protein expression. Statistical analysis was conducted using a two-sided Student's t-test with Benjamini–Hochberg adjusted p values. #P < 0.1, *P < 0.05, **P < 0.01, ***P < 0.001*

Lack of histopathological and immune cell phenotyping: Basic histological analysis of heart tissue (e.g., H&E, Picrosirius Red, etc.) was not provided. Additionally, the histopathological assessment of the liver, including the NAFLD activity score (NAS) and fibrosis score, was missing. Immunofluorescence

analysis of immune cell subsets in both liver and heart tissues would also be valuable.

Due to the small size of the hearts, we collected tissue exclusively for molecular analyses and we do not have material for histology. As histological analysis was not possible, we conducted proteomic profiling to gain deeper insights into molecular changes in the heart. Similar to the transcriptomic data, only modest differences were observed between groups (**Supplementary Figure 5A-D**). We believe this is likely because the analyses were performed on whole heart rather than isolated left ventricular samples, as we discussed in the manuscript. While histological data are not available, echocardiography (**Figure 2E-F**) clearly demonstrates ventricular hypertrophy, supporting structural remodelling in our model. We also performed targeted sphingolipid analysis **(Figure 8D)**, which revealed cardiac lipid alterations and elevated circulating ceramides and sphingomyelin species in plasma **(Figure 8D)**, commonly associated with atherosclerosis. Along with the previously presented biochemical data (**Figure 1I, Supplementary Figure 1B, Figure 6C**), these findings provide evidence of cardiac dysfunction and indicate an increased risk for cardiovascular events.

Regarding the histopathological assessment of the liver, we initially analysed tissue sections showing % macrosteatosis per tissue area from H&E samples (**Supplementary Figure 2C, Supplementary Figure 8C**), % positive cells to assess inflammation from CD45 slides (**Supplementary Figure 8C**), and % fibrosis per tissue area from SR slides (**Figure 2C, Supplementary Figure 8C**). We selected this approach because it does not require the intervention of an expert pathologist and provides a quantitative analysis of the liver slides. However, we agree with the reviewer regarding the importance of the NAFLD Activity Score (NAS) and fibrosis score. Therefore, in the revised manuscript, expert pathologists evaluated the H&E and SR slides and the results are now incorporated in **Supplementary Figure 2D** and **Figure 6E**. Individual NAS and fibrosis scores for all mice are now available in an Excel file as **Supplementary Table 2**. We also appreciate the Reviewer's suggestion regarding immunofluorescence analysis of immune cells. We now provided α-SMA evaluation in liver tissue at 9 and 17 weeks of challenge to demonstrate hepatic stellate cell activation (**Supplementary Figure 8D**).

Data requiring further clarification: In Figure 2A, could the authors explain why females exhibit an increased liver-to-body weight ratio under RT in both CD- and WD-fed mice? In Supplementary Figure 1B, why females showed such high LDL levels on CD? In addition, the absolute AST levels are quite high in both males and females, even on CD. Why females show a much higher increase in AST than males?

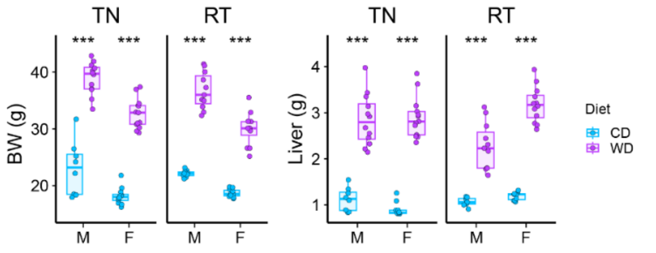

Supporting Figure 7. Body and liver weights (g) measured at sacrifice. *Statistical analysis was conducted using a two-sided Student's t-test with Benjamini–Hochberg adjusted p values. ***P < 0.001.*

In both CD and WD– RT mice, females exhibit a higher liver-to-body weight ratio primarily because their body weights are significantly lower than males, while their absolute liver weights are higher (**Supporting Figure 7)**. This leads to a proportionally larger liver relative to body size.

The exact mechanism explaining why PWK/PhJ females have high LDL plasma levels is not fully understood. A previous study performed by Price TR et al. [32], already reported that PWK/PhJ females have

increased concentrations of several LDL subclasses, as well as increased HDL and VLDL levels compared to males. In this study the authors identified splice and untranslated region variants in the *Gpam* gene in the PWK/PhJ strain. A specific variant in GPAM (rs10787429) has been associated with increased susceptibility to liver injury and elevated circulating lipid levels, including total, LDL, and HDL cholesterol levels in humans [33].

The plasma ALT and AST levels are strain-, diet-, and sex-specific, having different baseline levels and responses to diet challenge across genetic backgrounds and between males and females. Our previous study demonstrated the strain-specificity of these plasma markers, with PWK/PhJ being the strain with higher levels after WD (**Supporting Figure 8**). Sex differences have been also reported in different MASLD/MASH models [34-36]. In the longitudinal study by Spooner MH et al. [34], plasma levels of ALT and AST increased with WD in females, reaching levels even higher than those observed in males. The high increase in AST observed in female PWK/PhJ could be related to differences in lipid metabolism. As presented in **Supplementary Figure 1B**, females showed higher plasma LDL levels than males, even under CD. AST levels are also positively associated with liver fibrosis. In the semi-quantitative analysis of liver fibrosis score (**Supplementary Figure 2D**), females at RT scored higher fibrosis levels under both CD and WD than males, which could partially explain the elevated AST levels in females under RT. Hormone-mediated responses could have contributed to these outcomes, however, hormone levels were not measured in our animals.

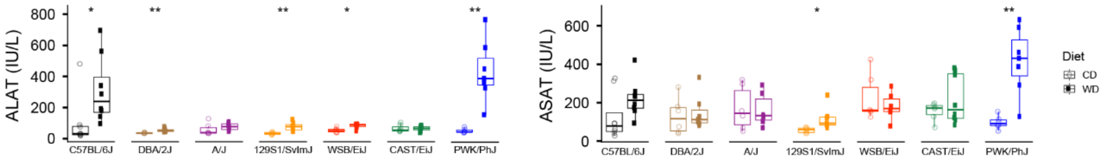

Supporting Figure 8. *Plasma ALAT and ASAT levels from different mouse genetic backgrounds from Benegiamo et al. 2023.*

Manuscript title: Including "cardiac dysfunction" in the title would better align with the abstract and the data presented throughout the manuscript, potentially better highlighting the significance of the study.

We have now updated the manuscript title.

Minor comments:

Data from metabolic cages (Promethion): It would be beneficial to include fat oxidation and glucose oxidation data. In addition, there are clear differences in RER in RT and TN during dark time in males versus females (Figure 1G), which were not sufficiently addressed.

We have not previously computed fat/glucose oxidation in our Promethion data. For this analysis, we applied the equations developed by Frayn KN [37] for glucose and fat oxidation, keeping units in mL/min for mice. We present the results both as absolute values and normalized to body weight (**Supporting Figure 9**). The requested plot was not included in the main manuscript but is now available in our online app (https://lisp-lms.shinyapps.io/PWK_study_app/), where it can be viewed and downloaded.

Regarding the RER differences, due to space limitations, we were unable to present all the data in as much detail as we would have liked. In the first part of the manuscript

(temperature effect on the cardiometabolic status of PWK/PhJ mice), we are taking in account three factors: temperature, diet and sex and all influence the metabolic parameters.

RER indicates which substrate is being oxidized, higher values reflect increased carbohydrate oxidation, whereas lower values reflect increased fat oxidation [37, 38]. At RT mice are below their thermoneutral zone, so they require extra heat to maintain body temperature. To generate this heat, they rely more on carbohydrate oxidation (**Supporting Figure 9**), as carbohydrates provide energy more quickly than fat. In non-shivering thermogenesis animals, carbohydrates are preferentially oxidized, mainly by the BAT [2]. At TN, mice require minimal thermogenesis, so energy comes from fat oxidation (**Supporting Figure 9**), in consequence the glucose oxidation is lower compared to RT. This explains why glucose oxidation is higher at RT than in TN, which aligns with the observed higher RER at RT, indicating greater carbohydrate utilization.

Additionally, diet influences RER. At RT mice on CD (D16042904B: 10% kcal from fat, 17% kcal from protein, 73% kcal from carbohydrates) have more carbohydrate available compared to mice on a WD (D12079B: 40% kcal from fat, 17% kcal from protein, 43% kcal from carbohydrates), resulting in higher RER compared to their counterparts at WD. At TN, since thermogenic demands are low, both CD and WD mice rely more on fat oxidation, and the RER difference between diets is reduced.

Sex also influences RER. At RT, males have higher lean mass (**Figure 1H**) than females, resulting in a higher metabolic rate and more reliance on carbohydrate oxidation (**Supporting Figure 9**) to meet their energy demands. Consequently, RER is higher in males compared to females at RT. At TN, thermogenic demand is low, and in consequence the sex difference in RER is also lower.

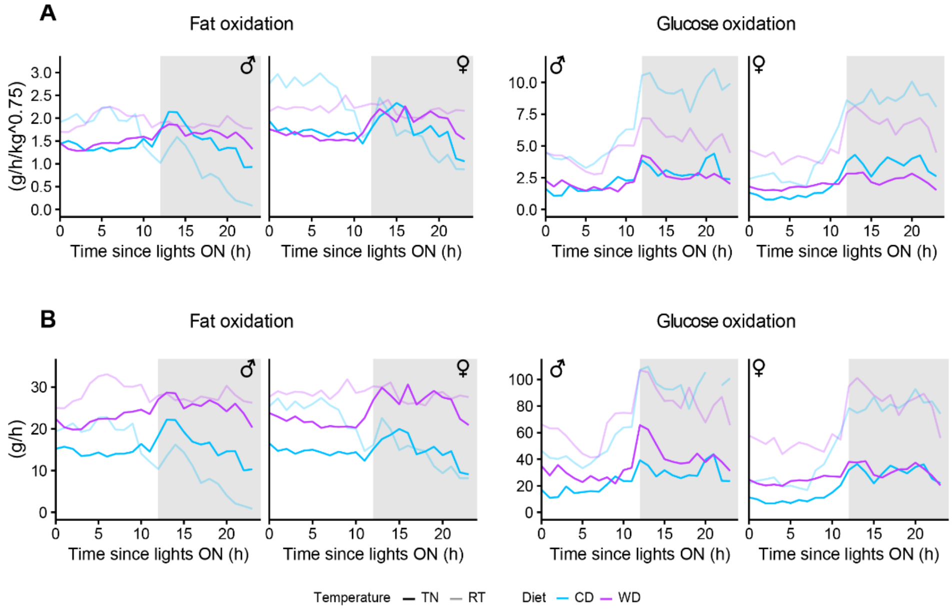

***Supporting Figure 9. (A)** Fat and glucose oxidation normalized by body weight (g/h/kg^0.75) and **(B)** non-normalized (g/h).*

Throughout the manuscript, MAFLD, MALFD (lines 82 and 111), NAFLD and NASH (line 214) are used instead of the recently accepted terminology - MASLD and MASH.

We thank the Reviewer for highlighting these inconsistencies in the abbreviations that are now corrected.

Figure 1F: Please add units for Y axis (Energy Expenditure)

The Energy Expenditure Y-axis has been corrected and now shows the units in kcal/h.

Fig 5C: The absolute levels of all plasma measurements should be included, at a minimum, as Supplementary Material.

The dataset of all measured plasma variables for each mouse is available as an Excel file in **Supplementary Table 1**.

Lines 165-167: The authors speculate that "Genes related to the mevalonate pathway, crucial for cholesterol synthesis were downregulated, likely as a protective response to reduce lipotoxicity". A more likely explanation would be negative feedback driven by SREBP2 suppression due to the overload of dietary cholesterol.

We agree that a more likely explanation is negative feedback mediated by SREBP2 suppression in response to cholesterol overload. This sentence has now been rewritten accordingly (**lines 200–205**).

REFERENCES:

1. Karp, C.L. Unstressing intemperate models: how cold stress undermines mouse modeling. *J Exp Med* **209**, 1069-74 (2012).
2. Cannon, B. and J. Nedergaard. Brown adipose tissue: function and physiological significance. *Physiol Rev* **84**, 277-359 (2004).
3. Fischer, A.W., B. Cannon, and J. Nedergaard. Optimal housing temperatures for mice to mimic the thermal environment of humans: An experimental study. *Mol Metab* **7**, 161-170 (2018).
4. Ganeshan, K. and A. Chawla. Warming the mouse to model human diseases. *Nat Rev Endocrinol* **13**, 458-465 (2017).
5. Gordon, C.J. The mouse thermoregulatory system: Its impact on translating biomedical data to humans. *Physiol Behav* **179**, 55-66 (2017).
6. Overton, J.M. Phenotyping small animals as models for the human metabolic syndrome: thermoneutrality matters. *Int J Obes (Lond)* **34 Suppl 2**, S53-8 (2010).
7. Vialard, F. and M. Olivier. Thermoneutrality and Immunity: How Does Cold Stress Affect Disease? *Front Immunol* **11**, 588387 (2020).
8. Stemmer, K., et al. Thermoneutral housing is a critical factor for immune function and diet-induced obesity in C57BL/6 nude mice. *Int J Obes (Lond)* **39**, 791-7 (2015).
9. Giles, D.A., et al. Thermoneutral housing exacerbates nonalcoholic fatty liver disease in mice and allows for sex-independent disease modeling. *Nat Med* **23**, 829-838 (2017).
10. John, L.M., et al. Housing-temperature reveals energy intake counter-balances energy expenditure in normal-weight, but not diet-induced obese, male mice. *Commun Biol* **5**, 946 (2022).
11. van der Velpen, V., et al. Sex-specific alterations in NAD+ metabolism in 3xTg Alzheimer's disease mouse brain assessed by quantitative targeted LC-MS. *J Neurochem* **159**, 378-388 (2021).
12. Teav, T., et al. Merged Targeted Quantification and Untargeted Profiling for Comprehensive Assessment of Acylcarnitine and Amino Acid Metabolism. *Anal Chem* **91**, 11757-11769 (2019).
13. Girish, V. and S. John, *Metabolic Dysfunction-Associated Steatotic Liver Disease (MAlphaSLD)*, in *StatPearls*. 2025: Treasure Island (FL).
14. Muzurovic, E., et al. Nonalcoholic Fatty Liver Disease and Cardiovascular Disease: a Review of Shared Cardiometabolic Risk Factors. *Hypertension* **79**, 1319-1326 (2022).
15. Steinberg, G.R., et al. Integrative metabolism in MASLD and MASH: Pathophysiology and emerging mechanisms. *J Hepatol* **83**, 584-595 (2025).
16. Feng, X., et al. Mechanism of Metabolic Dysfunction-associated Steatotic Liver Disease: Important role of lipid metabolism. *J Clin Transl Hepatol* **12**, 815-826 (2024).

17. Steinberg, G.R., A.C. Carpentier, and D. Wang. MASH: the nexus of metabolism, inflammation, and fibrosis. *J Clin Invest* **135**, (2025).
18. Sakuma, I., et al. Liver lipid droplet cholesterol content is a key determinant of metabolic dysfunction-associated steatohepatitis. *Proc Natl Acad Sci U S A* **122**, e2502978122 (2025).
19. Horn, C.L., et al. Role of Cholesterol-Associated Steatohepatitis in the Development of NASH. *Hepatol Commun* **6**, 12-35 (2022).
20. Bachmann, A.M., et al. Genetic background and sex control the outcome of high-fat diet feeding in mice. *iScience* **25**, 104468 (2022).
21. Benegiamo, G., et al. The genetic background shapes the susceptibility to mitochondrial dysfunction and NASH progression. *J Exp Med* **220**, (2023).
22. Arivazhagan, L., et al. Sex differences in murine MASH induced by a fructose-palmitate-cholesterol-enriched diet. *JHEP Rep* **7**, 101222 (2025).
23. Cheng, J.M., et al. Plasma concentrations of molecular lipid species in relation to coronary plaque characteristics and cardiovascular outcome: Results of the ATHEROREMO-IVUS study. *Atherosclerosis* **243**, 560-6 (2015).
24. Lemaitre, R.N., et al. Plasma Ceramides and Sphingomyelins in Relation to Heart Failure Risk. *Circ Heart Fail* **12**, e005708 (2019).
25. Bismuth, J., et al. Ceramide: a common pathway for atherosclerosis? *Atherosclerosis* **196**, 497-504 (2008).
26. Liu, C., et al. Cytokines: From Clinical Significance to Quantification. *Adv Sci (Weinh)* **8**, e2004433 (2021).
27. Norheim, F., et al. Genetic, dietary, and sex-specific regulation of hepatic ceramides and the relationship between hepatic ceramides and IR. *J Lipid Res* **59**, 1164-1174 (2018).
28. Hornemann, T., et al. The SPTLC3 subunit of serine palmitoyltransferase generates short chain sphingoid bases. *J Biol Chem* **284**, 26322-30 (2009).
29. Kovilakath, A., et al. SPTLC3 Is Essential for Complex I Activity and Contributes to Ischemic Cardiomyopathy. *Circulation* **150**, 622-641 (2024).
30. Montefusco, D., et al. SPTLC3 regulates plasma membrane sphingolipid composition to facilitate hepatic gluconeogenesis. *Cell Rep* **43**, 115054 (2024).
31. Visentin, M., et al. Dyslipidemic SPTLC3 Integrates Bile Acid-FXR Signaling with Sphingolipid Remodeling in MASLD. *bioRxiv*, (2025).
32. Price, T.R., et al. Identification of genetic drivers of plasma lipoprotein size in the Diversity Outbred mouse population. *J Lipid Res* **64**, 100471 (2023).
33. Hakim, A., et al. Genetic Variation in the Mitochondrial Glycerol-3-Phosphate Acyltransferase Is Associated With Liver Injury. *Hepatology* **74**, 3394-3408 (2021).
34. Spooner, M.H., et al. Time course of western diet (WD) induced nonalcoholic steatohepatitis (NASH) in female and male Ldlr-/- mice. *PLoS One* **18**, e0292432 (2023).
35. Manjarres, L., et al. Sex differences in the relationship between body composition and MASLD progression in a murine model of metabolic syndrome. *iScience* **28**, 111863 (2025).
36. Lee, C., et al. Formyl peptide receptor 2 determines sex-specific differences in the progression of nonalcoholic fatty liver disease and steatohepatitis. *Nat Commun* **13**, 578 (2022).
37. Frayn, K.N. Calculation of substrate oxidation rates in vivo from gaseous exchange. *J Appl Physiol Respir Environ Exerc Physiol* **55**, 628-34 (1983).
38. Lusk, G. ANIMAL CALORIMETRY: Twenty-Fourth Paper. ANALYSIS OF THE OXIDATION OF MIXTURES OF CARBOHYDRATE AND FAT. *Journal of Biological Chemistry* **59**, 41-42 (1924).

POINT TO POINT REPLY

We sincerely thank the Reviewers for taking the time to evaluate our revised manuscript and for providing their feedback. Responses to their comments and the corresponding changes in the revised manuscript are highlighted in blue.

Before addressing the reviewers' concerns, we would also like to inform the Editors and Reviewers that, while preparing the second revision of our manuscript, the Metabolomics Unit at the University of Lausanne (UNIL) informed us that the plasma amino acid analysis data provided was incorrect. The figure has been updated with the corrected data, which do not alter the main conclusions of the study (**Fig. S11D, plasma**). We would like to emphasize that this issue was beyond our control and all data have been carefully reviewed in collaboration with the facility.

REVIEWER COMMENTS

Reviewer #1 (Remarks to the Author):

The author has made comprehensive revisions. However, the author should supplement the study with additional protein quantification experiments to further substantiate the fibrotic changes, such as measuring the protein levels of αSMA, COL1A1, COL1A3, CTGF, and TIMP1. These experiments should be added.

In response to the Reviewer's suggestion, we measured the protein levels of fibrosis-associated markers in the liver by Western blot analysis, and these data have now been included as **Supplementary Figure 3**. While we appreciate the Reviewer's comment, we respectfully believe that further quantification of individual proteins was not necessary, as liver fibrosis has already been thoroughly characterized in our study using transcriptomic, proteomic (the gold standard for protein quantifications) and histological approaches (Sirius Red and α-SMA staining). Additionally, TIMP1 levels were measured in plasma. Furthermore, individual gene and protein expression data derived from the omics analyses are fully accessible and can be explored through our online app (https://lisp-lms.shinyapps.io/PWK_study_app/), as illustrated in **Supportive Image 1**. We believe these multi-layered datasets provide robust evidence of fibrosis without the need for additional individual protein assays.

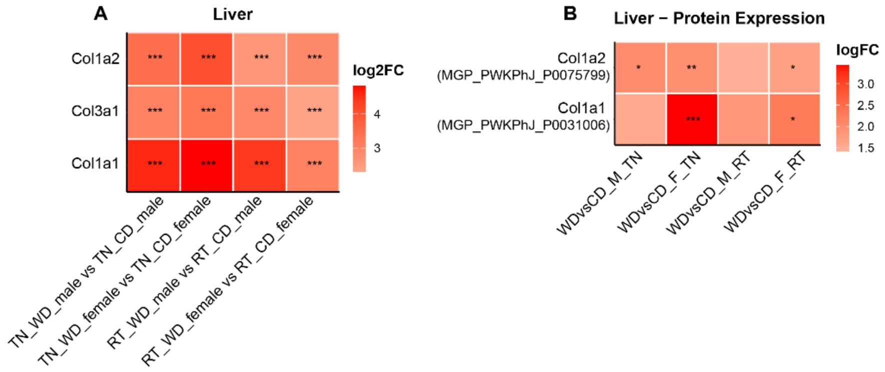

Supportive Image 1. *Liver fibrosis-related gene expression (A) and protein expression (B) visualized using our online app. Individual gene expression data are available at "Temperature Effect -> BRB-seq-> Gene Plots" and protein expression data at "Temperature Effect -> Proteomics-> Protein Plots". Images can be downloaded in various formats, such as png and pdf.*

Reviewer #3 (Remarks to the Author):

The authors have addressed most of the comments and acknowledged the key limitations of the study. The following issues remain and should be addressed:

1) The authors acknowledge that the absence of a direct comparison to other strains including C57BL/6J mice represents a key limitation of the current manuscript. Accordingly, because the study did not include analyses across other strains, statements suggesting superiority of the PWK/PhJ strain should be removed throughout the manuscript. Here are a couple of examples:

Abstract: "…male and female PWK/PhJ mice exhibit accelerated hepatic fibrosis and cardiac dysfunction…"

Conclusion: "…making them a preferred model to study the transition of MASLD to MASH"

Terms such as 'accelerated' or 'preferred' can only be used when conducting a comparative study.

As suggested by the Reviewer, we have toned down the language in the revised manuscript.

2) Although measuring indices of systemic inflammation is relatively straightforward, the authors did not perform such assessments in the revised manuscript. Analyses of inflammatory gene expression in the liver would not address this limitation. Notably, speculations regarding systemic inflammation remained in the manuscript without any supporting data: "Larger liver size (Fig. 2A) and higher cholesterol levels (Fig. S1B) in females may contribute to systemic inflammation and impair heart function".

As noted in the previous revision, detecting circulating pro-inflammatory cytokines and chemokines in plasma to assess systemic inflammation in MASH mouse model is challenging. This conclusion was based on measurements from our previous study [1], where these markers were undetectable or below the limit of detection. These data are presented in the below **Supporting Table 1.**

For this revision, we measured a panel of 11 cytokines, chemokines and other plasma factors, (KC (CXCL1), TNF-α, MIP-2 (CXCL2), IL-6, MCP-1 (CCL2), IL-1β (IL-1F2), IFN-γ, ICAM-1 (CD54), CXCL10 (IP-10/CRG-2), G-CSF and MMP9 using the Luminex platform. As expected from the outcome of our previous studies, these markers remained largely undetectable. ICAM-1 and MMP9 were the only two markers detected among the evaluated and have now been included in **Figure 1I**. The complete results from this experiment are provided in **Supplementary Table 1**, as we believe they may be valuable for the research community.

Accordingly, and as suggested by this reviewer, we have removed the statement regarding systemic inflammation from the manuscript (**Lines 167-168**), as these markers were undetectable in our samples.

3) Another statement that should be moderated, as it lacks experimental evidence including modulation of Sptlc3 in PWK/PhJ mice or other strains, is the following: "...the increased transcript levels of Sptlc3 and enhanced de novo synthesis are the primary drivers of ceramide and deoxy-ceramide accumulation in the liver, which could explain why PWK/PhJ is the most sensitive strain to MASH, developing fibrosis and mitochondrial dysfunction [21]."

As suggested by the Reviewer, we have toned down the statement in the revised manuscript (**Lines 357-362**).

4) The addition of liver histopathological analyses have strengthened the revised manuscript. However, the absence of histological and biochemical assessments to demonstrate cardiac dysfunction represents a major limitation, which weakens the study's conclusion regarding a dual model

Although additional histology analysis could provide supportive information, our study includes multi-layered functional and biochemical evidence of cardiac dysfunction. Echocardiography demonstrates increased LV mass with reduced EF and FS, while plasma analyses reveal metabolic alterations (elevated fasting glucose, insulin, HOMA-IR, LDL and HDL cholesterol, and TIMP1) and both plasma and heart metabolomics show elevated cardiotoxic sphingolipids liked to atherosclerotic plaques (SM, Cer 16:0). Together, these data provide in our opinion evidence for the MASH–cardiac dysfunction dual model. Furthermore, we did not collect heart tissue for histological analysis in this study; providing these data would require repeating all experiments, which we feel is beyond the scope of this manuscript.

Supporting Table 1. Plasma cytokines and chemokines involved in inflammation and immune regulation, Benegiamo G et al., 2023 [1].

Strain	ID	Diet	Sex	TNFα	CCL2	IL-6	IL-10	IFNγ
PWK/PhJ	HDP-002520	CD	m	< 3.251	< 210.576	< 29.547	< 12.469	< 12.593
PWK/PhJ	HDP-002521	CD	m	< 3.251	< 210.576	< 29.547	0	< 12.593
PWK/PhJ	HDP-002530	HFD	m	< 3.251	< 210.576	< 29.547	< 12.469	< 12.593
PWK/PhJ	HDP-002531	HFD	m	< 3.251	< 210.576	< 29.547	< 12.469	< 12.593
PWK/PhJ	HDP-002536	HFD	m	< 3.251	< 210.576	< 29.547	0	< 12.593
CAST/EiJ	HDP-002544	HFD	m	< 3.251	< 210.576	< 29.547	0	< 12.593
CAST/EiJ	HDP-002546	HFD	m	< 3.251	< 210.576	< 29.547	< 12.469	< 12.593
C57BL/6J	HDP-002577	CD	m	< 3.251	< 210.576	< 29.547	1	< 12.593
C57BL/6J	HDP-002578	CD	m	< 3.251	< 210.576	< 29.547	1	< 12.593
C57BL/6J	HDP-002579	CD	m	< 3.251	< 210.576	< 29.547	1	< 12.593
C57BL/6J	HDP-002580	HFD	m	< 3.251	< 210.576	< 29.547	4	< 12.593
C57BL/6J	HDP-002581	HFD	m	< 3.251	< 210.576	< 29.547	1	< 12.593
C57BL/6J	HDP-002582	HFD	m	< 3.251	< 210.576	< 29.547	0	< 12.593
C57BL/6J	HDP-002583	HFD	m	< 3.251	< 210.576	< 29.547	5	< 12.593
C57BL/6J	HDP-002584	HFD	m	< 3.251	< 210.576	< 29.547	1	< 12.593
CAST/EiJ	HDP-002600	CD	m	< 3.251	84	< 29.547	1	< 12.593
CAST/EiJ	HDP-002662	HFD	m	< 3.251	84	< 29.547	0	< 12.593
PWK/PhJ	HDP-002669	CD	m	< 3.251	< 210.576	< 29.547	1	< 12.593
C57BL/6J	HDP-003022	HFD	m	< 3.251	< 210.576	< 29.547	2	< 12.593
C57BL/6J	HDP-003023	HFD	m	< 3.251	< 210.576	< 29.547	1	< 12.593
C57BL/6J	HDP-003024	HFD	m	< 3.251	< 210.576	< 29.547	1	< 12.593
PWK/PhJ	HDP-003030	HFD	m	< 3.251	< 210.576	< 29.547	0	< 12.593
PWK/PhJ	HDP-003031	HFD	m	< 3.251	< 210.576	< 29.547	< 12.469	< 12.593
PWK/PhJ	HDP-003032	HFD	m	< 3.251	< 210.576	< 29.547	0	< 12.593
PWK/PhJ	HDP-003033	HFD	m	< 3.251	< 210.576	< 29.547	1	< 12.593
PWK/PhJ	HDP-003039	CD	m	< 3.251	< 210.576	< 29.547	< 12.469	< 12.593
PWK/PhJ	HDP-003040	CD	m	< 3.251	< 210.576	< 29.547	0	< 12.593
A/J	HDP-003050	CD	m	< 3.251	< 210.576	< 29.547	1	< 12.593
A/J	HDP-003051	CD	m	< 3.251	< 210.576	< 29.547	0	< 12.593
A/J	HDP-003052	CD	m	< 3.251	< 210.576	< 29.547	0	< 12.593

PWK/PhJ	HDP-003077	CD	m	< 3.251	< 210.576	< 29.547	0	< 12.593
C57BL/6J	HDP-003088	CD	m	< 3.251	84	< 29.547	1	< 12.593
C57BL/6J	HDP-003089	CD	m	< 3.251	< 210.576	< 29.547	1	< 12.593
C57BL/6J	HDP-003090	CD	m	< 3.251	84	< 29.547	2	< 12.593
C57BL/6J	HDP-003119	CD	m	< 3.251	< 210.576	< 29.547	1	< 12.593
C57BL/6J	HDP-003120	CD	m	< 3.251	< 210.576	< 29.547	0	< 12.593
A/J	HDP-003164	HFD	m	< 3.251	< 210.576	< 29.547	1	< 12.593
A/J	HDP-003165	HFD	m	< 3.251	< 210.576	< 29.547	1	< 12.593
A/J	HDP-003166	HFD	m	< 3.251	< 210.576	< 29.547	1	< 12.593
A/J	HDP-003167	HFD	m	< 3.251	< 210.576	< 29.547	1	< 12.593
A/J	HDP-003400	HFD	m	< 3.251	84	< 29.547	1	< 12.593
A/J	HDP-003404	CD	m	< 3.251	< 210.576	< 29.547	0	< 12.593
A/J	HDP-003408	CD	m	< 3.251	< 210.576	< 29.547	1	< 12.593
A/J	HDP-003409	CD	m	< 3.251	< 210.576	< 29.547	1	< 12.593
CAST/EiJ	HDP-003417	HFD	m	< 3.251	84	< 29.547	1	< 12.593
CAST/EiJ	HDP-003420	CD	m	< 3.251	84	< 29.547	1	< 12.593
CAST/EiJ	HDP-003421	CD	m	< 3.251	84	< 29.547	1	< 12.593
CAST/EiJ	HDP-003422	CD	m	< 3.251	< 210.576	< 29.547	1	< 12.593
129S1/SvImJ	HDP-003458	HFD	m	< 3.251	< 210.576	< 29.547	1	< 12.593
129S1/SvImJ	HDP-003459	HFD	m	< 3.251	< 210.576	< 29.547	0	< 12.593
129S1/SvImJ	HDP-003463	HFD	m	< 3.251	< 210.576	< 29.547	2	< 12.593
129S1/SvImJ	HDP-003464	CD	m	< 3.251	84	< 29.547	0	< 12.593
129S1/SvImJ	HDP-003473	CD	m	< 3.251	46	< 29.547	1	< 12.593
129S1/SvImJ	HDP-003474	CD	m	< 3.251	84	< 29.547	0	< 12.593
A/J	HDP-003478	HFD	m	< 3.251	< 210.576	< 29.547	1	< 12.593
CAST/EiJ	HDP-003516	HFD	m	< 3.251	< 210.576	< 29.547	1	< 12.593
DBA/2J	HDP-005026	CD	m	< 3.251	< 210.576	< 29.547	0	< 12.593
DBA/2J	HDP-005036	HFD	m	< 3.251	< 210.576	< 29.547	1	< 12.593
DBA/2J	HDP-005037	HFD	m	< 3.251	< 210.576	< 29.547	1	< 12.593
DBA/2J	HDP-005040	HFD	m	< 3.251	< 210.576	< 29.547	< 12.469	< 12.593
DBA/2J	HDP-005041	HFD	m	< 3.251	< 210.576	< 29.547	< 12.469	< 12.593
CAST/EiJ	HDP-005065	CD	m	< 3.251	< 210.576	< 29.547	< 12.469	< 12.593
CAST/EiJ	HDP-005067	CD	m	< 3.251	< 210.576	< 29.547	< 12.469	< 12.593
CAST/EiJ	HDP-005068	HFD	m	< 3.251	< 210.576	< 29.547	< 12.469	< 12.593
CAST/EiJ	HDP-005069	HFD	m	< 3.251	< 210.576	< 29.547	< 12.469	< 12.593
CAST/EiJ	HDP-005070	HFD	m	< 3.251	< 210.576	< 29.547	< 12.469	< 12.593
DBA/2J	HDP-005108	CD	m	< 3.251	< 210.576	< 29.547	< 12.469	< 12.593
DBA/2J	HDP-005109	CD	m	< 3.251	< 210.576	< 29.547	< 12.469	< 12.593
129S1/SvImJ	HDP-005296	HFD	m	< 3.251	< 210.576	< 29.547	< 12.469	< 12.593
129S1/SvImJ	HDP-005297	HFD	m	< 3.251	22	< 29.547	< 12.469	< 12.593
129S1/SvImJ	HDP-005298	HFD	m	< 3.251	< 210.576	< 29.547	< 12.469	< 12.593
129S1/SvImJ	HDP-005299	HFD	m	< 3.251	< 210.576	< 29.547	< 12.469	< 12.593
129S1/SvImJ	HDP-005355	CD	m	< 3.251	99	< 29.547	< 12.469	< 12.593
129S1/SvImJ	HDP-005356	CD	m	< 3.251	< 210.576	< 29.547	< 12.469	< 12.593
129S1/SvImJ	HDP-005357	CD	m	< 3.251	< 210.576	< 29.547	< 12.469	< 12.593
129S1/SvImJ	HDP-005358	CD	m	< 3.251	< 210.576	< 29.547	< 12.469	< 12.593
DBA/2J	HDP-005433	CD	m	< 3.251	< 210.576	< 29.547	< 12.469	< 12.593
DBA/2J	HDP-005434	CD	m	< 3.251	< 210.576	< 29.547	< 12.469	< 12.593
DBA/2J	HDP-005435	CD	m	< 3.251	< 210.576	< 29.547	< 12.469	< 12.593
DBA/2J	HDP-005436	HFD	m	< 3.251	< 210.576	< 29.547	< 12.469	< 12.593
WSB/EiJ	HDP-005462	HFD	m	< 3.251	< 210.576	< 29.547	< 12.469	< 12.593
WSB/EiJ	HDP-005463	HFD	m	< 3.251	< 210.576	< 29.547	< 12.469	< 12.593
WSB/EiJ	HDP-005464	HFD	m	< 3.251	99	< 29.547	< 12.469	< 12.593
DBA/2J	HDP-005476	HFD	m	< 3.251	99	< 29.547	< 12.469	< 12.593
DBA/2J	HDP-005477	HFD	m	< 3.251	< 210.576	< 29.547	< 12.469	< 12.593
WSB/EiJ	HDP-005607	HFD	m	< 3.251	99	< 29.547	< 12.469	< 12.593
WSB/EiJ	HDP-005612	CD	m	< 3.251	< 210.576	< 29.547	< 12.469	< 12.593
WSB/EiJ	HDP-005613	CD	m	< 3.251	< 210.576	< 29.547	< 12.469	< 12.593
WSB/EiJ	HDP-005614	CD	m	< 3.251	< 210.576	< 29.547	< 12.469	< 12.593

WSB/EiJ	HDP-005615	CD	m	< 3.251	< 210.576	< 29.547	< 12.469	< 12.593
WSB/EiJ	HDP-006070	CD	m	< 3.251	< 210.576	< 29.547	< 12.469	< 12.593
WSB/EiJ	HDP-006071	CD	m	< 3.251	< 210.576	< 29.547	< 12.469	< 12.593
WSB/EiJ	HDP-006072	HFD	m	< 3.251	< 210.576	< 29.547	< 12.469	< 12.593
WSB/EiJ	HDP-006251	HFD	m	< 3.251	< 210.576	< 29.547	< 12.469	< 12.593

REFERENCES:

1. Benegiamo, G., et al. The genetic background shapes the susceptibility to mitochondrial dysfunction and NASH progression. *J Exp Med* **220**, (2023).
